# Order parameters and phase transitions of continual learning in deep neural networks

**DOI:** 10.1073/pnas.2501899123

**Published:** 2026-02-06

**Authors:** Haozhe Shan, Qianyi Li, Haim Sompolinsky

**Affiliations:** ^a^Center for Brain Science, Harvard University, Cambridge, MA 02138; ^b^Program in Neuroscience, Harvard Medical School, Boston, MA 02115; ^c^Department of Computer Science and Zuckerman Institute, Columbia University, New York, NY 10027; ^d^Biophysics Graduate Program, Harvard University, Cambridge, MA 02138; ^e^Edmond and Lily Safra Center for Brain Sciences, Hebrew University, Jerusalem 9190401, Israel

**Keywords:** continual learning, deep neural network, catastrophic forgetting, anterograde interference, order parameters

## Abstract

Continual learning (CL) enables animals to learn new tasks without erasing prior knowledge. CL in artificial neural networks (NNs) is challenging due to catastrophic forgetting, where new learning degrades performance on older tasks. While various techniques exist to mitigate forgetting, theoretical insights into when and why CL fails in NNs are lacking. Here, we present a statistical-mechanics theory of CL in deep, wide NNs, which characterizes the network’s input–output mapping as it learns a sequence of tasks. It gives rise to order parameters (OPs) that capture how task relations and network architecture influence forgetting and anterograde interference, as verified by numerical evaluations. For networks with a shared readout for all tasks (single-head CL), the relevant-feature and rule similarity between tasks, respectively measured by two OPs, are sufficient to predict a wide range of CL behaviors on classic benchmark tasks. In addition, the theory predicts that increasing the network depth can effectively reduce interference between tasks, thereby lowering forgetting. For networks with task-specific readouts (multihead CL), the theory identifies a phase transition where CL performance shifts dramatically as tasks become less similar, as measured by another task-similarity OP. While forgetting is relatively mild compared to single-head CL across all tasks, sufficiently low similarity leads to catastrophic anterograde interference, where the network retains old tasks and interpolates new training data perfectly but completely fails to generalize new learning. Our results delineate important factors affecting CL performance and offer theoretical insights into common heuristics for mitigation of forgetting.

## Significance

Continual learning (CL), the ability to learn new tasks without forgetting existing ones, is one of the greatest challenges in AI. Our work provides an analytically tractable theory that captures some key phenomena of CL in deep, wide neural networks. We highlight several “order parameters” that measure the similarity between tasks and show that they can be highly predictive of CL behaviors on classic benchmark tasks. Strikingly, we identify a set of phase transitions where the network’s CL ability changes abruptly with the order parameter. Our results provide quantitative understanding of how CL ability depends on task relations, network architectures, and learning procedures.

Continual learning (CL), the capability to acquire and refine knowledge and skills over time, is fundamental to how animals survive in a nonstationary world. As an animal learns and performs many tasks, CL allows it to leverage previous learning to help learn a new task while retaining the ability to perform old ones. In artificial neural networks (NN), developing such abilities has been challenging. Artificial NNs especially struggle with catastrophic forgetting, where learning a new task overwrites existing information and dramatically degrades performance on previously learned tasks (1–4). This problem is so prevalent and severe in machine learning (ML) that it has become one of the biggest challenges for developing human-level artificial general intelligence (5–7). Despite also relying on NNs for computation, the brain clearly does not suffer from catastrophic forgetting to nearly the same extent (7). Not only does this offer an “existence proof” of successful CL in NNs (3, 7, 8), it also raises intriguing questions about mechanisms underlying CL in the brain. A wide range of possible underpinnings, ranging from memory reactivation (9), synaptic stabilization (10, 11), to representational drift (12), have been proposed. However, their specific contributions to CL in the brain are not well understood.

Engineering CL in ML and understanding its mechanisms in the brain both suffer from a lack of theoretical understanding of the problem in NNs. The challenges stem from two interconnected issues: 1) developing an analytical understanding of the learning process of CL in NNs and 2) characterizing and quantifying the diverse types of task relations and their impacts on forgetting. Recently, progress on the first challenge has been made by analyzing simplified cases where the network is a shallow linear network (13–17), or equivalently so via the neural tangent kernel (NTK) approximation (18, 19). However, these approaches fail to account for the realistic and common scenario of NNs having multiple readouts dedicated to different tasks. Other studies have addressed task-dedicated readouts but focused on networks with only a single hidden layer and a limited number of neurons (20–22), limiting their relevance to real-world NNs with typically hundreds of hidden units in each layer. Moreover, many of these studies (13, 14, 16, 17, 20–23) rely on fully synthetic datasets, limiting the generalizability of their conclusions across diverse datasets and task relations.

**Fig. 1.**
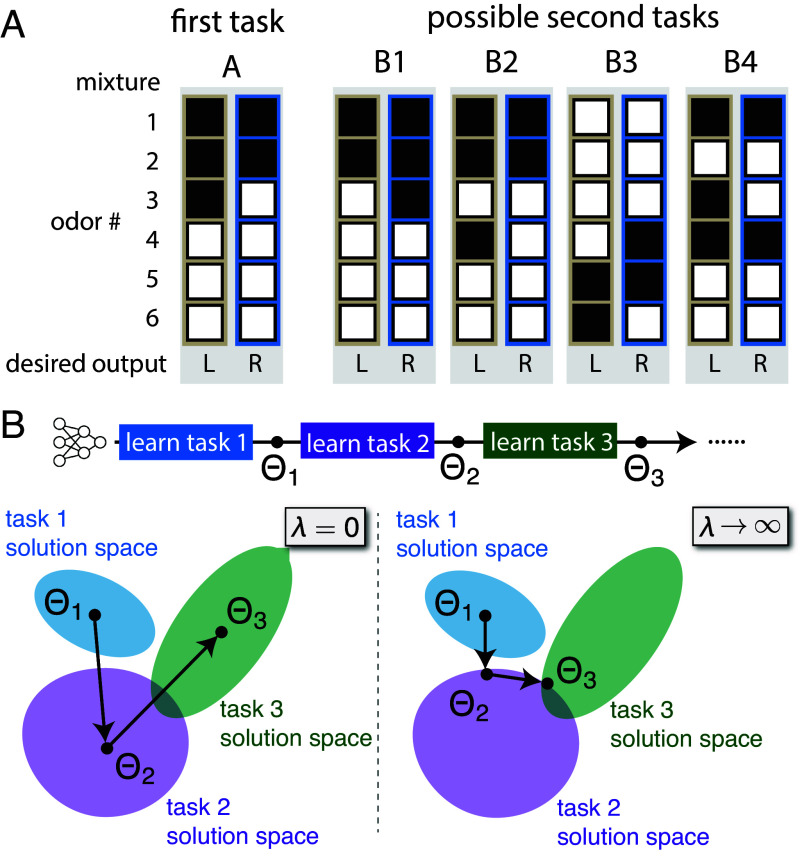
Different types of task relations in CL and the weight space schematics of the Gibbs formulation. (*A*) Hypothetical odor-mixture classification tasks demonstrating various possible relations between two supervised-learning tasks. Each task requires classification of two odor mixtures (each column is a mixture; black/white squares indicate present/absent odors). The subject needs to respond “L” or “R” depending on the presented mixture. The first task is A; possible second tasks B1, B2, B3, and B4 exhibit different relations to A. Tasks can have highly overlapping odors (A vs. B1, A vs. B2), partially overlapping odors (A vs. B4), or entirely different odors (A vs. B3). Among these odors some are relevant for the task (e.g. odor 3 for task A) while others are not (e.g. odor 1, 2 for task A). Tasks can also share these relevant odors (A vs. B1, A vs. B4) or have different relevant odors (A vs. B2). Furthermore, with the same relevant odors, the task rules can be the same (A vs. B4) or reversed (A vs. B1). It is crucial to quantify these different aspects of relations and identify their impact on CL performance. (*B*) Weight-space schematics showing the Gibbs formulation of CL. Each dataset defines a space of solutions where the training loss is zero. The network learns the first task by sampling from its space of solutions. For subsequent tasks, the network assumes different solutions depending on the regularization strength (λ). At λ=0, learning of each task samples from its corresponding space of solutions independent of previous learning. At the other extreme of λ→∞, learning chooses the solution closest to the weights sampled while learning the previous task. These schematics assume β→∞.

The second challenge involves systematically characterizing task relations, as illustrated by the hypothetical odor-mixture classification tasks in Fig. 1*A*. Task relations can vary depending on the overlap of the odors, whether overlapping odors are relevant to classification, and whether task rules are similar—different aspects of relations may well have diverging impacts on CL performance. This underscores the need for systematic, theory-motivated quantification of CL-relevant aspects of task relations. While recent work has proposed metrics of task relations (18, 24), whether and how they may be used to predict CL performance remains unclear.

In this work, we utilize a Gibbs formulation of CL and use tools from statistical physics to develop a theory of CL in deep, wide NNs (25, 26). Critically, our results require minimal data assumptions, enabling analysis across a broad range of tasks. The theory connects the degree of forgetting and anterograde interference during CL to task relations, the NN’s architecture, and hyperparameters of the learning process. A key contribution is the identification of scalar order parameters (OPs) quantifying different aspects of task relations that are predictive of CL performance on classic benchmark task sequences, as summarized in Table 1. For NNs without task-specific readouts, our theory identifies two critical OPs: one measuring relevant-feature similarity and another quantifying rule similarity. For networks with task-dedicated readouts, we introduce a third OP that captures overall task similarity. We also uncover three distinct CL regimes determined by this task-similarity OP and the ratio of training data to network width. While task-dedicated readouts generally reduce forgetting, sequentially learning dissimilar tasks can lead to “catastrophic anterograde interference,” where previous learning causes overfitting to the latest task. Our results offer a rigorous and predictive framework for understanding CL in deep NNs, highlighting measurable task and architecture factors that influence performance. Finally, we discuss the broader implications of our findings for understanding the neuroscience of CL.

**Table 1.** Definitions of the task relation OPs

Scenario	OP	Definition
Single-head	$\gamma_{\text{feature}}$	$P^{-1}\mathrm{Tr}\left(P_{1}P_{2}\right)$
	$\gamma_{\text{RF}}$	$V_{2}^{⊤}P_{12}V_{2}$
	$\gamma_{\text{rule}}$	$V_{2}^{⊤}P_{12}V_{1}$
Multihead	$\gamma_{\text{sim}}$	$\gamma_{\text{feature}}+\cos\left(V_{1},V_{2}\right)-V_{1}^{⊤}P_{2}V_{1}/\left|\right|V_{1}{\left|\right|}^{2}$

## 1. Gibbs Framework of CL in Deep Neural Networks

We studied a task-based CL setting (27) where the network learns a sequence of T tasks, respectively represented by training datasets $D_{1},...,D_{T}$ of identical size. $D_{t}\equiv \left(X_{t}\in R^{P\times N_{0}},Y_{t}\in R^{P}\right)$, where P is the number of examples per task and $N_{0}$ is the input dimensionality. Each row of $X_{t}$, $x_{t}^{\mu }$, is an input example with its corresponding label given by the μ-th element of $Y_{t}$, $y_{t}^{\mu }$. While learning task t, the network accesses $D_{t}$ but not the other datasets. We use “at time t” to refer to the state of the network after sequentially learning $D_{1}$ through $D_{t}$.

We first considered the simplest architecture: a multilayer perceptron where all weights are shared across tasks [“single-head” CL (28, 29)]. The network has L fully connected hidden layers, each containing N nonlinear neurons, assumed to be ReLU for concreteness. The network load is denoted α≡P/N. The input–output mapping of the network at time t is given by

[1] $$\begin{array}{c} f_{t}\left(x\right)\equiv \frac{1}{\sqrt{N}}a_{t}\cdot \Phi \left(W_{t},x\right), \end{array}$$

where $a_{t}\in R^{N}$ and $W_{t}$ are the readout and hidden-layer weights at time t, respectively. $\Phi \left(W_{t},x\right)\in R^{N}$ is the activation vector in the last hidden layer for input $x\in R^{N_{0}}$. The more complex “multihead” CL scenario, where the network utilizes task-specific readouts, is introduced and studied later.

We assume that learning $D_{t\geq 2}$ involves selecting the weights $\Theta_{t}\equiv \left\{a_{t},W_{t}\right\}$ according to a cost function

[2] $$\begin{array}{cc} & E\left(\Theta_{t}|\Theta_{t-1},D_{t}\right)\equiv \frac{1}{2}\sum_{\mu =1}^{P}{\left(f_{t}\left(x_{t}^{\mu }\right)-y_{t}^{\mu }\right)}^{2}\\ & +\frac{1}{2}\beta^{-1}\sigma^{-2}{∥\Theta_{t}∥}^{2}+\frac{1}{2}\beta^{-1}\lambda {∥\Theta_{t}-\Theta_{t-1}∥}^{2}. \end{array}$$

The first term measures the error of $f_{t}$ on $D_{t}$. The second term acts as regularization that favors weights with small norm, which is known to encourage good generalization (30). The third term is a perturbation penalty that favors small weight changes relative to $\Theta_{t-1}$, a natural strategy for mitigating forgetting (6, 10, 11, 31). β>0 denotes the inverse temperature, controlling how well $f_{t}$ interpolates $D_{t}$. $\sigma^{-2}>0$ and λ≥0 respectively scale the $L_{2}$ regularization and the perturbation penalty. The cost function for learning $D_{1}$, $E\left(\Theta_{1}|D_{1}\right)$, has the same form but without the penalty.

Learning at each stage is modeled as a posterior distribution of $\Theta_{t}$ conditioned on $\Theta_{t-1}$, $P\left(\Theta_{t}|\Theta_{t-1},D_{t}\right)\propto \;\exp\left(-\beta E\left(\Theta_{t}|\Theta_{t-1},D_{t}\right)\right)$. This distribution defines a Markovian transition from $\Theta_{t-1}$ to $\Theta_{t}$, controlled by $D_{t}$. Multiplying all such transition matrices for t≥2 and the posterior of learning the first task, $P\left(\Theta_{1}|D_{1}\right)\propto \;\exp\left(-\beta E\left(\Theta_{1}|D_{1}\right)\right)$, yields a joint posterior over $\Theta_{1},...,\Theta_{T}$ (16, 17, 32). This posterior fully describes how the network evolves during CL, from which various statistics can be calculated. We focus on overparameterized NNs (33) in the β→∞ limit. In this case, there is a large space of $\Theta_{t}$ that perfectly interpolates the dataset $D_{t}$ (Fig. 1*B*). At λ=0, there is no coupling between weights from different times, and the network has no memory of previous tasks. On the other hand, at λ→∞, the network makes the minimum perturbation to weights that is required to interpolate $D_{t}$ (34). Performance of the network on some dataset $D=\left(X,Y\right)$ at time t is measured by averaging the normalized mean-squared-error (MSE) loss,

[3] $$\begin{array}{c} L\left(f_{t},D\right)\equiv \sum_{\mu }{\left(f_{t}\left(x^{\mu }\right)-y^{\mu }\right)}^{2}/{∥Y∥}^{2}, \end{array}$$

over the posterior of $\Theta_{t}$, denoted $\left\langle L\left(f_{t},D\right)\right\rangle$.

## 2. Networks Using a Readout Shared Across Tasks

Our theory of single-head CL, exact in the infinite-width limit of N→∞,α→0, allows analytical evaluations of $\left\langle L\left(f_{t},D\right)\right\rangle$ for arbitrarily long task sequences (*SI Appendix*, Eqs. **S16** and **S17**). For intuition, we here discuss a naively simplified version of the full theory that 1) nevertheless reproduces the key qualitative behaviors of the full theory (*SI Appendix*, Fig. S1) and 2) provides simple geometric intuitions of task relations. We also focus our discussion here on the case where forgetting is minimized by taking λ→∞ and σ≪1 (*SI Appendix*, section 3.A.1).

### 2.1. Task-Relation Order Parameters (OPs).

The naive simplification, which is not required for the full theory, is motivated by the well-known observation that learning in networks in the infinite-width limit tends to induce only small modifications of the hidden weight matrices (35). In the simplified theory, we neglect learning-induced changes to the hidden-layer weights and assume them to be fixed at $W_{0}∼N\left(0,\sigma^{2}\right)$ throughout learning. Thus, CL in the network can be viewed as solely learning the readout from a fixed feature layer. We denote the training data of the two tasks as ${\left\{\left(X_{i},Y_{i}\right)\right\}}_{i\leq 2}$, and the features at the L-th hidden layer as $X_{i}^{L}\in R^{P\times N}$, where the μ-th row is given by $\Phi (W_{0},x_{i}^{\mu })$. Additionally, we assume the two tasks to have a “symmetric” relation (*SI Appendix*, section A.2). In this case, short-term forgetting, defined as the error on the training data of task 1 after learning two tasks ($F_{2,1}$), adopts a simple form:

[4] $$\begin{array}{c} F_{2,1}=2\left(\gamma_{\text{RF}}-\gamma_{\text{rule}}\right), \end{array}$$

where

[5] $$\begin{array}{cc} \gamma_{\text{RF}} & \equiv V_{2}^{⊤}P_{12}V_{2} \end{array}$$

[6] $$\begin{array}{cc} \gamma_{\text{rule}} & \equiv V_{2}^{⊤}P_{12}V_{1}. \end{array}$$

$P_{12}$ and $P_{i=1,2}$ are N×N matrices,

[7] $$\begin{array}{cc} P_{12} & \equiv \frac{1}{2N}\left(P_{2}{\left(X_{1}^{L}\right)}^{⊤}X_{1}^{L}P_{2}+P_{1}{\left(X_{2}^{L}\right)}^{⊤}X_{2}^{L}P_{1}\right) \end{array}$$

[8] $$\begin{array}{cc} P_{i} & \equiv {\left(X_{i}^{L}\right)}^{⊤}{\left[X_{i}^{L}{\left(X_{i}^{L}\right)}^{⊤}\right]}^{-1}X_{i}^{L} \end{array}$$

and $V_{i=1,2}$ are N-dimensional vectors,

[9] $$\begin{array}{c} V_{i}\equiv \sqrt{N}{\left(X_{i}^{L}\right)}^{⊤}{\left[X_{i}^{L}{\left(X_{i}^{L}\right)}^{⊤}\right]}^{-1}Y_{i}/\left|\right|Y_{i}\left|\right|. \end{array}$$

**Fig. 2.**
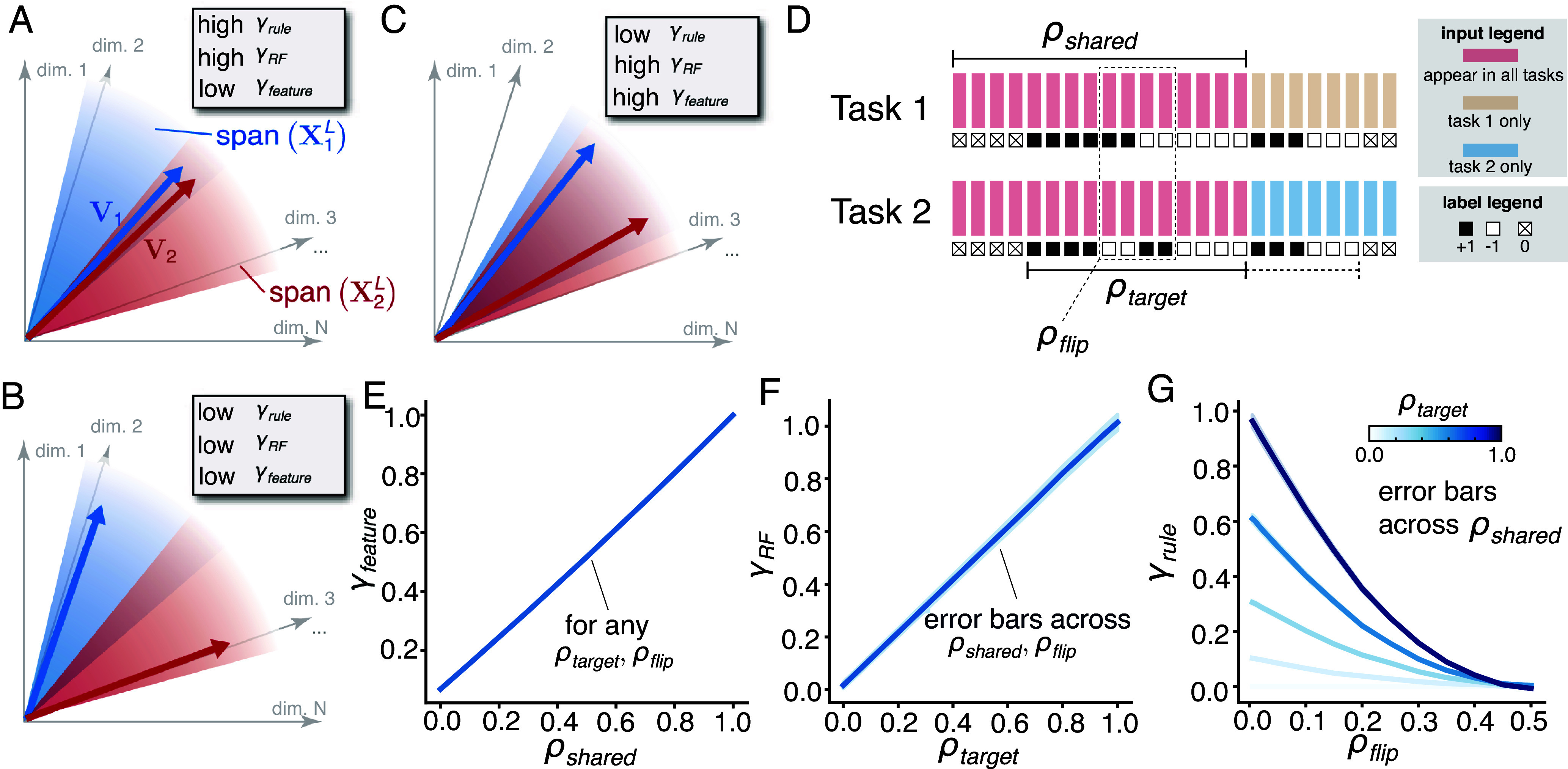
Schematics of the OPs and the target-distractor task. (*A*–*C*) Schematics of the OPs. The input features of each task span a P-dimensional subspace in the N dimensional feature space. $\text{span}(X_{1}^{L})$ (shown in blue) denotes the space spanned by task 1 input features at the L-th layer, while $\text{span}(X_{2}^{L})$ (shown in red) denotes the space spanned by task 2 input features at the L-th layer. $V_{1}$ and $V_{2}$ are the rule vectors of task 1 and task 2, and by definition lie in $\text{span}(X_{1}^{L})$ and $\text{span}(X_{2}^{L})$ respectively. $\gamma_{\text{RF}}$ measures how much the rule vectors project onto the shared feature dimensions. In (*A* and *C*), both rule vectors fully lie in the shared subspace and $\gamma_{\text{RF}}$ is high. In contrast, in (*B*) the rule vectors are away from the shared subspace, thus $\gamma_{\text{RF}}$ is small. $\gamma_{\text{rule}}$ measures the similarity between the projection of the rule vectors on to the shared feature dimensions. In a $\gamma_{\text{rule}}$ is high and in (*B* and *C*) $\gamma_{\text{rule}}$ is low. $\gamma_{\text{feature}}$ measures the degree of overlap between the shared feature dimensions and is low in (*A* and *B*) but high in (*C*). (*D*) Schematics of the target-distractor task. Each task consists of a set of P images (rectangles) from CIFAR-100, assigned labels ±1 or 0 (squares). $\rho_{\text{shared}}$ controls the ratio of shared images between two tasks. $\rho_{\text{target}}$ controls the ratio of images with ±1 labels that are shared between the tasks. For the shared images, some of the labels are flipped between the tasks, and $\rho_{\text{flip}}$ controls the ratio of the images with flipped labels. Varying these parameters allows us to explore the full range of the OPs. (*E*–*G*) Controlling the 3 OPs with the target-distractor task. $\gamma_{\text{feature}}$ depends only on $\rho_{\text{shared}}$ (*E*), as $\rho_{\text{shared}}$ increases, the overlap between the shared feature subspaces increases, resulting in higher $\gamma_{\text{feature}}$. $\gamma_{\text{RF}}$ depends mainly on $\rho_{\text{target}}$ (*F*). As $\rho_{\text{target}}$ increases, the rule vectors project more onto the shared feature dimensions, thus $\gamma_{\text{RF}}$ increases. $\gamma_{\text{rule}}$ is tuned by both $\rho_{\text{target}}$ and $\rho_{\text{flip}}$ (*G*). $\rho_{\text{target}}$ sets an upper bound of $\gamma_{\text{rule}}$, for a fixed $\rho_{\text{target}}$, $\gamma_{\text{rule}}$ decreases with $\rho_{\text{flip}}$. At $\rho_{\text{flip}}=0.5$ about half of the labels are flipped, and $\gamma_{\text{rule}}$ goes to 0. In both (*F* and *G*), error bars represent a uniform grid of 5 $\rho_{\text{shared}}$ values in [0.1,0.9] crossed with 11 values of $\rho_{\text{flip}}$ or $\rho_{\text{target}}$ (respectively) in [0,1].

These expressions allow us to describe task relations in terms of the geometry in the network’s N-dimensional feature space. $P_{i}$ projects vectors in this space onto the P-dimensional subspace spanned by the P training examples of task i, $X_{i}^{L}$. $P_{12}$ represents the projection of the input features of one task onto those of the other task. $V_{i}$ is the normalized readout from the top hidden layer of the network, generated when learning $\left(X_{i},Y_{i}\right)$ alone. We refer to it as the rule vector since it fully characterizes the learned input–output rule of each task. The two terms in Eq. **4** have interesting geometrical interpretations in the feature space, as illustrated in Fig. 2*A*–*C*. The first term, denoted as $\gamma_{\text{RF}}$, measures how much the rule vectors of individual tasks project onto the shared input feature subspace of both tasks. Intuitively, it corresponds to the similarity of the input features which are relevant to the task rules, and thus is referred to as the relevant-feature (RF) similarity. The second term, $\gamma_{\text{rule}}$, measures the similarity between the two task rule vectors projected onto the shared input feature subspace, and thus is referred to as the rule similarity. We note that both $\gamma_{\text{RF}}$ and $\gamma_{\text{rule}}$ depend on the target outputs and the input features. It is also instructive to define a third OP, $\gamma_{\text{feature}}\equiv P^{-1}\mathrm{Tr}\left(P_{1}P_{2}\right)$, which measures the degree of overlap between the input feature subspaces. It is thus a similarity metric between the input feature vectors, independent of the target outputs. By definition, they are algebraically bounded: $\gamma_{\mathrm{RF}}\geq 0$, $\gamma_{\text{rule}}\in \left[-\gamma_{\text{RF}},\gamma_{\text{RF}}\right]$ and $\gamma_{\text{features}}\in \left[0,1\right]$. Under reasonable assumptions (*SI Appendix*, section 3.A.2) we further have $\gamma_{\text{RF}}\in \left[0,1\right]$ and $\gamma_{\text{rule}}\in \left[0,\gamma_{\text{RF}}\right]$, which empirically hold for our results throughout this paper.

In summary, the quantity $\gamma_{\text{RF}}-\gamma_{\text{rule}}$ provides a direct measure of short-term forgetting (Eq. **4**), which we hereafter refer to as the “conflict” between two tasks. Conflict can be small under two scenarios. The first occurs when both $\gamma_{\text{RF}}$ and $\gamma_{\text{rule}}$ are large but close in value, indicating that the tasks have similar relevant features and similar rules. The second is when both OPs are small, corresponding to the case where the tasks have dissimilar relevant features and rules. In contrast, two tasks would have high conflict if they share a lot of the relevant features (high $\gamma_{\text{RF}}$) but use different rules (low $\gamma_{\text{rule}}$).

### 2.2. Exploring the OPs with Target-Distractor Task Sequences.

We next sought to understand how the 3 OPs are tied to the severity of forgetting. We began by studying them in the setting of task sequences with parametrically controllable input distributions and task rules. We constructed “target-distractor task sequences” (full details in *SI Appendix*, section 5), where each task consists of a set of P randomly selected stimuli from a large pool of stimuli, e.g., P images from CIFAR-100 (36), with random labels ±1 or 0 for each task (Fig. 2*D*). A subset of the inputs is shared across all tasks in the sequence, whereas other inputs are unique to each task. The parameter $\rho_{\text{shared}}\in \left[0,1\right]$ represents the proportion of shared inputs among all P inputs across all tasks in the sequence and controls $\gamma_{\text{feature}}$ (Fig. 2*E*). Another parameter $\rho_{\text{target}}\in \left[0,1\right]$ represents the proportion of shared inputs among the inputs with ±1 labels across all tasks; varying $\rho_{\text{target}}$ controls $\gamma_{\mathrm{RF}}$ (Fig. 2*F*). For the shared images, part of the ±1 labels are flipped between tasks, and $\rho_{\text{flip}}\in \left[0,0.5\right]$ controls the ratio of images with flipped labels between tasks on average. It thereby affects how consistent the rules for different tasks are, as reflected in $\gamma_{\text{rule}}$ (Fig. 2*G*). Therefore, through varying $\left(\rho_{\text{shared}},\rho_{\text{target}},\rho_{\text{flip}}\right)$, we can explore the full range of the 3 OPs and elucidate their respective roles in forgetting.

**Fig. 3.**
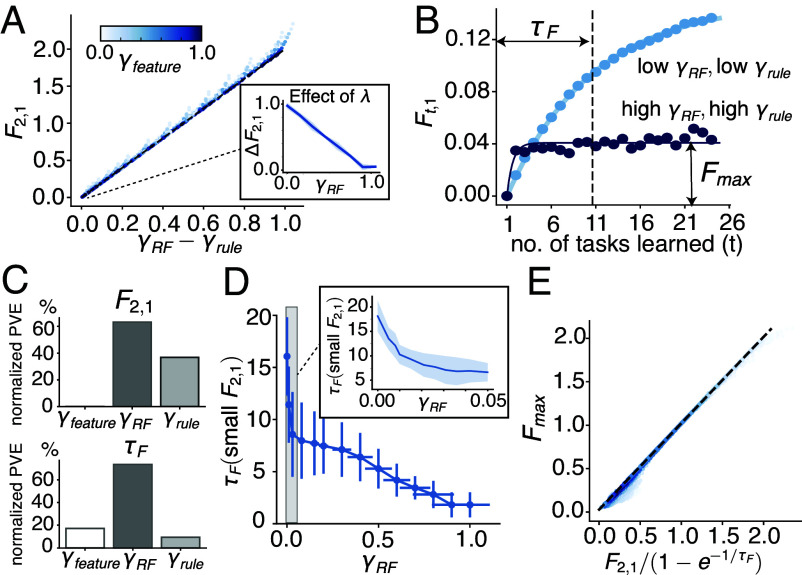
OPs predict short-term and long-term forgetting behaviors in target-distractor sequences. (*A*) Forgetting on the training data of the first task after learning two tasks ($F_{2,1}$) is accurately predicted by 2($\gamma_{\text{RF}}-\gamma_{\text{rule}})$, and does not depend on $\gamma_{\text{feature}}$ (represented by the color of the points). Each point represents a target-distractor task sequence with a different set of $\left(\rho_{\text{target}},\rho_{\text{shared}},\rho_{\text{flip}}\right)$. *Inset*: $\Delta F_{2,1}$ measures the effect of the regularizer. When $F_{2,1}$ is small (<0.05), $\gamma_{\text{RF}}$ and $\gamma_{\text{rule}}$ are close, the effect of the regularizer decreases as $\gamma_{\text{RF}}$ (and $\gamma_{\text{rule}}$) increases, i.e., as the tasks become more similar. (*B*) Long-term forgetting in a task sequence can be approximated by an exponential relaxation process, where $F_{\text{max}}$ denotes its asymptote as t→∞, and $\tau_{F}$ denotes its time constant (*SI Appendix*, section 7). We show two examples, one for dissimilar tasks (low $\gamma_{\text{RF}}$, low $\gamma_{\text{rule}}$) with relatively large $\tau_{F}$ and $F_{\text{max}}$, and the other for similar tasks (high $\gamma_{\text{RF}}$, high $\gamma_{\text{rule}}$), with relatively small $\tau_{F}$ and $F_{\text{max}}$. (*C*) Normalized PVE (proportion of variance explained, 37) of $F_{2,1}$ and $\tau_{F}$ by the 3 OPs (*SI Appendix*, section 5). $F_{2,1}$ depends on $\gamma_{\text{rule}}$ and $\gamma_{\text{RF}}$ and is independent of $\gamma_{\text{feature}}$, consistent with (*A*). $\tau_{F}$ mainly depends on $\gamma_{\text{RF}}$ and weakly depends on $\gamma_{\text{rule}}$ and $\gamma_{\text{feature}}$. (*D*) For tasks where $F_{2,1}$ is small (<0.05), $\tau_{F}$ decreases as $\gamma_{\text{RF}}$ increases. *Inset*: zoomed-in region of $\gamma_{\text{RF}}<0.05$ highlights the fast decrease of $\tau_{F}$ for small $\gamma_{\text{RF}}$. Data are binned by $\gamma_{\text{RF}}$, error bars are SDs across data points within the same bin. (*E*) $F_{\text{max}}$ can be accurately predicted given $F_{2,1}$ and $\tau_{F}$. Each point represents a target-distractor task sequence with a different set of $\left(\rho_{\text{target}},\rho_{\text{shared}},\rho_{\text{flip}}\right)$. The color represents the density of points. All $F_{t,1}$ and the corresponding OPs are averaged across 40 random seeds used for generating data. See *SI Appendix*, section 5 for detailed parameters.

As expected from Eq. **4**, $F_{2,1}$ is captured by the conflict (Fig. 3*A*) and is independent of $\gamma_{\text{feature}}$ (Fig. 3*C*, *Top* panel). We also measured the effect of λ by defining $\Delta F_{2,1}\equiv F_{2,1}-F_{2,1}\left(\lambda =0\right)$, where $F_{2,1}\left(\lambda =0\right)$ denotes forgetting on the first task without regularization (Fig. 3*A*, *Inset*). For tasks with low conflict, the effect of regularization on mitigating forgetting decreases as the tasks become more similar, as measured by $\gamma_{\text{RF}}$ (since the conflict is small, $\gamma_{\text{rule}}$ is close to $\gamma_{\text{RF}}$ for these tasks).

### 2.3. Long-Term Forgetting.

We next studied forgetting after learning sequences of multiple tasks, which we refer to as long-term forgetting. For simplicity, hereafter, we assume that all tasks in each sequence have identical pairwise relations; otherwise, task relations in a sequence of T tasks would require at least characterizing all T(T−1)/2 pairs. For such sequences, we empirically observed that forgetting of the first task ($F_{t,1}$) increases over time approximately as an exponential relaxation process, $F_{t,1}\approx F_{\text{max}}\left(1-e^{-\left(t-1\right)/\tau_{F}}\right)$. We thus characterized long-term forgetting by its time constant $\tau_{F}$ and long-time asymptote $F_{\max}$ (Fig. 3*B*). Interestingly, task sequences can have similar short-term forgetting but very different long-term forgetting behaviors (Fig. 3*B*). This suggests that $\tau_{F}$ and $F_{\text{max}}$ depend on the OPs in ways that differ from short-term forgetting ($F_{2,1}$). We analyzed the variance of $\tau_{F}$ explained by the 3 OPs (Fig. 3*C*, *Bottom* panel). $\tau_{F}$ mainly depends on $\gamma_{\text{RF}}$, and weakly depends on the other two OPs.

Since $F_{t,1}$ can only be relatively mild when $F_{2,1}$ is small, we are particularly interested in long-term forgetting at small $F_{2,1}$. For task sequences with small $F_{2,1}$ ($F_{2,1}<0.05$), $\tau_{F}$ decreases with $\gamma_{\text{RF}}$ (Fig. 3*D*). The decrease is very fast around $\gamma_{\text{RF}}$ close to 0 (Fig. 3*D*, *Inset*), and slows down afterward. Finally, since the exponential fit of $F_{t,1}$ is remarkably accurate across all parameters of the target-distractor task, characterzing the relation between the OPs and $F_{2,1}$ and $\tau_{F}$ also characterizes $F_{\text{max}}$ (Fig. 3*E*).

In summary, our results suggest that while short-term forgetting is small as long as the tasks have low conflict (small $\gamma_{\text{RF}}-\gamma_{\text{rule}}$), long-term forgetting can still vary depending on the similarity (magnitude of $\gamma_{\text{RF}}$ and $\gamma_{\text{rule}}$) between tasks. Forgetting tends to accumulate slowly for dissimilar tasks over time but quickly rises and plateaus for similar tasks (smaller $\tau_{F}$). On the other hand, neither short-term nor long-term forgetting depends on $\gamma_{\text{feature}}$, suggesting that a task-relation metric that includes only the input features is likely not informative of CL performance. Instead, it is crucial to consider the interaction between the task rules and the input features.

### 2.4. The Effect of Depth on Forgetting in Benchmark Sequences.

Having varied the task relations to explore the entire OP space using the target-distractor task sequences, we next studied the effects of the network depth, L. L affects CL performance by modifying $X_{i}^{L}$. To study the effect of L on general data, we analyzed the forgetting on several benchmark task sequences in NNs with different depths (L=1,3,5,7,9). Following standard practices, we created each sequence by applying a generation protocol (specified below) to a multiway classification dataset (“source datasets”): MNIST (38), EMNIST (39), Fashion-MNIST (40), or CIFAR-100. To generate long task sequences (T≫2) for the long-term forgetting analysis, we used the split protocol on EMNIST and CIFAR-100 and the permutation protocol on all source datasets, where a higher “permutation ratio” corresponds to less similar inputs between tasks (31) (see *SI Appendix*, section 6 for further details).

**Fig. 4.**
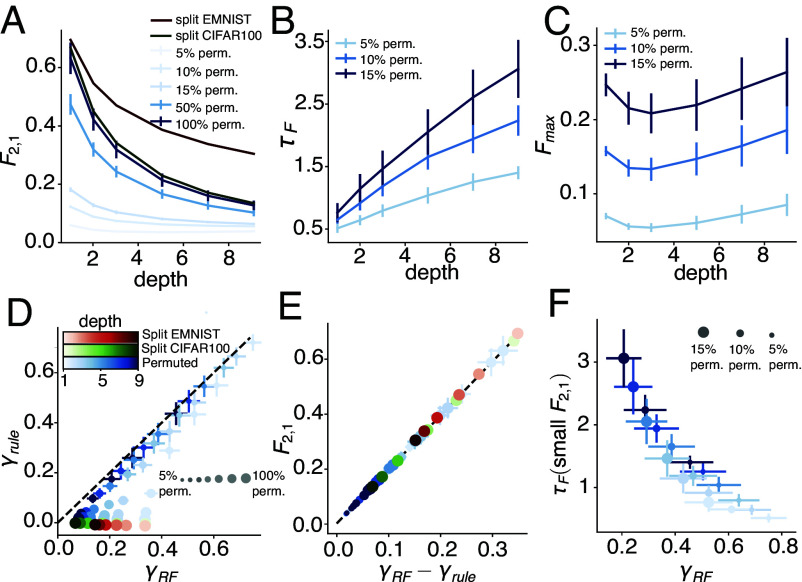
Forgetting in benchmark task sequences and the effect of depth. (*A*–*C*) The effect of depth on short-term ($F_{2,1}$) and long-term ($\tau_{F}$, $F_{\text{max}}$) forgetting on benchmark sequences. For permutation sequences, we averaged over source datasets including MNIST, EMNIST, Fashion-MNIST, and CIFAR as their behaviors are similar, and error bars are SEs across the source datasets. For split sequences, we show separately split EMNIST and split CIFAR-100 sequences as their behaviors are more different. In all cases, $F_{2,1}$ decreases with depth (*A*). We look at long-term forgetting only in sequences with small $F_{2,1}$ (5, 10, 15% permutation sequences), $\tau_{F}$ increases with depth (*B*). Due to the opposing behaviors of $F_{2,1}$ and $\tau_{F}$, $F_{\text{max}}$ does not vary strongly with depth, and there may be an optimal depth where $F_{\text{max}}$ is lowest (*C*). (*D*) Task-relation OPs ($\gamma_{\text{rule}}$ and $\gamma_{\text{RF}}$) on the benchmark sequences). Colors blue, red, and green correspond to permutation sequences, split EMNIST, and split CIFAR100, respectively. Colors from light to dark correspond to increasing depth of the network. In permutation sequences, larger sizes of the points correspond to larger permutation ratio. The benchmark sequences explore a more constrained region in the OP space compared to the target-distractor sequences. (*E*) $F_{2,1}$ is accurately predicted by $2(\gamma_{\text{RF}}-\gamma_{\text{rule}})$, as in Fig. 3*A*. Increasing depth or decreasing the permutation ratio results in smaller $\gamma_{\text{RF}}-\gamma_{\text{rule}}$ (as also shown in *D*), and thus leads to smaller $F_{2,1}$. (*F*) For tasks with small $F_{2,1}$ (5,10,15% permutation sequences), $\tau_{F}$ decreases with $\gamma_{\text{RF}}$, consistent with Fig. 3*D*. For a fixed permutation ratio, increasing depth results in smaller $\gamma_{\text{RF}}$ (as also shown in *D*), and thus leads to larger $\tau_{F}$. All $F_{t,1}$ and the corresponding OPs are averaged across 50 random seeds used for generating data. See *SI Appendix*, section 6 for detailed parameters.

For permutation sequences, we aggregated over different source datasets as forgetting does not differ much between them. For split sequences, forgetting varies more between split EMNIST and split CIFAR-100, so we present their results separately. For all benchmark task sequences we explored, $F_{2,1}$ monotonically decreases with depth (Fig. 4*A*). $F_{2,1}$ is larger for split sequences and smaller for permutation sequences, and increases with a larger permutation ratio. As before, we are primarily interested in long-term forgetting for tasks with small $F_{2,1}$, we thus show $\tau_{F}$ and $F_{\text{max}}$ for permutation sequences with 5 to 15% permutation ratio. As shown in Fig. 4*B*, $\tau_{F}$ increases with depth. Therefore, depth has opposing effects on $F_{2,1}$ and $\tau_{F}$, while deeper networks forget less in the short term, forgetting accumulates over longer periods of time. As a result, the dependence of $F_{\text{max}}$ on depth is more complex. $F_{\text{max}}$ does not change as significantly as $F_{2,1}$, and there may be an optimal depth where $F_{\text{max}}$ is at its lowest (Fig. 4*C*).

### 2.5. Forgetting in Benchmark Sequences are Explained by the OPs.

In this section, we examine whether the dependence of forgetting on depth and across different benchmark sequences can be explained by the OPs as in the target-distractor sequences. To this end, we first computed the OPs for the task sequences, aggregated over the source datasets for the permutation sequences, and separately for split EMNIST and split CIFAR-100 sequences. As we showed in Section 2.2, forgetting does not exhibit significant dependence on $\gamma_{\text{feature}}$, so we focus on $\gamma_{\text{rule}}$ and $\gamma_{\text{RF}}$ in this section.

As shown in Fig. 4*D*, the OPs on the benchmark sequences depend on both the sequence type and the network depth, and altogether partially fill the entire feasible OP space (below the dashed line in Fig. 4*D*). Split sequences including split EMNIST (red points) and split CIFAR-100 (green points) have close to zero $\gamma_{\text{rule}}$, and $\gamma_{\text{RF}}$ decreases with depth (darker colors represent increasing depths). Permutation sequences with large permutation ratios (large blue points) behave similarly to the split sequences. Permutation sequences with small permutation ratios (small blue points) have $\gamma_{\text{RF}}$ close to $\gamma_{\text{rule}}$ across all depths, and for networks with larger depths, $\gamma_{\text{rule}}$ and $\gamma_{\text{RF}}$ decrease simultaneously, such that the tasks become more dissimilar.

To summarize, between-task conflict becomes lower in deeper networks, and in permutation sequences with lower permutation ratio. As verified in Fig. 4*E*, $F_{2,1}$ is still accurately predicted by the conflict. For task sequences with small conflict (permutation sequences with 5 to 15% permutation ratio), both OPs decrease with depth, predicting a larger $\tau_{F}$, as verified in Fig. 4*F*. The relations between the OPs and forgetting in the target-distractor sequences in Section 2.2 also hold for general benchmark task sequences, and can be used to explain the effect of network depth. We also verified that our theory in this regime provides a good qualitative account of gradient descent-trained networks (*SI Appendix*, Fig. S4).

## 3. Networks with Task-Dedicated Readouts

### 3.1. Setup of Multihead CL.

**Fig. 5.**
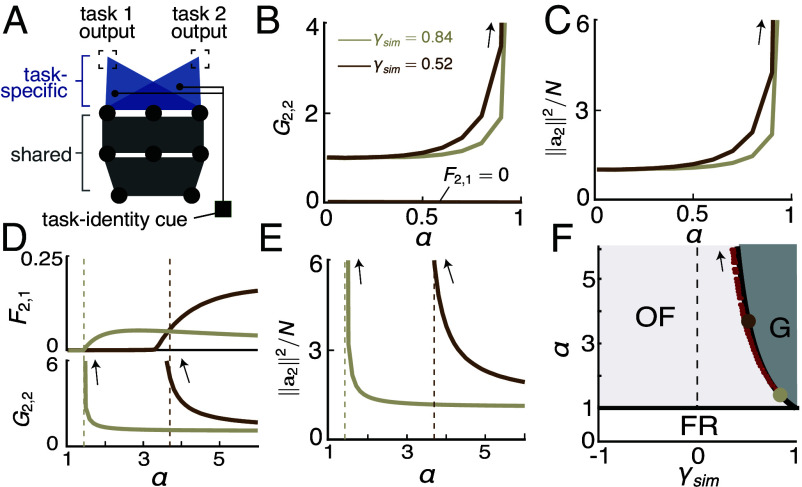
Multihead CL exhibits phase transitions in the target-distractor sequence. (*A*) Schematics of multihead CL. Different tasks utilize the same shared hidden-layer weights but different task-specific readouts. The weight-perturbation penalty is only applied to the hidden-layer weights. (*B*) Forgetting of task 1 ($F_{2,1}$) and the normalized generalization error on task 2 ($G_{2,2}$) as a function of the network load (α) for 2 different sets of $\left(\rho_{\text{target}},\rho_{\text{shared}},\rho_{\text{flip}}\right)$ in the target-distractor task in the fixed-representation regime (FR, α<1). Black arrows indicate divergence toward infinity as α approaches 1. Curves of different colors correspond to tasks with different parameters $\left(\rho_{\text{target}},\rho_{\text{shared}},\rho_{\text{flip}}\right)$. light: $\left(1,0.88,0\right),\gamma_{\text{sim}}=0.84$; dark: $\left(1,0.58,0.005\right),\gamma_{\text{sim}}=0.52$. The generalization errors are calculated on the training data with small perturbations to the input (*SI Appendix*, section 5). (*C*) The norm of $a_{2}$, $\left|\right|a_{2}{\left|\right|}^{2}/N$, as a function of α in the fixed-representation regime (FR). Since the hidden layer representations are fixed, learning the second task is equivalent to learning the linear weights $a_{2}$ in linear regression, thus the divergence of $G_{2,2}$ results from the divergence of $a_{2}$ when approaching the interpolation threshold in linear regression. (*D*) Same as (*B*), but for α>1. For each combination of $\left(\rho_{\text{target}},\rho_{\text{shared}},\rho_{\text{flip}}\right)$, $F_{2,1}$ and $G_{2,2}$ exhibit abrupt changes as α crosses a critical load ($\alpha_{c}$, vertical dashed line). In the overfitting regime (OF, $1<\alpha <\alpha_{c}$), $F_{2,1}$ is zero but $G_{2,2}$ diverges. In the generalization regime (G, $\alpha >\alpha_{c}$), both $F_{2,1}$ and $G_{2,2}$ are moderate and nonzero. (*E*) Same as (*C*), but for α>1. The divergence of $G_{2,2}$ results from the divergence of $a_{2}$ to compensate for minimal $\left|\right|W_{2}-W_{1}\left|\right|$ when learning task 2. (*F*) The transition boundary between the fixed-representation regime (FR) and the overfitting regime (OF) is always at α=1 and does not depend on the task. The transition boundary between the overfitting regime (OF) and the generalization regime (G), $\alpha_{c}$, can be theoretically predicted by the task similarity metric $\gamma_{\text{sim}}\in \left[-1,1\right]$ under reasonable assumptions (*SI Appendix*, section 4.*A*), as shown by the black line. Each red point shows the estimated transition boundary $\alpha_{c}$ from the shape of $F_{2,1}$ (*SI Appendix*, section 5) for a different combination of $\left(\rho_{\text{target}},\rho_{\text{shared}},\rho_{\text{flip}}\right)$, and thus a different value of $\gamma_{\text{sim}}$. The red points lie on top of the black curve, demonstrating the accuracy of the theoretical prediction. The light and dark brown points correspond to the lines shown in (*B* and *C*).

In many CL settings, both in ML applications and naturalistic environments for animals, the learner is aware (through external cues or inference) of the identity of the current task being learned or performed. A simple method of incorporating such information into the network (28, 29, 41) is to use task-specific readouts (“multihead” CL). Learning a new task involves modifying the shared hidden-layer weights while adding a new task-specific readout, leaving previous readouts untouched (Fig. 5*A*). The network has t different input–output mappings after learning t tasks, given by

[10] $$\begin{array}{c} f_{t}^{\tau }\left(x\right)\equiv \frac{1}{\sqrt{N}}a_{\tau }\cdot \Phi \left(W_{t},x\right)\;\tau =1,\cdots ,t. \end{array}$$

At time t, the network selects the mapping $f_{t}^{\tau }\left(x\right)$ to perform the τ-th task. Since the readout weights ${\left\{a_{\tau }\right\}}_{\tau =1,\cdots ,t}$ are task-dedicated and the hidden-layer weights W are shared, only the changes in W need to be constrained in order to mitigate forgetting. Due to these differences from single-head CL, the objective function of learning is given by Eq. **2** but with $f_{t}\left(x\right)$ replaced with $f_{t}^{t}\left(x\right)$ and the regularization term $\left|\right|\Theta_{t}-\Theta_{t-1}{\left|\right|}^{2}$ replaced with $\left|\right|W_{t}-W_{t-1}{\left|\right|}^{2}$.

The presence of task-specific parameters generally makes forgetting less severe than that in single-head CL (42). Importantly, this architecture allows the network to perform tasks with high conflict, which single-head networks struggle with, as shown in the previous section. In fact, in the infinite-width limit (N→∞,α→0) studied above, forgetting and anterograde effects can be entirely avoided regardless of task relations, since the network can simply freeze its random hidden-layer weights and learn a separate readout for each task. However, this simple scheme breaks down in the more realistic cases where resources are limited and the network may have to modify the hidden-layer weights to solve each task.

To study interesting properties of CL in the task-dedicated multihead architecture, we focus on the thermodynamic limit, defined by P,N→∞ and α≡P/N∼O(1). We focused on the case of T=2 and L=1 (due to the complexity of the theory in this limit, but see *SI Appendix*, Figs. S7 and S8 for results beyond these restrictions). Our theory analytically evaluates forgetting of task 1 and the anterograde effect on task 2 in multihead CL, respectively given by $F_{2,1}=\langle L\left(f_{2}^{1},D_{1}\right)\rangle$ and $G_{2,2}=\langle L\left(f_{2}^{2},D_{2}^{\mathrm{test}}\right)\rangle /G_{2}^{0}$, where $G_{2}^{0}$ is the generalization error on task 2 when learning it alone.

### 3.2. Phase Transitions in CL Performance in the Target-Distractor Sequence.

We first used the target-distractor task sequences to probe how task relations affect CL performance in the limit of λ→∞. In addition to varying $\left(\rho_{\text{shared}},\rho_{\text{target}},\rho_{\text{feature}}\right)$ as in the single-head analysis, we also varied the load α≡P/N. In Fig. 5*B*–*E*, we show two examples of task sequences generated under two combinations of $\left(\rho_{\text{shared}},\rho_{\text{target}},\rho_{\text{feature}}\right)$, and plot $F_{2,1}$ and $G_{2,2}$ as α increases. We found that, regardless of task relations, $F_{2,1}$ is zero as long as α<1, while $G_{2,2}$ diverges to infinity as α approaches 1 (Fig. 5*B*). Such behaviors can be explained by the fact that when α<1, learning the task-2 readout ($a_{2}$) alone is sufficient to interpolate $D_{2}$, requiring no change to the hidden-layer weights (W). Due to the strong perturbation penalty (λ→∞), W do not change, maintaining the network representations after learning task 1 (as derived in *SI Appendix*, section 4.B.6). This can also explain the divergence of $G_{2,2}$ as $\alpha \to 1^{-}$: learning $D_{2}$ by modifying $a_{2}$ on top of the N-dimensional fixed representations is effectively a linear regression, the generalization error of which is well known to diverge as P approaches N (43). This divergence is due to the divergence of the norm of $a_{2}$ (Fig. 5*C*). For smaller α, the zero $F_{2,1}$ and $G_{2,2}$ remaining close to 1 demonstrate the advantage of using task-specific readouts. We term this regime of α<1, where $F_{2,1}=0$ and $G_{2,2}$ is mostly finite, the “fixed representations” regime (FR).

As α increases past 1, interpolating $D_{2}$ requires changing W. Consequently, we expected that such changes would induce forgetting of task 1. Surprisingly, we found that there exists a critical load, $\alpha_{c}>1$, under which forgetting remains zero (Fig. 5*D*, *Top*). Further analysis showed that while changes in the network representations after learning task 1 no longer have zero norm, it is confined within the null space of $a_{1}$ and thus does not alter the output on task 1 (*SI Appendix*, section 4.B.6). Although the absence of forgetting is desirable, this regime is accompanied by the network’s inability to generalize on the second task, despite reaching zero training error. In fact, $G_{2,2}$ diverges (Fig. 5*D*, *Bottom*), indicating the surprising phenomenon we term “catastrophic anterograde interference,” where previous learning completely impedes generalization of new learning. We term this regime, where $F_{2,1}=0$, and $G_{2,2}\to \infty$, the “overfitting” regime. The divergence of $G_{2,2}$ in this regime is also due to the divergence of $a_{2}$ (Fig. 5*E*). In this regime, the minimal changes in the hidden layer weights W result in a hidden representation that does not learn the task rule of the second task (as we show later in Section 3.4), causing $a_{2}$ to diverge in order to compensate. As α further increases past $\alpha_{c}$, the network abruptly enters the “generalization” regime where $F_{2,1}>0$ and $G_{2,2}$ becomes finite. In this regime, the changes in the representation after learning task 1 are no longer confined to the null space of $a_{1}$, inducing forgetting. The network partially forgets task 1, but learns to generalize on task 2.

Importantly, the boundary separating the two regimes ($\alpha_{c}$) depends on task relations. Different combinations of $\left(\rho_{\text{target}},\rho_{\text{shared}},\rho_{\text{flip}}\right)$ are associated with a different $\alpha_{c^{\prime }s}$, separating the overfitting and generalization regimes. We found that under reasonable approximations, $\alpha_{c}$ is fully determined by a new OP measuring overall similarity of the two tasks, defined as

[11] $$\begin{array}{c} \gamma_{\text{sim}}=\gamma_{\text{feature}}+\cos\left(V_{1},V_{2}\right)-V_{1}^{⊤}P_{2}V_{1}/\left|\right|V_{1}{\left|\right|}^{2}, \end{array}$$

where $P_{i}$ and $V_{i}$ (i≤2) are defined as in Eqs. **8** and **9**. Although the precise definitions of the three terms in $\gamma_{\text{sim}}$ are different from the 3 OPs for single-head CL (introduced in Section 2.1) since we now focus on a different limit in a different CL architecture, they are actually closely related. The first term in $\gamma_{\text{sim}}$, $\gamma_{\text{feature}}$, is exactly the same as defined in Section 2.1. The second term, $\cos(V_{1},V_{2})$, measures the cosine similarity between the rule vectors of the two tasks; it has a similar interpretation as $\gamma_{\text{rule}}$. The third term, $V_{1}^{⊤}P_{2}V_{1}/\left|\right|V_{1}{\left|\right|}^{2}$, measures the projection of the rule vector onto the shared input feature subspace of the two tasks; it has a similar interpretation as $\gamma_{\text{RF}}$. $\gamma_{\text{sim}}$ is within the range of [−1,1]. For conflicting tasks with the same input feature subspaces but opposite rule vectors, $\gamma_{\text{sim}}=-1$, for identical tasks $\gamma_{\text{sim}}=1$. For dissimilar tasks with small overlap between input feature subspaces (small $\gamma_{\text{feature}}$), and rule vectors lying in the nonoverlapping input feature subspaces of each task (small second and third terms in Eq. **11**), $\gamma_{\text{sim}}$ is close to 0. As shown in Fig. 5*F*, for α<1, the network is in the “fixed representation” regime, independent of task relations. For α>1, the phase transition $\alpha_{c}$ is accurately predicted by $\alpha_{c}=\gamma_{\text{sim}}^{-2}$ (black line) across different parameters of the target-distractor tasks (red points). $\alpha_{c}$ is larger for more dissimilar tasks with smaller $\gamma_{\text{sim}}$, resulting in a smaller generalization regime. For $\gamma_{\text{sim}}<0$, $\alpha_{c}=\infty$, and the network is always in the overfitting regime as long as α>1.

### 3.3. Phase Transitions in Benchmark Sequences.

**Fig. 6.**
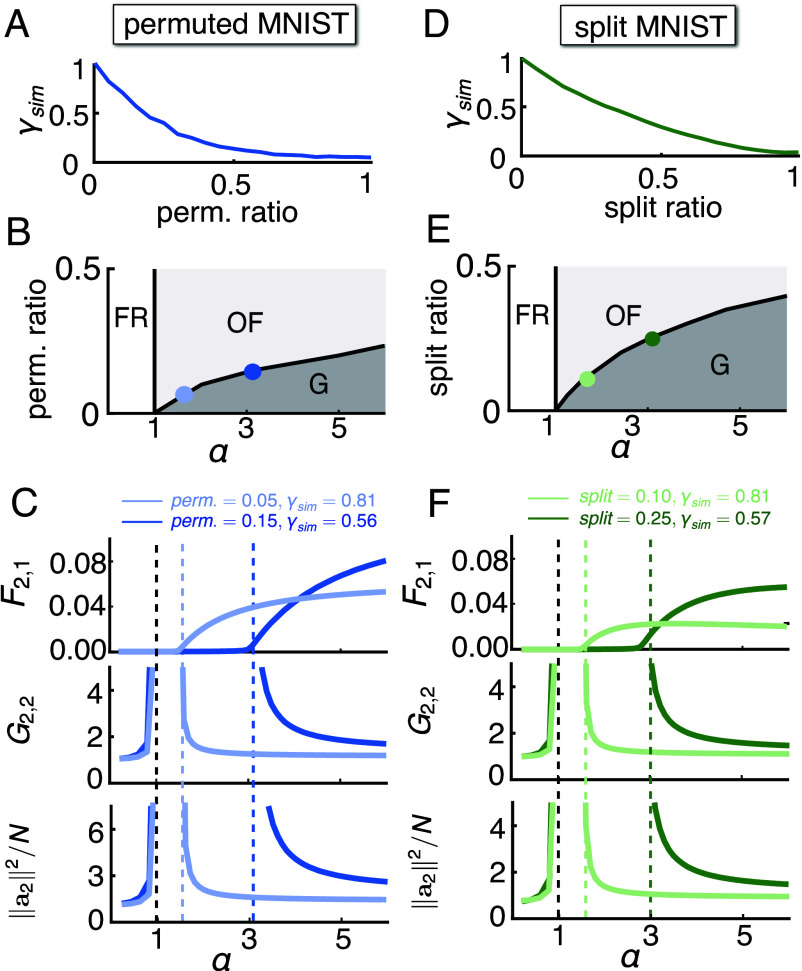
Similarity OP predicts phase transition in benchmark sequences. (*A*) For permuted MNIST with different permutation ratios, we can calculate the task similarity metric $\gamma_{\text{sim}}$, $\gamma_{\text{sim}}$ decreases with increasing permutation ratio. (*B*) Using $\gamma_{\text{sim}}$ shown in (*A*), the theory predicts a phase diagram in the permutation ratio-α space, showing the three regimes as in the target-distractor sequences: fixed representations (“FR”), overfitting (“OF”), and generalization (“G”). (*C*) $F_{2,1}$, $G_{2,2}$ and $\left|\right|a_{2}{\left|\right|}^{2}/N$ corresponding to the permutation ratios of the two points in (*B*) (light blue: perm. =0.05, $\gamma_{\text{sim}}=0.81$; dark blue: perm. =0.15, $\gamma_{\text{sim}}=0.56$), the transition from zero to positive $F_{2,1}$ and from diverging to finite $G_{2,2}$ or $\left|\right|a_{2}{\left|\right|}^{2}/N$ is accurately predicted by the theoretical $\alpha_{c}$ (light blue dashed line: $\alpha_{c}=1.51$, dark blue dashed line: $\alpha_{c}=3.19$). (*C*) $F_{2,1}$, $G_{2,2}$ and $\left|\right|a_{2}{\left|\right|}^{2}/N$ corresponding to the permutation ratios of the two points in (*B*) (light blue: perm. = 0.05, $\gamma_{\text{sim}}=0.81$; dark blue: perm. =0.15, $\gamma_{\text{sim}}=0.56$), the transition from zero to positive $F_{2,1}$ and from diverging to finite $G_{2,2}$ or $\left|\right|a_{2}{\left|\right|}^{2}/N$ is accurately predicted by the theoretical $\alpha_{c}$ (light blue dashed line: $\alpha_{c}=1.51$, dark blue dashed line: $\alpha_{c}=3.19$). (*D*–*F*) Same as (*A*–*C*), but for split MNIST, where we control the task similarity by changing the split ratio (*SI Appendix*, section 6). $\gamma_{\text{sim}}$ decreases with increasing split ratio. The two examples shown in (*E* and *F*) correspond to split ratio 0.1 (light green, $\gamma_{\text{sim}}=0.81$) and 0.25 (dark green, $\gamma_{\text{sim}}=0.57$), and the theoretical prediction of their $\alpha_{c}$’s are 1.54 and 3.03 respectively.

Our results suggest that the three phases are in fact general phenomena, and the OP $\gamma_{\text{sim}}$ can be used to predict the phase transition boundary $\alpha_{c}$ across different task sequences. To verify, we computed the OP $\gamma_{\text{sim}}$ for two types of benchmark task sequences, permuted and split MNIST, where the task similarity is controlled by varying the permutation or split ratio (*SI Appendix*, section 6). Smaller permutation or split ratios correspond to intuitively more similar tasks and vice versa. As shown in Fig. 6 *A* and *D*, $\gamma_{\text{sim}}$ decreases with the permutation ratio or the split ratio, capturing the changes in the task similarity. When the permutation (split) ratio is 0, the tasks are identical and $\gamma_{\text{sim}}=1$, whereas when the permutation (split) ratio is 1, the tasks are dissimilar and $\gamma_{\text{sim}}\approx 0$.

Using $\gamma_{\text{sim}}$, our theory predicts an $\alpha_{c}$ for each permutation (split) ratio, producing a phase diagram in the permutation (split) ratio-α space (Fig. 6 *B* and *E*), with the same three regimes as we showed in the target-distractor sequences: the fixed representations regime (FR), the overfitting regime (OF), and the generalization regime (G). To verify the prediction of the phase diagram, we selected two examples for each type of task sequences with different permutation (split) ratios, and thus different $\gamma_{\text{sim}}$, and computed $F_{2,1}$, $G_{2,2}$ and $\left|\right|a_{2}{\left|\right|}^{2}/N$ as a function of α. As shown in Fig. 6 *C* and *F*, the theoretical prediction of $\alpha_{c}$ (dashed lines) accurately captures the abrupt changes in the performance of the network: For α<1, $F_{2,1}=0$, $G_{2,2}$ and $\left|\right|a_{2}{\left|\right|}^{2}/N$ are finite and start to diverge as $\alpha \to 1^{-}$; for $1<\alpha <\alpha_{c}$, $F_{2,1}$ remains 0, $G_{2,2}$ and $\left|\right|a_{2}{\left|\right|}^{2}/N$ are diverging; for $\alpha >\alpha_{c}$, $F_{2,1}$, $G_{2,2}$ and $\left|\right|a_{2}{\left|\right|}^{2}/N$ are all finite and nonzero. Finally, these qualitative behaviors of phase transitions were reproduced in gradient-descent trained networks (*SI Appendix*, Fig. S7) as well as in CL of longer task sequences (*SI Appendix*, Fig. S8).

### 3.4. Balancing Memorization and New Learning with Finite λ.

**Fig. 7.**
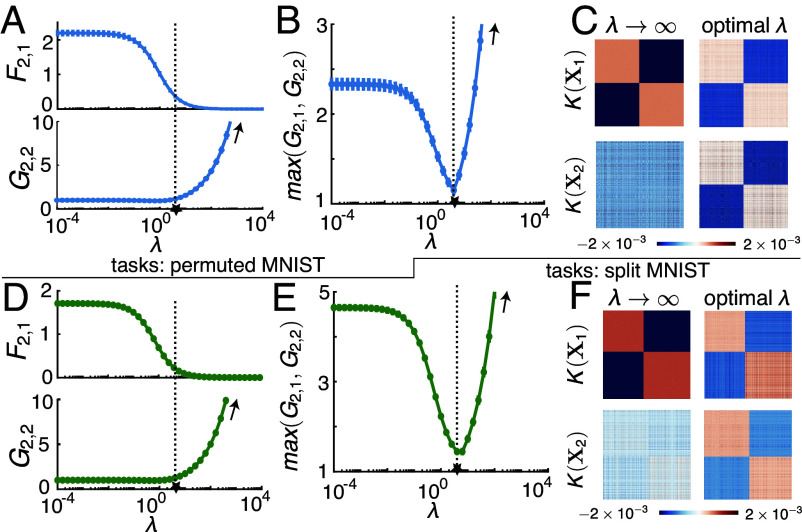
Optimal regularization strength balances memorization and learning new tasks. (*A*) Forgetting of task 1 ($F_{2,1}$) monotonically decreases with the regularization strength (λ) while the normalized generalization error on task 2 ($G_{2,2}$) monotonically increases. The two tasks considered here are sufficiently dissimilar (permuted MNIST with 100% permutation ratio) that they are in the overfitting regime and $G_{2,2}$ diverges at large λ. (*B*) Maximum of the normalized generalization error on task 1 and task 2 ($\max(G_{2,1},G_{2,2})$) as a function of the regularization strength λ. There exists an intermediate optimal λ which minimizes $\max(G_{2,1},G_{2,2})$ by keeping both of them close to 1 such that there is minimal interference coming from the other task, indicated by the star. (*C*) The learned component of the similarity matrix of the hidden layer activations on task 1 and task 2 training data ($X_{1}$ and $X_{2}$), after learning the two tasks, denoted $K(X_{1})$ (*Top* row) and $K(X_{2})$ (*Bottom* row) respectively. At λ→∞, only $K(X_{1})$ but not $K(X_{2})$ exhibits a task-relevant block structure. This indicates that the network fails to learn good representations for task 2, and overmemorizes task 1. In contrast, at the optimal λ (corresponding to the star in *B*), both $K(X_{1})$ and $K(X_{2})$ show a block structure aligned with their corresponding tasks, exhibiting a shared representation beneficial for both tasks. (*D*–*F*) Same as (*A*–*C*), but for split MNIST with 100% split. In (*A*, *B*, *D*, and *E*), error bars are SDs across 10 different random seeds of task sequence generation. α=3 in both examples, which is below the corresponding $\alpha_{c}$ for these sequences.

The analysis so far has shown that, when tasks are sufficiently dissimilar, λ→∞ can cause the network to memorize perfectly (zero $F_{2,1}$) at the expense of catastrophic anterograde interference (diverging $G_{2,2}$). We next characterized the trade-off for such dissimilar tasks between improving $G_{2,2}$ and maintaining low $F_{2,1}$ by lowering λ. As expected, as λ lowers, the network forgets the first task more (higher $F_{2,1}$, Fig. 7 *A* and *D*, *Top*), resulting in weaker interference of the second task (lower $G_{2,2}$, Fig. 7 *A* and *D*, *Bottom*). To evaluate and compare the performance on both tasks and quantify the trade-off, we also computed the normalized test loss on task 1 after learning task 2, denoted by $G_{2,1}$, and studied $\max(G_{2,1},G_{2,2})$ as a function of λ (Fig. 7 *B* and *E*). We found that there exists a finite optimal λ that minimizes $\max(G_{2,1},G_{2,2})$ by keeping both $G_{2,1}$ and $G_{2,2}$ close to 1.

We next sought to understand how the representations of task 1 and task 2 inputs depend on λ by studying the representation similarity matrix after learning both tasks. Specifically, we analyzed the learned component in the representation similarity matrix on the training data $X_{1}$ and $X_{2}$ (*SI Appendix*, sections 4.B and 6), denoted by $K\left(X_{1}\right)\in R^{P\times P}$ and $K\left(X_{2}\right)\in R^{P\times P}$ respectively. Prior work has indicated that, for binary classification tasks that we considered, a 2×2 block structure in the similarity matrix suggests that the representations are clustered according to the task labels and is associated with good generalization performance (44–47). Indeed, we found that at large λ, the similarity matrix has such 2×2 block structure for $X_{1}$ but not $X_{2}$ (Fig. 7 *C* and *F*), explaining our previous finding that the network fails to generalize on task 2 in the overfitting regime. However, when using the optimal λ that minimizes $\max\left(G_{2,1},G_{2,2}\right)$, representations of inputs from both tasks have such block structure, consistent with the finding that both $G_{2,1}$ and $G_{2,2}$ are close to 1, highlighting the importance of representation learning on the generalization capabilities in CL.

## 4. Discussion

### 4.1. Related Works.

Many mechanisms have been proposed for mitigating catastrophic forgetting (for a recent review, see ref. 48). We used the simplest approach by adding an $L_{2}$ penalty on weight changes to facilitate theoretical analysis. While this work’s aim is not to achieve state-of-the-art performance but to develop a theoretical understanding of CL, it is important that our theoretical results achieve reasonable performance compared to commonly used CL approaches. We confirmed this by comparing against networks trained using gradient descent with online EWC (49) or L2 regularizers (*SI Appendix*, section 8). Theoretical advances on CL have been made recently. For single-head CL, our theoretical results (*SI Appendix*, Eqs. **S16** and **S17**) are consistent with refs. 18 and 24 in the λ→∞ limit. However, we stress that our theoretical framework and assumptions are different. While (18, 24) assume linearization of the dynamics around initialization in the NTK regime (19), our formulation does not rely on any assumptions on the learning dynamics. Interestingly, we recover an NTK-like theory of CL in the single-head scenario when λ→∞. Furthermore, while these previous results proposed an analytical expression for the mean predictor, they did not provide explicit predictions of the theory on CL performance. In this work, combining our theoretical solutions and numerical evaluations, we characterized the connections between forgetting in CL and task relations using the theoretically inspired OPs. We qualitatively verified qualitative predictions in gradient descent-trained networks (*SI Appendix*, Fig. S4). Importantly, our Gibbs formulation also allows us to investigate the effect of λ (*SI Appendix*, Fig. S3), which is not amenable in the NTK formulation in refs. 18 and 24. For multihead CL, most theoretical works make specific assumptions about the tasks (20–22), limiting their applicability. We became aware of a very recent work during the preparation of our manuscript (50), which adopts a Gibbs formulation for transfer learning similar to our multihead setup and does not make specific assumptions about the statistics of the tasks. However, this work focused on learning in the regime α<1 with finite λ, and therefore did not uncover the intriguing phenomenon of phase transitions that are determined by task similarities as in our work. It also does not identify the order parameters governing these transitions and predicting other aspects of CL performance.

### 4.2. Forgetting and Task Relation OPs.

We systematically investigated how task relations influence forgetting in $L_{2}$-regularized CL in wide DNNs in single-head and multihead scenarios, studying short-term forgetting (sequential learning of two tasks) and, in the case of single-head CL, long-term forgetting (a long sequence of tasks). In contrast to prior work, which mostly treated “task similarity” as a single variable (2, 16, 18, 21, 22, 51, but see refs. 13 and 20), our analysis emphasizes the importance of distinguishing different aspects of task relations on CL. Importantly, we identified several scalar OPs quantifying these task relations. These OPs can be evaluated given the training data of each task and are highly predictive of CL performance in the settings that we studied. We summarize the definitions of these OPs in Table 1.

For single-head CL, we studied $\gamma_{\text{feature}}$, $\gamma_{\text{RF}}$ and $\gamma_{\text{rule}}$. Interestingly, $\gamma_{\text{feature}}$ is only weakly related to forgetting, while the other two OPs play important roles. This suggests that the similarity between the input features, which are relevant for the task, rather than the input features themselves, are the determining factors of forgetting. $\gamma_{\text{RF}}-\gamma_{\text{rule}}$, which we termed the conflict between tasks, is directly related to short-term forgetting. For tasks with low conflict and thus small short-term forgetting, long-term forgetting accumulates slowly for dissimilar tasks (small $\gamma_{\text{RF}},\gamma_{\text{rule}}$) and quickly for similar tasks (high $\gamma_{\text{RF}},\gamma_{\text{rule}}$). That lowering $\gamma_{\mathrm{RF}}$ reduces forgetting is consistent with CL methods that explicitly learn representations of inputs from different tasks in mutually orthogonal subspaces (52, 53). A potential promising direction is to design methods that encourage networks to learn “reusable” features and increase $\gamma_{\mathrm{rule}}$. For multihead CL, our theory identifies another task similarity OP $\gamma_{\text{sim}}$ composed of 3 terms, each bearing resemblance to the 3 OPs in single-head CL including $\gamma_{\text{feature}}$, which does not significantly affect single-head CL performance. The effect of this OP depends also on the load α. For α<1, task relations have no effect on forgetting as it vanishes at λ→∞. However, for α>1 there exists a $\gamma_{\text{sim}}-\alpha$ phase diagram (Fig. 5*D*). For a fixed load (α), when $\gamma_{\text{sim}}$ are high, CL is in the generalization regime where forgetting is nonzero but moderate. When the tasks become sufficiently dissimilar, CL abruptly enters the overfitting regime where forgetting is zero but generalization on the new task fails despite reaching zero training error, a surprising phenomenon we termed “catastrophic anterograde interference.” For tasks in this regime, fine-tuning λ of the learner can reach a reasonable compromise and allow the network to perform both tasks (Fig. 7).

### 4.3. Architecture.

Our analysis suggests that task relations are modulated by the architecture of the learner. Increasing depth effectively mitigates single-head forgetting for short task sequences (decreased $F_{2,1}$, Fig. 4*A*) by reducing the conflict between tasks but has a more complicated effect on long-term forgetting (reflected in nonmonotonic $F_{\text{max}}$, Fig. 4*C*) due to the opposing effects it has on $F_{2,1}$ and $\tau_{F}$. In addition, increasing the width (N), which we studied for multihead CL, can also mitigate forgetting. As N increases for a fixed dataset size (P), α decreases below $\alpha_{c}$. As a result, CL transitions from the generalization regime, where forgetting is finite, to the overfitting regime, where it is zero. Although the specific value of $\alpha_{c}$ depends on task relations, our theory indicates that the transition to zero forgetting is a general phenomenon. Widening the network further eventually causes α to drop below 1 where network features are fixed and forgetting is zero for any tasks. The observed beneficial effects of depth and width on mitigating forgetting are consistent with empirical reports of less forgetting in larger networks (54).

### 4.4. Anterograde Effects.

In addition to forgetting (retrograde interference), we investigated anterograde aspects of CL by studying how learning one task affects the generalization performance on a subsequently learned one. For single-head CL, we omitted discussion of anterograde effects from the main text as they are generally weak in the infinite width limit that we consider (*SI Appendix*, Fig. S2), consistent with previous reports. However, results from multihead (Figs. 5–7) CL indicate that anterograde interference can be severe and worsens as the tasks become less similar. This suggests a parameter regime at α>1 where, counterintuitively, single-head CL performs better than multihead (*SI Appendix*, Fig. S9). It would be interesting to rigorously verify this in a future theory of single-head CL with finite α. The existence of diverging test loss for α>1 suggests that increasing the width of the network (reducing α to a value below 1) will have a very beneficial effect on sequential learning. While anterograde interference appears prevalent and severe in our analysis, this is partially due to the specific settings we focused on. Assuming the second task to have substantially fewer training examples than the first or a compositional structure between tasks (55) could lead to stronger positive transfer effects. In addition, making transitions between dissimilar tasks “smoother” by inserting intermediate datasets can mitigate anterograde interference in multihead CL (based on a generalization of the theory to T=3; *SI Appendix*, Fig. S8).

### 4.5. Implications for CL in the Brain.

Recent neuroscience experiments indicate that neural representations of a learned task can “drift” after learning has concluded (12, 56), raising the question of how the brain maintains stable task performance despite such drifts (57). While a multitude of mechanisms likely underlie this phenomenon, subsequent learning of other tasks by the same neural circuits likely contributes (12). As shown by our analysis, this can indeed occur during multihead CL at α>1, where representations of task 1 inputs are altered by learning the second task. Our analysis hints at how the brain may deal with this issue. Task 1 performance can be unperturbed as long as representational changes occur only in the null space of its readout, consistent with the notion that the brain orthogonalizes representations for different tasks to reduce interference (12, 58, 59). The overfitting regime demonstrates that such orthogonality can occur without storing task 1 inputs and explicitly confining new learning in their null space, as long as the penalty on weight perturbations is sufficiently strong. To avoid the failure to generalize on task 2 in this regime, the brain may weaken the penalty or enforce a hard constraint on the strength of the readout weights, such that representational changes are still mostly orthogonal to the task 1 readout but sufficient for good generalization of task 2. These results suggest the possibility of enforcing near-orthogonality between task subspaces by having a regularization-like mechanism [e.g., synaptic stabilization (10, 11)] along with appropriately tuned penalty or constrained readout weight strength.

Our results also highlight how architectural elements of the brain can confer CL benefits. Sensory expansion, a motif often seen in sensory cortices, projects a low-dimensional input signal into a much higher-dimensional code within a large population of neurons (60). From the perspective of multihead CL, this may effectively increase the NN width and reduce forgetting, as discussed above. Additionally, our finding that increasing depth can mitigate forgetting may indicate an advantage of having a deep, multistage sensory processing system. This suggestion predicts that representations of different tasks are less similar in later stages of sensory processing (61, 62). To assess such similarity in high-dimensional neural codes without resorting to nonlinear dimensionality-reduction techniques (e.g., 59), it may be promising to adapt our OPs to experimental data.

Finally, it would be interesting to test whether the same connections between task relations and severity of forgetting hold in the brain. For instance, animals can be sequentially trained on a series of two-alternative forced choice tasks. In each task, the animal would need to distinguish two classes of simple stimuli with a few attributes, much like the example shown in Fig. 1*A*. Different tasks would contain different dichotomies on different attributes. The dichotomies should be consistent across tasks to ensure a small conflict $\gamma_{\text{RF}}-\gamma_{\text{rule}}$. Assuming animals are using a single-head-like shared behavioral readout for these tasks (63) and a regularization-like mechanism for CL, our results predict forgetting will accumulate longer if attributes from different tasks are made more distinct (lower $\gamma_{\text{RF}}$ and $\gamma_{\text{rule}}$).

### 4.6. Extensions and Limitations.

The presented theory can be extended in several important directions. First, our Gibbs formulation assumes a uniform perturbation penalty across all weights, while popular regularization-based CL methods typically use some metric to evaluate the importance of each individual weight for past performance and apply a stronger penalty to more important ones (6, 31). Our theory may be extended to the case with weight-specific penalties and elaborate on how different importance metrics affect CL outcomes.

Second, we assumed that the tasks are symmetric and have similar pairwise task relations in long task sequences, which prevents us from capturing how different orderings of the same set of tasks elicit different CL performance. While we have neglected ordering effects here because they are often small in common task sequences (16, 64), it remains an interesting future direction to systematically probe into the effect of task ordering in more structured progressive task sequences. Another important direction for future research is the study of heterogeneous task sequences, such as curriculum learning ones where the task difficulty progressively increases (65) While our analytical theory is also applicable to such cases, it remains to be seen what OPs can predict CL phenomena there.

Finally, while we have focused on leveraging task-identity information during CL using the multihead scheme, having multiple readouts is considered less realistic for CL in the brain. Conceptually, the task identity information can be incorporated through gating units that gate parts of the readout weights on or off depending on the task, as in ref. 66, making the multihead scheme more biologically relevant. Task-identity information can be incorporated into single-head CL by appending a task-identity embedding vector to relevant inputs (67) or gating individual neurons in a task-dependent manner (68–70). In *SI Appendix*, Fig. S6, preliminary results show that while appending task-identity embedding vectors to the inputs helps mitigate forgetting, its beneficial effect is still weaker compared to adding task-dependent readouts (multihead CL). Extending our theory to other mechanisms of incorporating task-identity information such as gating and studying how they affect the OPs and CL performance is a promising future research direction.

matseccnt1

## 5. Methods

Additional results and further details are provided in *SI Appendix*. *SI Appendix*, section 1 precisely defines the feedforward network architecture under consideration. *SI Appendix*, section 2 derives and presents the generalized kernel functions, which extend the results of ref. 71 and are important building blocks for the theories. *SI Appendix*, section 3 presents full details of the single-head theory, where the network uses the same readout for all tasks. It includes results and discussions of the theory away from the λ→∞ limit discussed here. Analytical forms of generalized kernel functions for linear and ReLU networks are provided and the connection to NTK is specified. *SI Appendix*, section 4 presents the multihead theory, where the network uses a different readout for each task. Generalization of the kernel renormalization technique (41) to continual learning is discussed in detail. A detailed study of how hidden-layer representations change is provided. *SI Appendix*, sections 5 through 8 provide details on the numerical analyses: implementation details of the target-distractor tasks (*SI Appendix*, section 5) and benchmark tasks (*SI Appendix*, section 6), details on using exponential fitting to describe long-term forgetting (*SI Appendix*, section 7) and gradient descent simulations (*SI Appendix*, section 8). *SI Appendix*, sections 9 through 11 provide additional numerical results that support or supplement conclusions in the main text.

## Supplementary Material

Appendix 01 (PDF)

## Data Availability

Code and scripts data have been deposited in https://github.com/hzshan/gp_continual_learning. Previously published data were used for this work (36, 38–40).
