# Supplementary material for: Order parameters and phase transitions of continual learning in deep neural networks
Source: Proc Natl Acad Sci U S A. 2026 Feb 6;123(6):e2501899123. doi: 10.1073/pnas.2501899123 (PMC12890896; doi:10.1073/pnas.2501899123)
Supplement: Supplementary file 1 — Appendix 01 (PDF) [file pnas.2501899123.sapp.pdf]

PNAS

Supporting Information for

Order parameters and phase transitions of continual learning in deep neural networks

Haozhe Shan, Qianyi Li and Haim Sompolinsky

Haim Sompolinsky.
E-mail: hsompolinsky@mcb.harvard.edu

This PDF file includes:

Supporting text
Figs. S1 to S9
SI References

Contents

I Theoretical Results 3

1 Network Architecture 3

2 Generalized Kernel Functions 3
A Kernel Functions for Arbitrary λ . . . 3
B Kernel Functions in the $\lambda \to \infty$ Limit: . . . 4

3 Single-Head Theory 4
A Summary of Main Theoretical Results . . . 4
A.1 Statistics of the Input-Output Mappings . . . 4
A.2 The Order Parameters . . . 5
B Detailed Derivations . . . 5
B.1 Moment Generating Function . . . 5
B.2 Definition of Kernel Functions . . . 7
B.3 Derivation of the Statistics of Input-Output Mappings . . . 7
B.4 Analytical Forms of Kernel Functions in Linear and ReLU Neurons . . . 9
B.5 $\tilde{K}^L_{t,t'}$ and the Neural Tangent Kernel . . . 10

4 Multi-Head Theory 11
A Summary of Main Theoretical Results . . . 11
A.1 The Mean Input-Output Mappings . . . 11
A.2 The Variance of the Input-Output Mappings . . . 12
B Detailed Derivation . . . 12
B.1 Kernel Renormalization Theory . . . 12
B.2 Statistics of the Input-Output Mappings for Arbitrary Number of Tasks T . . . 15
B.3 Self-Consistent Equations for the Renormalization Factors Up to $T = 3$ . . . 16
B.4 Interpretation of Renormalization Factors . . . 18
B.5 Predicting Phase Transition Boundaries with the Task Simialrity OP . . . 19
B.6 Hidden-Layer Kernels and Representational Changes . . . 20

II Details and Parameters of Numerical Experiments 21

5 The Target-Distractor Task Sequence 21

6 Benchmark Task Sequences 21
A Permutation . . . 22
B Split . . . 22

7 Exponential Fitting of Long-Term Forgetting 22

8 Gradient Descent Simulations 22
A Single-Head CL . . . 22
A.1 Gradient Descent . . . 22
A.2 L-2 regularizer . . . 22
A.3 Online EWC . . . 22
B Multi-Head CL . . . 23

III Additional numerical results 23

9 Numerical results on single-head CL 23

10 Numerical results on multi-head CL 27

11 Numerical comparison between single-head and multi-head CL 29

Supporting Information Text

Part I

Theoretical Results

In this section, we will present a summary and derivations of our theoretical results. We begin by introducing more formally the network architecture (SI 1) and defining the important kernel functions which will show up repeatedly in our theoretical results (SI 2). We then present single-head CL and multi-head CL theories separately, first summarizing the main theoretical results (SI 3.A and SI 4.A), and then presenting the detailed derivations (SI 3.B and SI 4.B).

1. Network Architecture

All networks we studied have a fully-connected feedforward body. $\boldsymbol{x}^{l=0,\ldots,L}$ denote the vector of activation in the l-th hidden layer in response to input $\boldsymbol{x}$ ($\boldsymbol{x}^0 \equiv \boldsymbol{x}$), given by

$$x_i^{l+1}(\boldsymbol{x}) = \phi\left(\frac{1}{\sqrt{\dim(\boldsymbol{x}^l)}}\sum_j W_{ij}^l x_j^l(\boldsymbol{x})\right), \qquad [1]$$

where $\dim\left(\boldsymbol{x}^0\right) = N_0$ and $\dim\left(\boldsymbol{x}^{1,\ldots,L}\right) = N$. The representation of an input $\boldsymbol{x}$ denotes the last-layer activation, $\Phi(\mathcal{W}, \boldsymbol{x}) \equiv \boldsymbol{x}^L(\boldsymbol{x})$. $\phi : \mathbb{R} \to \mathbb{R}$ is the single-neuron activation function, taken to be ReLU ($\phi(x) = \max\{0, x\}$).

2. Generalized Kernel Functions

Our theoretical results showed that the statistics of the network's input-output mappings depend on the input data through several generalized kernel functions, similar in spirit to the Neural Tangent Kernel (NTK) and Neural Network Gaussian Process (NNGP) theories of learning (1, 2). We will introduce these kernel functions and the notations we will use throughout in this section, and the main theoretical results in SI 3 and SI 4.

There are two crucial differences between the generalized kernel functions in our theory and the kernels in NTK/NNGP theories. First, our kernel functions are "generalized" in that they are generally asymmetric with respect to the inputs and thus are not proper kernels (3). Second, our kernel functions are time-dependent, as opposed to the stationary NTK/NNGP kernels in classic results. For brevity, we simply refer to the generalized kernel functions as kernels or kernel functions hereafter.

The kernel functions are defined as the inner products between random features averaged over correlated Gaussian weights. The statistics of the Gaussian weights are given by the prior contribution

$$P(\mathcal{W}) \propto \exp\left(-\frac{1}{2}\beta^{-1}\sigma^{-2}\sum_{t=1}^{T}\|\mathcal{W}_t\|^2 + \frac{1}{2}\beta^{-1}\sum_{t=2}^{T}\lambda\|\mathcal{W}_t - \mathcal{W}_{t-1}\|^2\right) \qquad [2]$$

A. Kernel Functions for Arbitrary λ. The important kernel functions are given by

$$K_{t,t'}^{L,1}(\boldsymbol{x}, \boldsymbol{x}') \equiv \frac{1}{N}\langle\Phi(\mathcal{W}_t, \boldsymbol{x}) \cdot \Phi(\mathcal{W}_{t'}, \boldsymbol{x}')\rangle_{\mathcal{W}} \qquad [3]$$

$$K_{t,t'}^{L,0}(\boldsymbol{x}, \boldsymbol{x}') \equiv \frac{1}{N}\langle\langle\Phi(\mathcal{W}_t, \boldsymbol{x})\rangle_{t,t'} \cdot \langle\Phi(\mathcal{W}_{t'}, \boldsymbol{x}')\rangle_{t',t'}\rangle_{\mathcal{W}} \qquad [4]$$

where $\langle\cdot\rangle_{\mathcal{W}}$ denotes the average over the full prior distribution SI Eq. 2, and $\langle\cdot\rangle_{t,t'}$ denotes the partial average over the conditional distribution $P(\mathcal{W}_t, \mathcal{W}_{t-1}, \cdots, \mathcal{W}_{t'}|\mathcal{W}_{t'-1})$. Furthermore, we introduced

$$\tilde{K}_{t,t'}^{L}(\boldsymbol{x}, \boldsymbol{x}') \equiv m_{t,t'}^1 K_{t,t'}^{L,1}(\boldsymbol{x}, \boldsymbol{x}') - m_{t,t'}^0 K_{t,t'}^{L,0}(\boldsymbol{x}, \boldsymbol{x}') \qquad [5]$$

For $t \geq t' \geq 2$, $m_{t,t'}^1 = \frac{\sigma^2}{1+\tilde{\lambda}}(\tilde{\lambda}^{t-t'} + \tilde{\lambda}^{t+t'-1})$, and $m_{\tau,\tau'}^0 = \frac{\sigma^2}{1+\tilde{\lambda}}(\tilde{\lambda}^{t-t'+2} + \tilde{\lambda}^{t+t'-1})$, where $\tilde{\lambda} \equiv \lambda(\lambda + \sigma^{-2})^{-1}$. Otherwise, $m_{t,1}^1 = \sigma^2\tilde{\lambda}^{t-1}$ and $m_{t,1}^0 = 0$. Finally, we introduced the difference kernel

$$\Delta K_{t,t'}^{L}(\boldsymbol{x}, \boldsymbol{x}') \equiv K_{t,t'}^1(\boldsymbol{x}, \boldsymbol{x}') - K_{t,t'}^0(\boldsymbol{x}, \boldsymbol{x}'). \qquad [6]$$

B. Kernel Functions in the $\lambda \to \infty$ Limit:. In the limit $\lambda \to \infty$, these kernel functions become stationary in time and can be simplified. In particular, $K^{L,1}_{t,t'}(\boldsymbol{x}, \boldsymbol{x}')$ becomes the NNGP kernel, given by

$$K^{L,1}_{t,t'}(\boldsymbol{x}, \boldsymbol{x}') \xrightarrow{\lambda\to\infty} K^{L}_{GP}(\boldsymbol{x}, \boldsymbol{x}') \equiv \Phi(\mathcal{W}_0, \boldsymbol{x}) \cdot \Phi(\mathcal{W}_0, \boldsymbol{x}'), \qquad [7]$$

where $\Phi(\mathcal{W}_0, \boldsymbol{x}) \in \mathbb{R}^N$, and $\mathcal{W}_0 \sim \mathcal{N}(0, \sigma^2 \mathbb{I})$, as introduced in the main text when we introduced the OPs. $\tilde{K}^L_{t,t'}$ $(t, t' \geq 2)$ becomes the NTK, given by

$$\tilde{K}^{L}_{t,t'}(\boldsymbol{x}, \boldsymbol{x}') \xrightarrow{\lambda\to\infty} \lambda^{-1} K^{L}_{NTK}(\boldsymbol{x}, \boldsymbol{x}') \equiv \lambda^{-1} \partial_{\Theta_{random}} f(\Theta_0, \boldsymbol{x}) \cdot \partial_{\Theta_{random}} f(\Theta_0, \boldsymbol{x}') \qquad [8]$$

where $\partial_{\Theta_0} f(\Theta_0, \boldsymbol{x}) \in \mathbb{R}^{N^2(L-1)+NN_0+N}$ is of the dimension of the total number of parameters in the network, and $\Theta_0 \sim \mathcal{N}(0, \sigma^2 \mathbb{I})$. $\Delta K^L_{t,t'}(\boldsymbol{x}, \boldsymbol{x}')$ is given by

$$\Delta K^{L}_{t,t'}(\boldsymbol{x}, \boldsymbol{x}') \xrightarrow{\lambda\to\infty} \lambda^{-1} K^{L}_{NTK}(\boldsymbol{x}, \boldsymbol{x}') \dot{K}^L(\boldsymbol{x}, \boldsymbol{x}') \qquad [9]$$

where

$$\dot{K}^L(\boldsymbol{x}, \boldsymbol{x}') \equiv \phi'(h^L_0(\boldsymbol{x})) \cdot \phi'(h^L_0(\boldsymbol{x}')) \qquad [10]$$

is the derivative kernel. $\left\{h^l_0(\boldsymbol{x})\right\}_{l=1,\cdots,L}$ denote the pre-activation of each layer with random weights $\mathcal{W}_0 = \left\{W^l_0\right\}_{l=1,\cdots,L}$, i.e.,

$$h^l_0(\boldsymbol{x}) \equiv \frac{1}{\sqrt{\dim\left(\boldsymbol{x}^{l-1}\right)}} \sum_j W^{l-1}_{0,ij} x^{l-1}_{0,j}(\boldsymbol{x}) \qquad [11]$$

$$x^l_0(\boldsymbol{x}) = \phi(h^l_0(\boldsymbol{x})) \qquad [12]$$

In the infinite-width limit $N \to \infty$, SI Eqs. 7-10 are all self-averaging, namely, they do not depend on the specific realization of $\mathcal{W}_0$ or Θ_0, and is equivalent to their averages across Gaussian $\mathcal{W}_0$ or Θ_0. See SI 3.B.2 for detailed derivations of the kernel functions and SI 3.B.4 for their analytical expressions in linear and ReLU networks.

Furthermore, to simplify the expression of the statistics of the network's input-output mappings, for each kernel function, we introduce corresponding notations for applying them to the training and testing data, respectively. Specifically, for a kernel function $K^L_{t,t'}(\boldsymbol{x}, \boldsymbol{x}')$, we introduce

$$\boldsymbol{k}^L_{t,t'}(\boldsymbol{x}) \equiv K^L_{t,t'}(\boldsymbol{x}, \boldsymbol{X}_{t'}) \in \mathbb{R}^P \qquad [13]$$

$$\boldsymbol{K}^L_{t,t'} \equiv K^L_{t,t'}(\boldsymbol{X}_t, \boldsymbol{X}_{t'}) \in \mathbb{R}^{P\times P} \qquad [14]$$

$$k^L_{t,t'} \equiv K^L_{t,t'}(\boldsymbol{x}, \boldsymbol{x}) \in \mathbb{R} \qquad [15]$$

where $\boldsymbol{X}_t$ denotes the training data matrix of task t, and $\boldsymbol{x}$ denotes an arbitrary test point. K^L represents the different kernel functions above, including $K^{L,1}, K^{L,0}, \tilde{K}^L$ and ΔK^L.

3. Single-Head Theory

A. Summary of Main Theoretical Results.

A.1. Statistics of the Input-Output Mappings. In single-head CL, the mean input-output mapping after learning T tasks in a network with L hidden layers in the infinite-width limit ($N \to \infty$, $\alpha = P/N \to 0$) is given by

$$\langle f_T(\boldsymbol{x}) \rangle = \sum_{t=1}^{T} \tilde{\boldsymbol{k}}^L_{T,t}(\boldsymbol{x})^\top \langle -i\boldsymbol{v}_t \rangle \qquad [16]$$

$$\langle -i\boldsymbol{v}_t \rangle = \left(\tilde{\boldsymbol{K}}^L_{t,t}\right)^{-1} (\boldsymbol{Y}_t - \sum_{t'=1}^{t-1} \tilde{\boldsymbol{K}}^L_{t,t'} \langle -i\boldsymbol{v}_{\tau'} \rangle) \qquad [17]$$

The equation is applied to evaluate $F_{t,t'} \approx \mathcal{L}(\langle f_T \rangle, D_{t'})$ and $G_{t,t'} \approx \mathcal{L}(\langle f_t \rangle, D^{test}_{t'})/G^0_{t'}$. For simplicity, we assumed small σ such that the variance contribution to $\langle \mathcal{L}(f, D) \rangle$ is neglected, but the expression for the variance of $f_T(\boldsymbol{x})$ is given in SI 3.B.3. These results hold for general λ, in the $\lambda \to \infty$ limit, the kernels $\tilde{K}^L_{t,t'}(t, t' \geq 2)$ are replaced with $\lambda^{-1} K^L_{NTK}$ (SI Eq. 8) and the kernels $\tilde{K}^L_{t,1}$ are replaced with K^L_{GP} (SI Eq. 7).

Although $\mathcal{W}_t$ is still time-dependent in the $\lambda \to \infty$ limit, empirically, we found that the change in $\mathcal{W}_t$ over time is weak, and we can assume that $\mathcal{W}_t \approx \mathcal{W}_0 \sim \mathcal{N}(0, \sigma^2)$ for all t. This results in a simpler dynamical process where only the readout weights $\boldsymbol{a}_t$ change over time, learning on top of the random feature $\Phi(\mathcal{W}_0, \mathbf{x})$. In this case the predictor statistics are simplified, such that all $\tilde{K}^L_{t,t'}$ can be replaced by K^L_{GP}. We refer to this approximation as the random feature approximation. In Fig. 3 and Fig. 4 in the main text and Figs. S2, S5, S6 in SI 9, we showed results with this simplified random feature approximation. In SI 9 Fig. S1 we showed that results under the full Gibbs distribution exhibit qualitatively similar behaviors. In Fig. S3 since we showed results with finite λ, we used $\langle f_T \rangle$ evaluated under the full Gibbs distribution, as given by SI Eqs. 16, 17.

A.2. The Order Parameters. For two tasks ($T=2$), with the random feature approximation in the $\lambda \to \infty$ limit, we obtain a simple expression of $F_{2,1} \approx \mathcal{L}(\langle f_2 \rangle, D_1)$ by plugging in $\langle f_2(\boldsymbol{x}) \rangle$.

$$F_{2,1} = \left(\boldsymbol{Y}_2 - \boldsymbol{K}_{2,1}^{L,1}\left(\boldsymbol{K}_{1,1}^{L,1}\right)^{-1}\boldsymbol{Y}_1\right)^\top \left(\boldsymbol{K}_{2,2}^{L,1}\right)^{-1}\boldsymbol{K}_{2,1}^{L,1}\boldsymbol{K}_{1,2}^{L,1}\left(\boldsymbol{K}_{2,2}^{L,1}\right)^{-1}\left(\boldsymbol{Y}_2 - \boldsymbol{K}_{2,1}^{L,1}\left(\boldsymbol{K}_{1,1}^{L,1}\right)^{-1}\boldsymbol{Y}_1\right)/\|\boldsymbol{Y}_1\|^2 \quad [18]$$

Further assuming that the tasks are symmetric, i.e., the indices are interchangeable in each term, we have

$$F_{2,1} = 2\Bigg(\underbrace{\frac{1}{2}\boldsymbol{Y}_2^\top\left(\boldsymbol{K}_{2,2}^{L,1}\right)^{-1}\boldsymbol{K}_{2,1}^{L,1}\left(\boldsymbol{I}+\left(\boldsymbol{K}_{1,1}^{L,1}\right)^{-1}\boldsymbol{K}_{1,2}^{L,1}\boldsymbol{K}_{2,1}^{L,1}\left(\boldsymbol{K}_{1,1}^{L,1}\right)^{-1}\right)\boldsymbol{K}_{1,2}^{L,1}\left(\boldsymbol{K}_{2,2}^{L,1}\right)^{-1}\boldsymbol{Y}_2/\|\boldsymbol{Y}_2\|^2}_{\gamma_{\mathrm{RF}}} -$$

$$\underbrace{\boldsymbol{Y}_2^\top\left(\boldsymbol{K}_{2,2}^{L,1}\right)^{-1}\boldsymbol{K}_{2,1}^{L,1}\boldsymbol{K}_{1,2}^{L,1}\left(\boldsymbol{K}_{2,2}^{L,1}\right)^{-1}\boldsymbol{K}_{2,1}^{L,1}\left(\boldsymbol{K}_{1,1}^{L,1}\right)^{-1}\boldsymbol{Y}_1/\left(\|\boldsymbol{Y}_1\|\|\boldsymbol{Y}_2\|\right)}_{\gamma_{\mathrm{rule}}}\Bigg) \quad [19]$$

In SI Eqs. 18, 19, $\{\boldsymbol{K}_{i,i'}^{L,1}\}_{i,i'\le 2}$ are equivalent to $\{\boldsymbol{K}_{GP}^{L}(\boldsymbol{X}_i,\boldsymbol{X}_{i'})\}_{i,i'\le 2}$ because $\lambda \to \infty$. SI Eq. 19 is equivalent to Eq. 4 in the main text. In the main text, we expressed the OPs in terms of the rule vectors $\{\boldsymbol{V}_i\}_{i\le 2} \in \mathbb{R}^N$ and the projection matrices $\{\boldsymbol{P}_i\}_{i\le 2} \in \mathbb{R}^{N\times N}$ for better geometric interpretations (Eq. 4). However, as we take the $N\to\infty$ limit, $\{\boldsymbol{V}_i\}_{i\le 2}$ and $\{\boldsymbol{P}_i\}_{i\le 2}$ can not be directly evaluated. In fact, as shown in SI Eq. 19, γ_{RF} and γ_{rule} can be expressed using P dimensional quantities, including the labels $\{\boldsymbol{Y}_i\}_{i\le 2}\in\mathbb{R}^P$ and the GP kernels $\{\boldsymbol{K}_{GP}^{L}(\boldsymbol{X}_i,\boldsymbol{X}_{i'})\}_{i,i'\le 2}\in\mathbb{R}^{P\times P}$, which allows us to compute them given any pair of training data $\{(\boldsymbol{X}_i,\boldsymbol{Y}_i)\}_{i\le 2}$ even as $N\to\infty$. Similarly, the additional OP $\gamma_{\mathrm{feature}}$ can be evaluated using $\gamma_{\mathrm{feature}} = \frac{1}{P}\mathrm{Tr}\left(\left(\boldsymbol{K}_{2,2}^{L,1}\right)^{-1}\boldsymbol{K}_{2,1}^{L,1}\left(\boldsymbol{K}_{1,1}^{L,1}\right)^{-1}\boldsymbol{K}_{1,2}^{L,1}\right)$.

Without any further assumptions, since the matrix $\mathcal{P}_{12}$ as defined in the main text is positive semi-definite, we have $\gamma_{\mathrm{RF}} \ge 0$. It is straightforward that $\gamma_{\mathrm{feature}} \in [0,1]$. Furthermore, given by the Cauchy-Schwarz inequality, we have

$$\begin{aligned}|\gamma_{\mathrm{rule}}| &\le \|\boldsymbol{Y}_2^\top\left(\boldsymbol{K}_{2,2}^{L,1}\right)^{-1}\boldsymbol{K}_{2,1}^{L,1}\|\cdot\|\boldsymbol{K}_{1,2}^{L,1}\left(\boldsymbol{K}_{2,2}^{L,1}\right)^{-1}\boldsymbol{K}_{2,1}^{L,1}\left(\boldsymbol{K}_{1,1}^{L,1}\right)^{-1}\boldsymbol{Y}_1\|/\left(\|\boldsymbol{Y}_1\|\|\boldsymbol{Y}_2\|\right)\\ &= \|\boldsymbol{Y}_2^\top\left(\boldsymbol{K}_{2,2}^{L,1}\right)^{-1}\boldsymbol{K}_{2,1}^{L,1}\|\cdot\|\boldsymbol{K}_{2,1}^{L,1}\left(\boldsymbol{K}_{1,1}^{L,1}\right)^{-1}\boldsymbol{K}_{1,2}^{L,1}\left(\boldsymbol{K}_{2,2}^{L,1}\right)^{-1}\boldsymbol{Y}_2\|/\|\boldsymbol{Y}_2\|^2\\ &\le \frac{1}{2}\left(\|\boldsymbol{Y}_2^\top\left(\boldsymbol{K}_{2,2}^{L,1}\right)^{-1}\boldsymbol{K}_{2,1}^{L,1}\|^2 + \|\boldsymbol{K}_{2,1}^{L,1}\left(\boldsymbol{K}_{1,1}^{L,1}\right)^{-1}\boldsymbol{K}_{1,2}^{L,1}\left(\boldsymbol{K}_{2,2}^{L,1}\right)^{-1}\boldsymbol{Y}_2\|^2\right)/\|\boldsymbol{Y}_2\|^2 = \gamma_{\mathrm{RF}}\end{aligned} \quad [20]$$

The equality follows from the assumption that the tasks are symmetric.

For a long sequence of tasks, we assumed that any pair of tasks in a long sequence of length T have the same relation, i.e.,

$$\mathcal{P}_{tt'} \equiv \frac{1}{2}\left(\boldsymbol{P}_t\left(\boldsymbol{X}_{t'}^L\right)^\top\boldsymbol{X}_{t'}^L\boldsymbol{P}_t + \boldsymbol{P}_{t'}\left(\boldsymbol{X}_t^L\right)^\top\boldsymbol{X}_t^L\boldsymbol{P}_{t'}\right) = \mathcal{P}, t, t' \le T, t \ne t' \quad [21]$$

γ_{rule} can thus be seen as the inner product of two vectors $\tilde{\boldsymbol{V}}_t = \boldsymbol{V}_t^\top\mathcal{P}^{1/2}$ and $\tilde{\boldsymbol{V}}_{t'} = \boldsymbol{V}_{t'}^\top\mathcal{P}^{1/2}$, $t,t'\le T, t\ne t'$. Since any two tasks have the same relation, the vectors $\tilde{\boldsymbol{V}}_t$ and $\tilde{\boldsymbol{V}}_{t'}$ have the same inner product for all $t,t'\le T, t\ne t'$. We also assumed that $\|\tilde{\boldsymbol{V}}_t\| = \|\tilde{\boldsymbol{V}}\|$ for all t. The $T\times T$ covariance matrix of the vectors $\{\tilde{\boldsymbol{V}}_t\}_{t\le T}$ is thus given by

$$\|\tilde{\boldsymbol{V}}\|^2\boldsymbol{I} + \gamma_{\mathrm{rule}}\boldsymbol{1}\boldsymbol{1}^\top \quad [22]$$

where $\boldsymbol{1}$ denotes an N-dimensional all-one vector. It follows from the positive semi-definiteness of this matrix, that $\gamma_{\mathrm{rule}} \ge \|\boldsymbol{V}\|^2/T$. When $T\to\infty$, $\gamma_{\mathrm{rule}} \in [0,\gamma_{\mathrm{RF}}]$, as indicated in Section 3 of the main text, and shown in almost all examples in this paper.

Further assuming that the random features of each task are orthogonal, i.e., $\boldsymbol{K}_{GP}^{L}(\boldsymbol{X}_i,\boldsymbol{X}_i) \propto \boldsymbol{I}$. We have $\gamma_{\mathrm{RF}} = \frac{1}{2}\boldsymbol{V}_2^\top\boldsymbol{P}_1(I+\boldsymbol{P}_2)\boldsymbol{P}_1\boldsymbol{V}_2/\|\boldsymbol{V}_2\|^2$. Since the spectral norm of an orthogonal projection matrix is 1, we have $\gamma_{RF}\in[0,1]$.

B. Detailed Derivations. In this section, we present the detailed derivation for the statistics of input-output mappings in single-head CL in the infinite-width limit.

B.1. Moment Generating Function. We start from the MGF for multi-head CL, given by

$$\mathcal{M}(\ell_T) \equiv \left[Z(D_1)\prod_{t=2}^{T}Z\left(\Theta_{t-1},D_t\right)\right]^{-1}\exp\left(-\beta E(\Theta_1|D_1) - \sum_{t=2}^{T}\beta E\left(\Theta_t|\Theta_{t-1},D_t\right) + i\sum_{t=1}^{T}\ell_T f_T(\mathbf{x})\right) \quad [23]$$

where

$$E\left(\Theta_t|\Theta_{t-1},D_t\right) = \frac{1}{2}\sum_{\mu=1}^{P}\left(f_t\left(\boldsymbol{x}_t^\mu\right)-y_t^\mu\right)^2 + \frac{1}{2}\beta^{-1}\sigma^{-2}\left\|\Theta_t\right\|^2 + \frac{1}{2}\beta^{-1}\lambda\left\|\Theta_t-\Theta_{t-1}\right\|^2. \quad [24]$$

$$E(\Theta_1,D_1) = \frac{1}{2}\sum_{\mu=1}^{P}\left(f_2^1\left(\boldsymbol{x}_1^\mu\right)-y_1^\mu\right)^2 + \frac{1}{2}\beta^{-1}\sigma^{-2}\left\|\Theta_1\right\|^2 \quad [25]$$

and

$$Z\left(\Theta_{t-1}, D_t\right) \equiv \int d\Theta_t \exp(-\beta E(\Theta_t|\Theta_{t-1}, D_t)) \quad [26]$$

$$Z(D_1) \equiv \int d\Theta_1 \exp(-\beta E(\Theta_1, D_1)) \quad [27]$$

Here we introduced field ℓ_T coupled to the mapping after learning all T tasks, the statistics of $f_T(\mathbf{x})$ can therefore be calculated by

$$\langle f_T(\mathbf{x})\rangle = -i\frac{\partial \mathcal{M}(\ell_T)}{\partial \ell_T}|_{\ell_T=0} \quad [28]$$

$$\langle \delta^2 f_T(\mathbf{x})\rangle = -\frac{\partial^2 \mathcal{M}(\ell_T)}{\partial (\ell_T)^2}|_{\ell_T=0} \quad [29]$$

We use the replica method for the denominator in SI Eq. 23, and denote the physical copy of Θ_t as Θ_t^n,we have

$$Z(\Theta_{t-1}, D_t)^{-1} = Z(\Theta_{t-1}^n, D_t)^{-1} = \lim_{n\to 0}\int \prod_{\alpha=1}^{n-1} d\Theta_t^\alpha \exp(-\beta \sum_{\alpha=1}^{n-1} E_t(\Theta_t^\alpha|\Theta_{t-1}^n, D_t)). \quad [30]$$

We then introduce auxiliary integration variable $\{\boldsymbol{v}_t^\alpha\}_{t=2,\cdots,T;\alpha=1,\cdots n}$, $\boldsymbol{v}_1^n \in \mathbb{R}^P$ using the H-S transform, and arrive at

$$\mathcal{M}\left(\ell_T\right) = \lim_{n\to 0}\int \prod_{\alpha=1}^{n}\prod_{t=2}^{T} d\boldsymbol{v}_t^\alpha \int d\boldsymbol{v}_1^n \int \prod_{\alpha=1}^{n}\prod_{t=2}^{T} d\Theta_t^\alpha \int d\Theta_1^n \exp(-i\sum_{t,\alpha}\sum_{\mu=1}^{P} v_t^{\alpha,\mu}\left(f_t\left(\Theta_t^\alpha, \boldsymbol{x}_t^\mu\right) - y_t^\mu\right)$$

$$-\frac{1}{2}\sigma^{-2}\sum_{t,\alpha}\|\Theta_t^\alpha\|^2 - \frac{1}{2}\lambda\sum_{t=2}^{T}\sum_{\alpha=1}^{n}\|\Theta_t^\alpha - \Theta_{t-1}^n\|^2 - \frac{1}{2}\beta^{-1}\sum_{t,\alpha}\boldsymbol{v}_t^{\alpha\top}\boldsymbol{v}_t^\alpha + \sum_{t=1}^{T}\ell_T f_T(\Theta_T^n, \boldsymbol{x})) \quad [31]$$

where we use $f_t\left(\Theta_t^\alpha, \boldsymbol{x}_t^\mu\right)$ to denote the mapping with the replicated Θ_t^α,$f_t(\Theta_t^\alpha, \mathbf{x}_t^\mu) \equiv \frac{1}{\sqrt{N}}\boldsymbol{a}_t^\alpha \Phi(\mathcal{W}_t^\alpha, \mathbf{x}_t^\mu)$; and we use $f_T(\Theta_T^n, \boldsymbol{x})$ to denote the mapping after learning all T tasks on arbitrary test input $\boldsymbol{x}$. Note that $t = 1$ is different from other time indices as we do not introduce replica for $Z_1(D_1)$. Therefore only Θ_1^n (and thus $\boldsymbol{v}_1^n$) appears in the MGF. For notational convenience we define $\sum_{t,\alpha}(\cdot)_{t,\alpha} \equiv (\cdot)_{1,n} + \sum_{t=2}^{T}\sum_{\alpha=1}^{n}(\cdot)_{t,\alpha}$.

We then integrate the readout weights $\{\boldsymbol{a}_t^\alpha\}_{t=1,\cdots T;\alpha=1,\cdots n}$, and obtain in the $\beta \to \infty$ limit

$$\mathcal{M}\left(\ell_T\right) = \lim_{n\to 0}\int \prod_{\alpha=1}^{n}\prod_{t=1}^{T} dv_t^\alpha \exp\left(-\frac{1}{2}\beta^{-1}\sum_{t,\alpha}\boldsymbol{v}_t^{\alpha\top}\boldsymbol{v}_t^\alpha + i\sum_{t,\alpha}\boldsymbol{v}_t^{\alpha\top}\boldsymbol{Y}_t + G(\{\tilde{\boldsymbol{v}}_t^\alpha\}_{\alpha=1,\cdots,n;t=2,\cdots,T}, \tilde{\boldsymbol{v}}_1^n)\right) \quad [32]$$

where

$$G(\{\tilde{\boldsymbol{v}}_t^\alpha\}_{\alpha=1,\cdots,n;t=2,\cdots,T}, \tilde{\boldsymbol{v}}_1^n) \equiv \log\left[\int \prod_{\alpha=1}^{n}\prod_{t=1}^{T} d\mathcal{W}_t^\alpha \exp\left(S_0\left(\{\mathcal{W}^\alpha\}_{\alpha=1,\cdots,n}\right) - \frac{1}{2}\sum_{t,\alpha}\sum_{t',\beta} m_{t,t'}^{\alpha\beta}\tilde{\boldsymbol{v}}_t^{\alpha\top}\boldsymbol{M}_{t,t'}^{\alpha\beta}\tilde{\boldsymbol{v}}_{t'}^\beta\right)\right] \quad [33]$$

$$S_0(\{\mathcal{W}^\alpha\}_{\alpha=1,\cdots,n}) \equiv -\frac{1}{2}\sigma^{-2}\sum_{t,\alpha}\|\mathcal{W}_t^\alpha\|^2 - \frac{1}{2}\lambda\sum_{t=2}^{T}\sum_{\alpha=1}^{n}\|\mathcal{W}_t^\alpha - \mathcal{W}_{t-1}^n\|^2 \quad [34]$$

and

$$\boldsymbol{M}_{t,t'}^{\alpha\beta} \equiv \begin{bmatrix} \frac{1}{N}\Phi(\mathcal{W}_t^\alpha, \boldsymbol{X}_t)\cdot\Phi(\mathcal{W}_{t'}^\beta, \boldsymbol{X}_{t'}) \in \mathbb{R}^{P\times P} & \frac{1}{N}\Phi(\mathcal{W}_t^\alpha, \boldsymbol{X}_t)\cdot\Phi(\mathcal{W}_T^\beta, \mathbf{x}) \in \mathbb{R}^{P\times 1} \\ \frac{1}{N}\Phi(\mathcal{W}_T^\alpha, \mathbf{x})\cdot\Phi(\mathcal{W}_{t'}^\beta, \boldsymbol{X}_{t'}) \in \mathbb{R}^{1\times P} & \frac{1}{N}\Phi(\mathcal{W}_T^\alpha, \mathbf{x})\cdot\Phi(\mathcal{W}_T^\beta, \mathbf{x}) \in \mathbb{R} \end{bmatrix} \in \mathbb{R}^{(P+1)\times(P+1)} \quad [35]$$

For simplicity, here we denote $\tilde{\boldsymbol{v}}_t^\alpha \equiv [\boldsymbol{v}_t^\alpha, \delta_{\alpha n}\delta_{tT}\ell_T] \in \mathbb{R}^{P+1}$, absorbing the field coupled to the mapping on arbitrary $\mathbf{x}$ into $\boldsymbol{v}_t^\alpha$. Since $m_{t,t'}^{\alpha\beta}$ is symmetric in t, t', w.l.o.g., we assume $t \geq t'$. For $t, t' \geq 2$, we have

$$m_{t,t'}^{\alpha\beta} = \begin{cases} m_{t,t'}^1 = \frac{\sigma^2}{1+\tilde{\lambda}}(\tilde{\lambda}^{t-t'} + \tilde{\lambda}^{t+t'-1}) & \{\alpha = \beta, t = t'\} \cup \{\beta = n, t > t'\} \\ m_{\tau,\tau'}^0 = \frac{\sigma^2}{1+\tilde{\lambda}}(\tilde{\lambda}^{t-t'+2} + \tilde{\lambda}^{t+t'-1}) & otherwise \end{cases} \quad [36]$$

where $\tilde{\lambda} \equiv \frac{\lambda}{\lambda+\sigma^{-2}}$. Otherwise we denote

$$m_{t,1}^{\alpha n} = m_{t,1}^1 = \sigma^2\tilde{\lambda}^{t-1}; m_{t,1}^0 \equiv 0 \quad [37]$$

While it is in general highly nontrivial to evaluate G, in the infinite-width limit, the distribution of $\mathcal{W}$ is dominated by the Gaussian prior determined by $S_0(\mathcal{W})$, and the weights become self-averaging. G is thus given by

$$G(\{\tilde{\boldsymbol{v}}_t^\alpha\}_{\alpha=1,\cdots,n;t=2,\cdots,T}, \tilde{\boldsymbol{v}}_1^n) = -\frac{1}{2}\sum_{t,\alpha}\sum_{t,\beta} m_{t,t'}^{\alpha\beta}\tilde{\boldsymbol{v}}_t^{\alpha\top}\langle \boldsymbol{M}_{t,t'}^{\alpha\beta}\rangle_{\mathcal{W}}\tilde{\boldsymbol{v}}_{t'}^\beta \quad [38]$$

where $\langle\cdot\rangle_{\mathcal{W}}$ denotes averaging over the prior Gaussian distribution proportional to $\exp(-S_0(\{\mathcal{W}^\alpha\}_{\alpha=1,\cdots,n}))$.

B.2. Definition of Kernel Functions. We observe that due to the structure of the prior distribution, $\langle \boldsymbol{M}^{\alpha\beta}_{t,t'}\rangle_{\mathcal{W}}$ can be expressed by two different kernel functions, defined on arbitrary inputs $\boldsymbol{x}$ and $\boldsymbol{x}'$.The kernel functions are symmetric in t and t', so w.l.o.g. we define them with $t \geq t'$.

$$K^{L,1}_{t,t'}(\boldsymbol{x},\boldsymbol{x}') \equiv \frac{1}{N}\langle \Phi(\mathcal{W}_t,\boldsymbol{x})\cdot\Phi(\mathcal{W}_{t'},\boldsymbol{x}')\rangle_{\mathcal{W}} \qquad [39]$$

$$K^{L,0}_{t,t'}(\boldsymbol{x},\boldsymbol{x}') \equiv \frac{1}{N}\langle\langle \Phi(\mathcal{W}_t,\boldsymbol{x})\rangle_{t,t'}\cdot\langle\Phi(\mathcal{W}_{t'},\boldsymbol{x}')\rangle_{t,t'}\rangle_{\mathcal{W}} \qquad [40]$$

where $\langle\cdot\rangle_{\mathcal{W}}$ denotes the average over the full prior distribution SI Eq. 2, and $\langle\cdot\rangle_{t,t'}$ denotes the partial average over the conditional distribution $P(\mathcal{W}_t,\mathcal{W}_{t-1},\cdots,\mathcal{W}_{t'}|\mathcal{W}_{t'-1})$.

$\langle \boldsymbol{M}^{\alpha\beta}_{t,t'}\rangle_{\mathcal{W}}$ can be expressed as

$$\langle \boldsymbol{M}^{\alpha\beta}_{t,t'}\rangle_{\mathcal{W}} = \begin{bmatrix} \boldsymbol{K}^{L,\alpha\beta}_{t,t'} \in \mathbb{R}^{P\times P} & \boldsymbol{k}^{L,\alpha n}_{t,T}(\boldsymbol{x}) \in \mathbb{R}^{P\times 1} \\ \boldsymbol{k}^{L,n\beta}_{T,t'}(\boldsymbol{x}) \in \mathbb{R}^{1\times P} & k^{L,nn}_{T,T}(\boldsymbol{x},\boldsymbol{x}) \in \mathbb{R} \end{bmatrix} \qquad [41]$$

with $\boldsymbol{K}^{L,\alpha\beta}_{t,t'}$,$\boldsymbol{k}^{L,\alpha n}_{t,T}(\boldsymbol{x})$,$\boldsymbol{k}^{L,n\beta}_{T,t}(\boldsymbol{x})$ and $k^{L,nn}_{T,T}(\boldsymbol{x},\boldsymbol{x})$ denoting the 4 blocks corresponding to SI Eq. 35. They are given by applying the kernel functions on the training and testing data, respectively.Again since $\langle \boldsymbol{M}^{\alpha\beta}_{t,t'}\rangle_{\mathcal{W}}$ is symmetric in t and t', w.l.o.g. for $t \geq t'$, we have

$$\boldsymbol{K}^{L,\alpha\beta}_{t,t'} = \begin{cases} \boldsymbol{K}^{L,1}_{t,t'} \equiv K^{L,1}_{t,t'}(\boldsymbol{X}_t,\boldsymbol{X}_{t'}) & \{\alpha=\beta, t=t'\}\cup\{\beta=n, t>t'\} \\ \boldsymbol{K}^{L,0}_{t,t'} \equiv K^{L,0}_{t,t'}(\boldsymbol{X}_t,\boldsymbol{X}_{t'}) & otherwise \end{cases} \qquad [42]$$

$$\boldsymbol{k}^{L,\alpha n}_{t,T}(\boldsymbol{x}) = \begin{cases} \boldsymbol{k}^{L,1}_{t,T}(\boldsymbol{x}) \equiv K^{L,1}_{t,T}(\boldsymbol{X}_t,\boldsymbol{x}) & \alpha = n \\ \boldsymbol{k}^{L,0}_{t,T}(\boldsymbol{x}) \equiv K^{L,0}_{t,T}(\boldsymbol{X}_t,\boldsymbol{x}) & otherwise \end{cases} \qquad [43]$$

$$k^{L,nn}_{T,T}(\boldsymbol{x},\boldsymbol{x}) = k^{L,1}_{T,T}(\boldsymbol{x},\boldsymbol{x}) \equiv K^{L,1}_{T,T}(\boldsymbol{x},\boldsymbol{x}) \qquad [44]$$

We introduced notations $\boldsymbol{K}^{L,1}_{t,t'}$,$\boldsymbol{K}^{L,0}_{t,t'}$,$\boldsymbol{k}^{L,1}_{t,T}(\boldsymbol{x})$, $\boldsymbol{k}^{L,0}_{t,T}(\boldsymbol{x})$ and $k^{L,1}_{T,T}(\boldsymbol{x},\boldsymbol{x})$, for applying the kernel functions (SI Eqs. 39, 40) on the training and testing data.

For notational convenience, we introduce another kernel function as it will appear frequently in the statistics of input-output mappings

$$\tilde{K}^{L}_{t,t'}(\boldsymbol{x},\boldsymbol{x}') \equiv m^1_{t,t'}K^{L,1}_{t,t'}(\boldsymbol{x},\boldsymbol{x}') - m^0_{t,t'}K^{L,0}_{t,t'}(\boldsymbol{x},\boldsymbol{x}') \qquad [45]$$

Applying this kernel function on the training and testing data, we have $\tilde{\boldsymbol{K}}^L_{t,t'} = m^1_{t,t'}\boldsymbol{K}^{L,1}_{t,t'} - m^0_{t,t'}\boldsymbol{K}^{L,0}_{t,t'}$, $\tilde{\boldsymbol{k}}^L_{t,t'}(\boldsymbol{x}) = m^1_{t,t'}\boldsymbol{k}^{L,1}_{t,t'}(\boldsymbol{x}) - m^0_{t,t'}\boldsymbol{k}^{L,0}_{t,t'}(\boldsymbol{x})$. We will use the same notations for these kernel functions and for applying them on training and testing data throughout the supplementary. Interestingly, in the limit $\lambda\to\infty$, $\tilde{K}^L_{t,t'}(\boldsymbol{x},\boldsymbol{x}')$ corresponds to a generalized two-times Neural Tangent Kernel, as we will show in SI 3.B.5.

B.3. Derivation of the Statistics of Input-Output Mappings. With the above definition of the kernel functions, we can replace $\langle \boldsymbol{M}^{\alpha\beta}_{t,t'}\rangle_{\mathcal{W}}$ with the corresponding kernels, and thus rewriting $\mathcal{M}(\ell_T)$ as

$$\begin{aligned} \mathcal{M}(\ell_T) = \lim_{n\to 0}\int \prod_{\alpha=1}^{n}\prod_{t=1}^{T} d\boldsymbol{v}^\alpha_t \exp(-\frac{1}{2}\beta^{-1}\sum_{t,\alpha}\boldsymbol{v}^{\alpha\top}_t\boldsymbol{v}^\alpha_t + i\sum_{t,\alpha}\boldsymbol{v}^{\alpha\top}_t\boldsymbol{Y}_t - \frac{1}{2}\sum_{t,\alpha}\boldsymbol{v}^\alpha_t\tilde{\boldsymbol{K}}^L_{t,t'}\boldsymbol{v}^\alpha_t - \frac{1}{2}\sum_{t=2}^{T}\sum_{\alpha,\beta=1}^{n} m^0_{t,t}\boldsymbol{v}^{\alpha\top}_t\boldsymbol{K}^{L,0}_{t,t}\boldsymbol{v}^\beta_t \\ - \sum_{t=t'+1}^{T}\sum_{t'=1}^{T}\sum_{\alpha=1}^{n}\boldsymbol{v}^{\alpha\top}_t\tilde{\boldsymbol{K}}^L_{t,t'}\boldsymbol{v}^n_{t'} - \sum_{t=t'+1}^{T}\sum_{t'=2}^{T}\sum_{\alpha,\beta=1}^{n} m^0_{t,t'}\boldsymbol{v}^\alpha_t\boldsymbol{K}^{L,0}_{t,t'}\boldsymbol{v}^\beta_{t'} \\ - \ell_T\sum_{t=1}^{T}\tilde{\boldsymbol{k}}^L_{T,t}(\boldsymbol{x})\boldsymbol{v}^n_t - \sum_{t=2}^{T}\sum_{\beta=1}^{n} m^0_{T,t}\ell_T\boldsymbol{k}^{L,0}_{T,t}(\boldsymbol{x})\boldsymbol{v}^\beta_t - \frac{1}{2}\ell_T^2 m^1_{T,T}k^{L,1}_{T,T}(\boldsymbol{x},\boldsymbol{x})) \end{aligned} \qquad [46]$$

The remaining calculation is to integrate over $\{\boldsymbol{v}^\alpha_t\}_{t=1,\cdots,T;\alpha=1,\cdots,n}$. To decouple the replica indices, we introduce $\boldsymbol{p}_t = \sum_{\alpha=1}^n \boldsymbol{v}^\alpha_t$, and its corresponding conjugate variable $\boldsymbol{q}_t$. Using Fourier representation of the Dirac delta function $\delta(\boldsymbol{p}_t - \sum_{\alpha=1}^n \boldsymbol{v}^\alpha_t) =$

$\int d\boldsymbol{q}_t \exp\left(i\boldsymbol{q}_t\left(\boldsymbol{p}_t - \sum_{\alpha=1}^{n}\boldsymbol{v}_t^{\alpha}\right)\right)$, we rewrite $\mathcal{M}(\ell_T)$ as

$$\mathcal{M}\left(\ell_T\right) = \lim_{n\to 0}\int d\boldsymbol{v}_1 \int \prod_{\alpha=1}^{n}\prod_{t=2}^{T} d\boldsymbol{v}_t^{\alpha} \int \prod_{t=1}^{T} d\boldsymbol{p}_t \int \prod_{t=1}^{T} d\boldsymbol{q}_t \exp\Bigg[-\frac{1}{2}\beta^{-1}\sum_{t,\alpha}\boldsymbol{v}_t^{\alpha\top}\boldsymbol{v}_t^{\alpha} + i\sum_{t=2}^{T}\boldsymbol{p}_t^{\top}\boldsymbol{Y}_t + i\boldsymbol{v}_1^{\top}\boldsymbol{Y}_1 \tag{47}$$

$$+ i\sum_{t=2}^{T}\boldsymbol{q}_t(\boldsymbol{p}_t - \sum_{\alpha=1}^{n}\boldsymbol{v}_t^{\alpha}) - \frac{1}{2}\sum_{t,\alpha}\boldsymbol{v}_t^{\alpha}\tilde{\boldsymbol{K}}_{t,t}^{L}\boldsymbol{v}_t^{\alpha} - \frac{1}{2}\sum_{t=2}^{T} m_{t,t}^{0}\boldsymbol{p}_t^{\top}\boldsymbol{K}_{t,t}^{L,0}\boldsymbol{p}_t$$

$$- \sum_{t=t'+1}^{T}\sum_{t'=1}^{T}\boldsymbol{p}_t^{\top}\tilde{\boldsymbol{K}}_{t,t'}^{L}\boldsymbol{v}_{t'} - \sum_{t=t'+1}^{T}\sum_{t'=2}^{T} m_{t,t'}^{0}\boldsymbol{p}_t^{\top}\boldsymbol{K}_{t,t'}^{L,0}\boldsymbol{p}_{t'}$$

$$- \ell_T\sum_{t=1}^{T}\tilde{\boldsymbol{k}}_{T,t}^{L}(\boldsymbol{x})\boldsymbol{v}_t^{n} - \sum_{t=2}^{T} m_{T,t}^{0}\ell_T\boldsymbol{k}_{T,t}^{L,0}(\boldsymbol{x})\boldsymbol{p}_t - \frac{1}{2}\ell_T^{2} m_{T,T}^{1} k_{T,T}^{L,1}(\boldsymbol{x},\boldsymbol{x})\Bigg] \tag{48}$$

We note that the different replica indices have been decoupled, which allows us to integrate over $\{\boldsymbol{v}_t^{\alpha}\}_{\alpha=1,\cdots,n}$ independently. Let $\boldsymbol{v}_t \equiv \boldsymbol{v}_t^{n}$, integrate over $\{\boldsymbol{v}_t^{\alpha}\}_{\alpha=1}^{n-1}$, and keep only the $\mathcal{O}(1)$ terms (neglecting $\mathcal{O}(n)$ contributions), we have

$$\mathcal{M}\left(\ell_T\right) = \int \prod_{t=2}^{T} d\boldsymbol{p}_t \int \prod_{t=1}^{T} d\boldsymbol{v}_t \exp\Bigg[-\frac{1}{2}\sum_{t=2}^{T}\boldsymbol{p}_t^{\top}(m_{t,t}^{0}\boldsymbol{K}_{t,t}^{L,0} - \tilde{\boldsymbol{K}}_{t,t}^{L})\boldsymbol{p}_t - \frac{1}{2}m_{1,1}^{1}\boldsymbol{v}_1^{\top}\boldsymbol{K}_{1,1}^{L,1}\boldsymbol{v}_1$$

$$- \sum_{t=t'}^{T}\sum_{t'=2}^{T}\boldsymbol{p}_t^{\top}\tilde{\boldsymbol{K}}_{t,t'}^{L}\boldsymbol{v}_{t'} - \sum_{t=t'+1}^{T}\sum_{t'=2}^{T} m_{t,t'}^{0}\boldsymbol{p}_t^{\top}\boldsymbol{K}_{t,t'}^{L,0}\boldsymbol{p}_{t'} - \sum_{t=2}^{T} m_{t,1}^{1}\boldsymbol{v}_1^{\top}\boldsymbol{K}_{1,t}^{L,1}\boldsymbol{p}_t + i\sum_{t=2}^{T}\boldsymbol{p}_t^{\top}\boldsymbol{Y}_t + i\boldsymbol{v}_1^{\top}\boldsymbol{Y}_1$$

$$- \frac{1}{2}\ell_T^{2} m_{T,T}^{1} k_{T,T}^{L,1}(\boldsymbol{x},\boldsymbol{x}) - \ell_T\sum_{t=2}^{T} m_{t,T}^{0}\boldsymbol{k}_{T,t}^{L,0}(\boldsymbol{x})\boldsymbol{p}_t - \ell_T\sum_{t=1}^{T}\tilde{\boldsymbol{k}}_{T,t}^{L}(\boldsymbol{x})\boldsymbol{v}_t\Bigg] \tag{49}$$

We have eliminated all the replica indices, allowing us to proceed to computing the mapping statistics.

The Mean Input-Output Mappings The average mapping can be obtained by taking derivative of $\mathcal{M}(\ell_T)$ w.r.t. ℓ_T, resulting in

$$\langle f_T(\boldsymbol{x})\rangle = \sum_{t=2}^{T} m_{T,t}^{0}\boldsymbol{k}_{T,t}^{L,0}(\boldsymbol{x})\langle -i\boldsymbol{p}_t\rangle + \sum_{t=1}^{T}\tilde{\boldsymbol{k}}_{T,t}^{L}(\boldsymbol{x})\langle -i\boldsymbol{v}_t\rangle \tag{50}$$

with the statistics of $\boldsymbol{p}_t$ and $\boldsymbol{v}_t$ determined by $\mathcal{M}(\ell_T = 0)$, resulting in $\langle \boldsymbol{p}_t\rangle = 0$ and

$$\langle -i\boldsymbol{v}_t\rangle = \left(\tilde{\boldsymbol{K}}_{t,t}^{L}\right)^{-1}(\boldsymbol{Y}_t - \sum_{t'=1}^{t-1}\tilde{\boldsymbol{K}}_{t,t'}^{L}\langle -i\boldsymbol{v}_{\tau'}\rangle) \tag{51}$$

The mean mapping thus simplifies to

$$\langle f_T(\boldsymbol{x})\rangle = \sum_{t=1}^{T}\tilde{\boldsymbol{k}}_{T,t}^{L}(\boldsymbol{x})\langle -i\boldsymbol{v}_t\rangle. \tag{52}$$

The Variance of the Input-Output Mappings The variance of the mapping can be evaluated by taking the second derivative of $\mathcal{M}(\ell_T)$ w.r.t. ℓ_T, resulting in

$$\langle \delta^2 f_T(\boldsymbol{x})\rangle = m_{T,T}^{1} k_{T,T}^{L,1}(\boldsymbol{x},\boldsymbol{x}) - \sum_{t,t'=2}^{T}\left(m_{T,t}^{0}\right)^2\boldsymbol{k}_{T,t}^{L,0}(\boldsymbol{x})\langle \delta\boldsymbol{p}_t\delta\boldsymbol{p}_{t'}^{\top}\rangle\boldsymbol{k}_{T,t}^{L,0}(\boldsymbol{x})^{\top}$$

$$- \sum_{t,t'=1}^{T}\tilde{\boldsymbol{k}}_{T,t}^{L}(\boldsymbol{x})\langle \delta\boldsymbol{v}_t\delta\boldsymbol{v}_{t'}^{\top}\rangle\tilde{\boldsymbol{k}}_{T,t'}^{L}(\boldsymbol{x})^{\top} - 2\sum_{t=2}^{T}\sum_{t'=1}^{T} m_{t,T}^{0}\boldsymbol{k}_{T,t}^{L,0}(\boldsymbol{x})\langle \delta\boldsymbol{p}_t\delta\boldsymbol{v}_{t'}^{\top}\rangle\tilde{\boldsymbol{k}}_{T,t'}^{L}(\boldsymbol{x})^{\top} \tag{53}$$

The statistics of $\boldsymbol{p}_t$ and $\boldsymbol{v}_t$ is again determined by $\mathcal{M}(\ell_T = 0)$, and we have $\langle \delta\boldsymbol{p}_t\delta\boldsymbol{p}_{t'}^{\top}\rangle = 0$

$$\langle \delta\boldsymbol{p}_t\delta\boldsymbol{v}_{t'}^{\top}\rangle = \begin{cases} 0 & t' < t \\ \left(\tilde{\boldsymbol{K}}_{t,t}^{L}\right)^{-1} & t = t' \\ -\left(\tilde{\boldsymbol{K}}_{t,t}^{L}\right)^{-1}\left(\sum_{\tau=t+1}^{t'}\tilde{\boldsymbol{K}}_{t,\tau}^{L}\langle \delta\boldsymbol{p}_{\tau}\delta\boldsymbol{v}_{t'}^{\top}\rangle\right) & t' > t \end{cases} \tag{54}$$

$\langle\delta\boldsymbol{v}_t\delta\boldsymbol{v}_{t'}^{\top}\rangle$ is symmetric in t,t', so we show $\langle\delta\boldsymbol{v}_t\delta\boldsymbol{v}_{t'}^{\top}\rangle$ only for $t\leq t'$

$$\langle\delta\boldsymbol{v}_t\delta\boldsymbol{v}_{t'}^{\top}\rangle=-\left(\tilde{\boldsymbol{K}}_{t,t}^{L}\right)^{-1}(\sum_{\tau=2}^{t-1}\tilde{\boldsymbol{K}}_{t,\tau}^{L}\langle\delta\boldsymbol{v}_\tau\delta\boldsymbol{v}_{t'}^{\top}\rangle+(m_{t,t}^{0}\boldsymbol{K}_{t,t}^{L,0}-\tilde{\boldsymbol{K}}_{t,t}^{L})\langle\delta\boldsymbol{p}_t\delta\boldsymbol{v}_{t'}^{\top}\rangle$$

$$+\sum_{\tau=2}^{t'}(1-\delta_{\tau,t})m_{t,\tau}^{0}\boldsymbol{K}_{t,\tau}^{L,0}\langle\delta\boldsymbol{p}_\tau\delta\boldsymbol{v}_{t'}^{\top}\rangle+(1-\delta_{1,t})m_{1,t}^{1}\boldsymbol{K}_{t,1}^{L,1}\langle\delta\boldsymbol{p}_1\delta\boldsymbol{v}_{t'}^{\top}\rangle) \quad [55]$$

Thus the variance can be simplified as

$$\langle\delta^2 f_T(\boldsymbol{x})\rangle=m_{T,T}^{1}k_{T,T}^{L,1}(\boldsymbol{x},\boldsymbol{x})-\sum_{t,t'=1}^{T}\tilde{\boldsymbol{k}}_{T,t}^{L}(\boldsymbol{x})\langle\delta\boldsymbol{v}_t\delta\boldsymbol{v}_{t'}^{\top}\rangle\tilde{\boldsymbol{k}}_{T,t'}^{L}(\boldsymbol{x})^{\top}$$

$$-2\sum_{t'=1}^{T}\sum_{t=2}^{t'}m_{t,T}^{0}\boldsymbol{k}_{T,t}^{L,0}(\boldsymbol{x})\langle\delta\boldsymbol{p}_t\delta\boldsymbol{v}_{t'}^{\top}\rangle\tilde{\boldsymbol{k}}_{T,t'}^{L}(\boldsymbol{x})^{\top} \quad [56]$$

The variance can therefore be calculated iteratively. Since $\left\{m_{t,t'}^{1}\right\}_{t,t'=1,\cdots,T}$, $\left\{m_{t,t'}^{0}\right\}_{t,t'=1,\cdots,T}$ scales as σ^2 , and the GP kernels scale as σ^{2L}, the variance scales with $\sigma^{2(L+1)}$, therefore when σ is small, the variance contribution can be neglected. For simplicity, we focus on the contribution of the bias term to the performance, namely $\langle\mathcal{L}(f_T,D)\rangle\approx\mathcal{L}(\langle f_T\rangle,D)$.

B.4. Analytical Forms of Kernel Functions in Linear and ReLU Neurons. The kernel functions in SI 3.B.2 can be evaluated iteratively across layers, using

$$K_{t,t'}^{L,1}(\boldsymbol{x},\boldsymbol{x}')=F(m_{t,t}^{1}K_{t,t}^{L-1,1}(\boldsymbol{x},\boldsymbol{x}),m_{t',t'}^{1}K_{t',t'}^{L-1,1}(\boldsymbol{x}',\boldsymbol{x}'),m_{t,t'}^{L-1,1}K_{t,t'}^{L-1,1}(\boldsymbol{x},\boldsymbol{x}')) \quad [57]$$

$$K_{t,t'}^{L,0}(\boldsymbol{x},\boldsymbol{x}')=F(m_{t,t}^{1}K_{t,t}^{L-1,1}(\boldsymbol{x},\boldsymbol{x}),m_{t',t'}^{1}K_{t',t'}^{L-1,1}(\boldsymbol{x}',\boldsymbol{x}'),m_{t,t'}^{L-1,0}K_{t,t'}^{L-1,0}(\boldsymbol{x},\boldsymbol{x}')) \quad [58]$$

with the initial conditions

$$K_{t,t'}^{L=0,1}(\boldsymbol{x},\boldsymbol{x}')=K_{t,t'}^{L=0,0}(\boldsymbol{x},\boldsymbol{x}')=N_0^{-1}\boldsymbol{x}\cdot\boldsymbol{x}' \quad [59]$$

The function $F(\mathbb{E}[z^2],\mathbb{E}[z^{'2}],\mathbb{E}[zz'])$ is a function of the variances of two Gaussian variables z and z' and their covariance. The form of F depends on the nonlinearity of the network (4). F has analytical forms for certain types of nonlinearities ϕ. In this paper we show results for networks with linear or ReLU nonlinearities. We present the analytical forms of the kernels in this section.

For linear networks,

$$K_{t,t'}^{L,0}(\boldsymbol{x},\boldsymbol{x}')=N_0^{-1}\left(m_{t,t'}^{0}\right)^{L}\boldsymbol{x}\cdot\boldsymbol{x}' \quad [60]$$

$$K_{t,t'}^{L,1}(\boldsymbol{x},\boldsymbol{x}')=N_0^{-1}\left(m_{t,t'}^{1}\right)^{L}\boldsymbol{x}\cdot\boldsymbol{x}' \quad [61]$$

$$\tilde{K}_{t,t'}^{L}(\boldsymbol{x},\boldsymbol{x}')=N_0^{-1}\left(\left(m_{t,t'}^{1}\right)^{L+1}-\left(m_{t,t'}^{0}\right)^{L+1}\right)\boldsymbol{x}\cdot\boldsymbol{x}' \quad [62]$$

In the $\lambda\to\infty$ limit, $\tilde{K}_{t,t'}^{L}(\boldsymbol{x},\boldsymbol{x}')$ scales with λ^{-1}, and can be given by

$$\tilde{K}_{t,t'}^{L}(\boldsymbol{x},\boldsymbol{x}')=N_0^{-1}(L+1)\lambda^{-1}\boldsymbol{x}\cdot\boldsymbol{x}' \quad [63]$$

For ReLU nonlinearity, we first define the function

$$J(\theta)=(\pi-\theta)\cos(\theta)+\sin(\theta) \quad [64]$$

Then we have

$$K_{t,t'}^{L,0}(\boldsymbol{x},\boldsymbol{x}')=\frac{\sqrt{K_{t,t}^{L-1,1}(\boldsymbol{x},\boldsymbol{x})K_{t',t'}^{L-1,1}(\boldsymbol{x}',\boldsymbol{x}')}}{2\pi}J(\theta_{t,t'}^{L-1,0}(\boldsymbol{x},\boldsymbol{x}')) \quad [65]$$

$$K_{t,t'}^{L,1}(\boldsymbol{x},\boldsymbol{x}')=\frac{\sqrt{K_{t,t}^{L-1,1}(\boldsymbol{x},\boldsymbol{x})K_{t',t'}^{L-1,1}(\boldsymbol{x}',\boldsymbol{x}')}}{2\pi}J(\theta_{t,t'}^{L-1,1}(\boldsymbol{x},\boldsymbol{x}')) \quad [66]$$

where

$$\theta_{t,t'}^{L,0}(\boldsymbol{x},\boldsymbol{x}')=\cos^{-1}\left(\frac{m_{t,t'}^{0}K_{t,t'}^{L,0}(\boldsymbol{x},\boldsymbol{x}')}{\sqrt{m_{t,t}^{1}K_{t,t}^{L,1}(\boldsymbol{x},\boldsymbol{x})}\sqrt{m_{t',t'}^{1}K_{t',t'}^{L,1}(\boldsymbol{x}',\boldsymbol{x}')}}\right) \quad [67]$$

and

$$\theta_{t,t'}^{L,1}(\boldsymbol{x},\boldsymbol{x}')=\cos^{-1}\left(\frac{m_{t,t'}^{1}K_{t,t'}^{L,1}(\boldsymbol{x},\boldsymbol{x}')}{\sqrt{m_{t,t}^{1}K_{t,t}^{L,1}(\boldsymbol{x},\boldsymbol{x})}\sqrt{m_{t',t'}^{1}K_{t',t'}^{L,1}(\boldsymbol{x}',\boldsymbol{x}')}}\right) \quad [68]$$

with the initial condition that

$$K_{t,t'}^{L=0,0}(\boldsymbol{x},\boldsymbol{x}') = K_{t,t'}^{L=0,1}(\boldsymbol{x},\boldsymbol{x}') = N_0^{-1}\boldsymbol{x}\cdot\boldsymbol{x}' \quad [69]$$

As usual we have $\tilde{K}_{t,t'}^{L}(\boldsymbol{x},\boldsymbol{x}') = m_{t,t'}^{1}K_{t,t'}^{L,1}(\boldsymbol{x},\boldsymbol{x}') - m_{t,t'}^{0}K_{t,t'}^{L,0}(\boldsymbol{x},\boldsymbol{x}')$.In the $\lambda\to\infty$ limit, $\tilde{K}_{t,t'}^{L}(\boldsymbol{x},\boldsymbol{x}')$ scales with λ^{-1}, and is given iteratively by

$$\tilde{K}_{t,t'}^{L}(\boldsymbol{x},\boldsymbol{x}') = \lambda^{-1}K_{t,t'}^{L,1}(\boldsymbol{x},\boldsymbol{x}') + \sigma^2\frac{1}{2\pi}(\pi - \theta_{t,t'}^{L-1,1}(\boldsymbol{x},\boldsymbol{x}'))\tilde{K}_{t,t'}^{L-1}(\boldsymbol{x},\boldsymbol{x}') \quad [70]$$

with initial condition

$$\tilde{K}_{t,t'}^{L=0}(\boldsymbol{x},\boldsymbol{x}') = \lambda^{-1}N_0^{-1}\boldsymbol{x}\cdot\boldsymbol{x}' \quad [71]$$

B.5. $\tilde{K}_{t,t'}^{L}$ ***and the Neural Tangent Kernel.*** In this section, we show that the kernel function $\tilde{K}_{t,t'}^{L}(\boldsymbol{x},\boldsymbol{x}')$ defined in SI Eq. 45 and appearing in the input-output mapping statistics in SI 3.B.3 is closely related the the neural tangent kernel (1), in the limit $\lambda\to\infty$.

Iterative Expression of $\tilde{K}_{t,t'}^{L}$ First, we derive an iterative expression of $\tilde{K}_{t,t'}^{L}(\boldsymbol{x},\boldsymbol{x}')$ in the $\lambda\to\infty$ limit. By expanding in $\mathcal{O}(\lambda^{-1})$, we can rewrite the $\tilde{K}_{t,t'}^{L}(\boldsymbol{x},\boldsymbol{x}')$ as

$$\tilde{K}_{t,t'}^{L}(\boldsymbol{x},\boldsymbol{x}') = \lambda^{-1}K_{t,t'}^{L,1}(\boldsymbol{x},\boldsymbol{x}') + \sigma^2\Delta K_{t,t'}^{L}(\boldsymbol{x},\boldsymbol{x}') \quad [72]$$

where $\Delta K_{t,t'}^{L}(\boldsymbol{x},\boldsymbol{x}')$ is defined as

$$\Delta K_{t,t'}^{L}(\boldsymbol{x},\boldsymbol{x}') \equiv K_{t,t'}^{L,1}(\boldsymbol{x},\boldsymbol{x}') - K_{t,t'}^{L,0}(\boldsymbol{x},\boldsymbol{x}') \quad [73]$$

Applying and expanding SI Eqs. 57, 58, we have

$$\Delta K_{t,t'}^{L}(\boldsymbol{x},\boldsymbol{x}') = \dot{K}_{t,t'}^{L}(\boldsymbol{x},\boldsymbol{x}')\tilde{K}_{t,t'}^{L-1}(\boldsymbol{x},\boldsymbol{x}') \quad [74]$$

$$\dot{K}_{t,t'}^{L}(\boldsymbol{x},\boldsymbol{x}') \equiv \langle\phi'(h_t^L(\boldsymbol{x}))\cdot\phi'(h_{t'}^L(\boldsymbol{x}'))\rangle \quad [75]$$

where $h_t^L(\boldsymbol{x}) \equiv N^{-1/2}W_t^L\cdot\boldsymbol{x}$ is the pre-activation at the L-th layer.Thus we have an iterative relation

$$\tilde{K}_{t,t'}^{L}(\boldsymbol{x},\boldsymbol{x}') = \lambda^{-1}K_{t,t'}^{L,1}(\boldsymbol{x},\boldsymbol{x}') + \sigma^2\dot{K}_{t,t'}^{L}(\boldsymbol{x},\boldsymbol{x}')\tilde{K}_{t,t'}^{L-1}(\boldsymbol{x},\boldsymbol{x}') \quad [76]$$

with initial condition

$$\tilde{K}_{t,t'}^{L=0}(\boldsymbol{x},\boldsymbol{x}') = \lambda^{-1}N_0^{-1}\boldsymbol{x}\cdot\boldsymbol{x}' \quad [77]$$

Note that in the $\lambda\to\infty$ limit both $K_{t,t'}^{L,1}$ and $\dot{K}_{t,t'}^{L}$ are independent of time. Therefore $\tilde{K}_{t,t'}^{L}(\boldsymbol{x},\boldsymbol{x}')$ is also independent of time.Thus we have

$$\tilde{K}^{L}(\boldsymbol{x},\boldsymbol{x}') = \lambda^{-1}K_{GP}^{L}(\boldsymbol{x},\boldsymbol{x}') + \sigma^2\dot{K}^{L}(\boldsymbol{x},\boldsymbol{x}')\tilde{K}^{L-1}(\boldsymbol{x},\boldsymbol{x}') \quad [78]$$

Relation to the Neural Tangent Kernel: Next, we note that the neural tangent kernel (NTK), is given by

$$K^{L,NTK}(\boldsymbol{x},\boldsymbol{x}') = \langle\partial_{\Theta_{random}}f(\Theta_{random},\boldsymbol{x})\cdot\partial_{\Theta_{random}}f(\Theta_{random},\boldsymbol{x}')\rangle \quad [79]$$

where the average is w.r.t. Gaussian random $\Theta_{random}\sim\mathcal{N}(0,\sigma^2\mathbb{I})$. We aim to show that $K^{L,NTK}(\boldsymbol{x},\boldsymbol{x}')$ obeys the same relation as $\lambda\tilde{K}^{L}(\boldsymbol{x},\boldsymbol{x}')$, given by SI Eq. 78. To this end, we separate SI Eq. 79 into two parts, derivative w.r.t. the readout weights, and derivative w.r.t. the hidden-layer weights.

- Derivative w.r.t. the readout weights:

$$\langle\partial_{\boldsymbol{a}_{random}}f(\Theta_{random},\boldsymbol{x})\cdot\partial_{\boldsymbol{a}_{random}}f(\Theta_{random},\boldsymbol{x}')\rangle = K_{GP}^{L}(\boldsymbol{x},\boldsymbol{x}') \quad [80]$$

- Derivative w.r.t. the hidden-layer weights:
  Using $\left\{h_m^l(\boldsymbol{x})\right\}_{m=1,\cdots N;l=1,\cdots,L}$ and $\left\{x_n^l(\boldsymbol{x})\right\}_{n=1,\cdots,N;l=1,\cdots,L}$ to denote the hidden-layer pre- and post-activations with random weights Θ_{random}. By chain rule, we have

$$\partial_{W^l_{random,ij}}x_m^L(\boldsymbol{x}) = \begin{cases}(N_{L-1})^{-1/2}\,\phi'(h_m^L(\boldsymbol{x}))\sum_n W_{random,mn}^L\frac{\partial x_n^{L-1}(\boldsymbol{x})}{\partial W^l_{random,ij}} & l\le L-1\\ (N_{L-1})^{-1/2}\,\phi'(h_m^L(\boldsymbol{x}))\delta_{im}x_j^{L-1}(\boldsymbol{x}) & l=L\end{cases} \quad [81]$$

  To the leading order

$$\begin{aligned}&\sum_{l=1}^{L}\langle\partial_{W^l_{random}}f(\Theta_{random},\boldsymbol{x})\cdot\partial_{W^l_{random}}f(\Theta_{random},\boldsymbol{x}')\rangle\\ =&\langle N_L^{-1}\boldsymbol{a}_{random}\cdot\boldsymbol{a}_{random}\rangle\cdot N_L^{-1}\sum_{l=1}^{L}\langle\sum_{m,ij}\left(\partial_{W^l_{random,ij}}x_m^L(\boldsymbol{x})\cdot\partial_{W^l_{random,ij}}x_m^L(\boldsymbol{x}')\right)\rangle\\ =&N_L^{-1}\sigma^2\sum_{l=1}^{L}\langle\sum_{m,ij}\left(\partial_{W^l_{random,ij}}x_m^L(\boldsymbol{x})\cdot\partial_{W^l_{random,ij}}x_m^L(\boldsymbol{x}')\right)\rangle\end{aligned} \quad [82]$$

and by plugging in SI Eq. 82 and keeping only the leading order terms

$$N_L^{-1}\sum_{l=1}^{L}\langle\sum_{m,ij}\left(\partial_{W^l_{random,ij}}x_m^L(\boldsymbol{x})\cdot\partial_{W^l_{random,ij}}x_m^L(\boldsymbol{x}')\right)\rangle$$

$$=\sigma^2N_L^{-1}\langle\left(\phi'(h_m^L(\boldsymbol{x}))\cdot\phi'(h_m^L(\boldsymbol{x}'))\right)\rangle N_{L-1}^{-1}\sum_{l=1}^{L-1}\langle\sum_{n,ij}\left(\partial_{W^l_{random,ij}}x_n^{L-1}(\boldsymbol{x})\cdot\partial_{W^l_{random,ij}}x_n^{L-1}(\boldsymbol{x}')\right)\rangle$$

$$+N_L^{-1}\langle\left(\phi'(h_m^L(\boldsymbol{x}))\cdot\phi'(h_m^L(\boldsymbol{x}'))\right)\rangle N_{L-1}^{-1}\sum_j x_j^{L-1}(\boldsymbol{x})\cdot x_j^{L-1}(\boldsymbol{x}')$$

$$=\dot{K}^L(\boldsymbol{x},\boldsymbol{x}')\left(\sigma^2N_{L-1}^{-1}\sum_{l=1}^{L-1}\langle\sum_{n,ij}\left(\partial_{W^l_{random,ij}}x_n^{L-1}(\boldsymbol{x})\cdot\partial_{W^l_{random,ij}}x_n^{L-1}(\boldsymbol{x}')\right)\rangle+K_{GP}^{L-1}(\boldsymbol{x},\boldsymbol{x}')\right) \quad [83]$$

Denote

$$\mathcal{Q}^{L-1}(\boldsymbol{x},\boldsymbol{x}')=\sigma^2N_{L-1}^{-1}\sum_{l=1}^{L-1}\langle\sum_{n,ij}\left(\partial_{W^l_{random,ij}}x_n^{L-1}(\boldsymbol{x})\cdot\partial_{W^l_{random,ij}}x_n^{L-1}(\boldsymbol{x}')\right)\rangle+K_{GP}^{L-1}(\boldsymbol{x},\boldsymbol{x}') \quad [84]$$

and we have

$$\mathcal{Q}^L(\boldsymbol{x},\boldsymbol{x}')=\sigma^2\dot{K}^L(\boldsymbol{x},\boldsymbol{x}')\mathcal{Q}^{L-1}(\boldsymbol{x},\boldsymbol{x}')+K_{GP}^L(\boldsymbol{x},\boldsymbol{x}') \quad [85]$$

with initial condition

$$\mathcal{Q}^{L=0}(\boldsymbol{x},\boldsymbol{x}')=N_0^{-1}\boldsymbol{x}\cdot\boldsymbol{x}' \quad [86]$$

Therefore $\mathcal{Q}^L$ obeys the same iterative relation and initial condition as $\tilde{K}^L\lambda$. So we have

$$\mathcal{Q}^L(\boldsymbol{x},\boldsymbol{x}')=\tilde{K}^L(\boldsymbol{x},\boldsymbol{x}')\lambda \quad [87]$$

We also have

$$\sum_{l=1}^{L}\langle\partial_{W^l_{random}}f(\Theta_{random},\boldsymbol{x})\cdot\partial_{W^l_{random}}f(\Theta_{random},\boldsymbol{x}')\rangle=\mathcal{Q}^L(\boldsymbol{x},\boldsymbol{x}')-K_{GP}^L(\boldsymbol{x},\boldsymbol{x}') \quad [88]$$

Combining the two contributions above we have

$$\langle\partial_{\Theta_{random}}f(\Theta_{random},\boldsymbol{x})\cdot\partial_{\Theta_{random}}f(\Theta_{random},\boldsymbol{x}')\rangle=\mathcal{Q}^L(\boldsymbol{x},\boldsymbol{x}')=\lambda\tilde{K}^L(\boldsymbol{x},\boldsymbol{x}') \quad [89]$$

The relation is similar to what has been shown in (5), the relevant scales of temperature β^{-1}, λ and time t are different.Thus $\tilde{K}^L(\boldsymbol{x},\boldsymbol{x}')$ is time-independent, and is equivalent to the NTK.

4. Multi-Head Theory

A. Summary of Main Theoretical Results. In multi-head CL, we consider both the mean and variance of the network's input-output mappings, for $T=2$ and $L=1$. The variance is not negligible as in single-head CL, as it causes the divergence of $G_{2,2}$ in the overfitting regime. Since we take L to be 1, in this section the L superscript of the kernels is neglected. Analogous to the kernel $\tilde{K}^L_{t,t'}(\boldsymbol{x},\boldsymbol{x}')$ defined in SI Eq. 5, we introduce a new "renormalized" kernel for multi-head CL, using the same notation

$$\tilde{K}_{2,2}(\boldsymbol{x},\boldsymbol{x}')\equiv u^1_{2,2}K^1_{2,2}(\boldsymbol{x},\boldsymbol{x}')-u^0_{2,2}K^0_{2,2}(\boldsymbol{x},\boldsymbol{x}') \quad [90]$$

where the "renormalization factors" $u^1_{2,2}$ and $u^0_{2,2}$ can be solved self-consistently as detailed in SI 4.B.3. Similarly as in SI 2, we introduce $\tilde{\boldsymbol{k}}_{2,2}(\boldsymbol{x})\in\mathbb{R}^P$, $\tilde{\boldsymbol{K}}_{2,2}\in\mathbb{R}^{P\times P}$ and $\tilde{k}_{2,2}\in\mathbb{R}$ for this kernel function applied on the training and testing data. The expressions for the statistics of the network's input-output mappings are given below.

A.1. The Mean Input-Output Mappings. The mean input-output mappings are given by

$$\langle f_2^1(\boldsymbol{x})\rangle=u^1_{1,2}\Delta\boldsymbol{k}_{2,2}(\boldsymbol{x})^\top\tilde{\boldsymbol{K}}_{2,2}^{-1}\left(\boldsymbol{Y}_2-u^1_{1,2}\left(u^1_{1,1}\right)^{-1}\boldsymbol{K}^1_{2,1}\left(\boldsymbol{K}^1_{1,1}\right)^{-1}\boldsymbol{Y}_1\right)+\boldsymbol{k}^1_{2,1}(\boldsymbol{x})^\top\left(\boldsymbol{K}^1_{1,1}\right)^{-1}\boldsymbol{Y}_1 \quad [91]$$

$$\langle f_2^2(\boldsymbol{x})\rangle=\tilde{\boldsymbol{k}}_{2,2}(\boldsymbol{x})^\top\tilde{\boldsymbol{K}}_{2,2}^{-1}\left(\boldsymbol{Y}_2-u^1_{1,2}\left(u^1_{1,1}\right)^{-1}\boldsymbol{K}^1_{2,1}\left(\boldsymbol{K}^1_{1,1}\right)^{-1}\boldsymbol{Y}_1\right)+u^1_{2,1}\left(u^1_{1,1}\right)^{-1}\boldsymbol{k}^1_{2,1}(\boldsymbol{x})^\top\left(\boldsymbol{K}^1_{1,1}\right)^{-1}\boldsymbol{Y}_1 \quad [92]$$

A.2. The Variance of the Input-Output Mappings. The variance of the input-output mappings are

$$\langle \delta f_2^1(\boldsymbol{x})^2\rangle = u_{1,1}^1 k_{2,2}^1(\boldsymbol{x},\boldsymbol{x}) - \left(u_{1,2}^1\right)^2 \Delta\boldsymbol{k}_{2,2}(\boldsymbol{x})^\top \tilde{\boldsymbol{K}}_{2,2}^{-1}\Delta\boldsymbol{k}_{2,2}(\boldsymbol{x}) - \left(u_{1,1}^1\boldsymbol{k}_{2,1}^1(\boldsymbol{x}) - u_{1,2}^1\Delta\boldsymbol{k}_{2,2}(\boldsymbol{x})\tilde{\boldsymbol{K}}_{2,2}^{-1}\tilde{\boldsymbol{K}}_{2,1}\right)\tilde{\boldsymbol{K}}_{1,1}^{-1}$$
$$\left(u_{1,1}^1\boldsymbol{k}_{2,1}^1(\boldsymbol{x}) - u_{1,2}^1\Delta\boldsymbol{k}_{2,2}(\boldsymbol{x})\tilde{\boldsymbol{K}}_{2,2}^{-1}\tilde{\boldsymbol{K}}_{2,1}\right)^\top - 2\left(u_{1,2}\right)^2\Delta\boldsymbol{k}_{2,2}(\boldsymbol{x})\tilde{\boldsymbol{K}}_{2,2}^{-1}\boldsymbol{k}_{2,2}^0(\boldsymbol{x})^\top$$
$$+ \left(u_{1,2}^1\right)^2 u_{2,2}^0\Delta\boldsymbol{k}_{2,2}(\boldsymbol{x})^\top\tilde{\boldsymbol{K}}_{2,2}^{-1}\boldsymbol{K}_{2,2}^0\tilde{\boldsymbol{K}}_{2,2}^{-1}\Delta\boldsymbol{k}_{2,2}(\boldsymbol{x}) \quad [93]$$

$$\langle \delta f_2^2(\boldsymbol{x})^2\rangle = u_{2,2}^1 k_{2,2}^1(\boldsymbol{x},\boldsymbol{x}) - \left(u_{2,2}^1\right)^2 \Delta\boldsymbol{k}_{2,2}(\boldsymbol{x})^\top \tilde{\boldsymbol{K}}_{2,2}^{-1}\Delta\boldsymbol{k}_{2,2}(\boldsymbol{x}) - \left(\tilde{\boldsymbol{k}}_{2,1}(\boldsymbol{x}) - \tilde{\boldsymbol{k}}_{2,2}(\boldsymbol{x})\tilde{\boldsymbol{K}}_{2,2}^{-1}\tilde{\boldsymbol{K}}_{2,1}\right)\tilde{\boldsymbol{K}}_{1,1}^{-1}$$
$$\left(\tilde{\boldsymbol{k}}_{2,1}(\boldsymbol{x}) - \tilde{\boldsymbol{k}}_{2,2}(\boldsymbol{x})\tilde{\boldsymbol{K}}_{2,2}^{-1}\tilde{\boldsymbol{K}}_{2,1}\right)^\top - 2u_{2,2}^0\tilde{\boldsymbol{k}}_{2,2}(\boldsymbol{x})\tilde{\boldsymbol{K}}_{2,2}^{-1}\boldsymbol{k}_{2,2}^0(\boldsymbol{x})^\top$$
$$+ u_{2,2}^0\tilde{\boldsymbol{k}}_{2,2}(\boldsymbol{x})\tilde{\boldsymbol{K}}_{2,2}^{-1}\boldsymbol{K}_{2,2}^0\tilde{\boldsymbol{K}}_{2,2}^{-1}\tilde{\boldsymbol{k}}_{2,2}(\boldsymbol{x})^\top. \quad [94]$$

These results hold for arbitrary λ. The "renormalization factors" $\left\{u_{1,1}^1, u_{1,2}^1, u_{2,2}^1, u_{2,2}^0\right\}$ are solved with SI Eqs. 129-132 in SI B.3. Plugging them back into Eqs. 91-94 allows us to evaluate $F_{2,1} = \left\langle\mathcal{L}(f_2^1, D_1)\right\rangle$, $G_{2,2} = \left\langle\mathcal{L}(f_2^2, D_2^{test})\right\rangle$ and $G_{2,1} = \left\langle\mathcal{L}(f_2^1, D_1^{test})\right\rangle$, which we showed in our multi-head results.

Furthermore, in the $\lambda \to \infty$ limit, the phase-transition boundary between the overfitting regime and the generalization regime and the corresponding α_c (shown in Figs. 5, 6) is calculated by solving

$$\frac{\|\boldsymbol{Y}_1\|^2}{\alpha}\boldsymbol{V}_1^\top\boldsymbol{P}_2\boldsymbol{V}_1 - \left(\frac{\|\boldsymbol{Y}_1\|\|\boldsymbol{Y}_2\|}{\alpha}\boldsymbol{V}_1^\top\boldsymbol{V}_2\right)^2\cdot\left(\frac{\|\boldsymbol{Y}_1\|^2}{\alpha}\|\boldsymbol{V}_1\|^2\right) = u_{1,1}^1\left(\gamma_{\text{feature}} - \alpha^{-1/2}\right) \quad [95]$$

$$\sigma^{-2}\left(u_{1,1}^1\right)^2 - (1-\alpha)u_{1,1}^1 - \|\boldsymbol{Y}_1\|^2\|\boldsymbol{V}_1\|^2 = 0 \quad [96]$$

These set of equations are polynomial equations in α, and can't be analytically solved. However, if we further assume that $\sigma^2 = 1$, and the data of each task is properly normalized such that $\frac{1}{P}\boldsymbol{Y}_1^\top\boldsymbol{K}_{1,1}^1\boldsymbol{Y}_1 = 1$, thus $\|\boldsymbol{V}_1\|^2 \equiv \frac{1}{N}\boldsymbol{Y}_1^\top\boldsymbol{K}_{1,1}^1\boldsymbol{Y}_1/\|\boldsymbol{Y}_1\|^2 = \frac{\alpha}{\|\mathbf{Y}_1\|^2}$, and similarly for $\boldsymbol{V}_2$. We can simplify SI Eqs. 95, 96 to

$$-\frac{\boldsymbol{V}_1^\top\boldsymbol{P}_2\boldsymbol{V}_1}{\|\boldsymbol{V}_1\|^2} + \cos(\boldsymbol{V}_1,\boldsymbol{V}_2)^2 + \gamma_{\text{feature}} = \alpha^{-1/2} \quad [97]$$

as given by main text Eq. 11.

For details of different solutions of the renormalization factors in the three phases, and the derivations of the phase-transition boundary see SI 4.B.5. Theoretical results regarding the hidden representations (Fig. 7(c,f)) are shown in SI 4.B.6.

B. Detailed Derivation.

B.1. Kernel Renormalization Theory. In this section, we will present the detailed derivation for mapping statistics of multi-head CL, in the thermodynamic finite-width limit ($P\to\infty, N\to\infty$). We start from the MGF for multi-head CL, given by

$$\mathcal{M}(\{\ell_T^{task\ t}\}_{t=1,\cdots,T}) \equiv \left[Z\left(D_1\right)\prod_{t=2}^{T} Z\left(\Theta_{t-1}, D_t\right)\right]^{-1}\exp\left(-\beta E\left(\Theta_1|D_1\right) - \sum_{t=1}^{T}\beta E\left(\Theta_t|\Theta_{t-1}, D_t\right) + \sum_{t=1}^{T}\ell_t f_T^t(\mathbf{x})\right) \quad [98]$$

where

$$E\left(\Theta_t|\Theta_{t-1}, D_t\right) = \frac{1}{2}\sum_{\mu=1}^{P}\left(f_t^t\left(\boldsymbol{x}_t^\mu\right) - y_t^\mu\right)^2 + \frac{1}{2}\beta^{-1}\sigma^{-2}\left\|\Theta_t\right\|^2 + \frac{1}{2}\beta^{-1}\lambda\left\|\mathcal{W}_t - \mathcal{W}_{t-1}\right\|^2. \quad [99]$$

$$E\left(\Theta_1|D_1\right) = \frac{1}{2}\sum_{\mu=1}^{P}\left(f_1^1\left(\boldsymbol{x}_1^\mu\right) - y_1^\mu\right)^2 + \frac{1}{2}\beta^{-1}\sigma^{-2}\left\|\Theta_1\right\|^2 \quad [100]$$

and

$$Z\left(\Theta_{t-1}, D_t\right) \equiv \int d\Theta_t\exp(-\beta E\left(\Theta_t|\Theta_{t-1}, D_t\right)) \quad [101]$$

$$Z\left(D_1\right) \equiv \int d\Theta_1\exp(-\beta E\left(\Theta_1|D_1\right)) \quad [102]$$

Here we introduce fields ℓ_t coupled to each mapping $f_T^t(\mathbf{x})$ after learning the T-th task, the statistics of $f_T^t(\mathbf{x})$ can therefore be calculated by

$$\langle f_T^t(\mathbf{x})\rangle = \frac{\partial\mathcal{M}(\{\ell_t\}_{t=1,\cdots,T})}{\partial\ell_t}|_{\{\ell_t\}_{t=1,\cdots,T}=0} \quad [103]$$

$$\langle\delta^2 f_T^t(\mathbf{x})\rangle = \frac{\partial^2\mathcal{M}(\{\ell_t\}_{t=1,\cdots,T})}{\partial(\ell_t)^2}|_{\{\ell_t\}_{t=1,\cdots,T}=0} \quad [104]$$

Similarly as in single-head CL, we use the replica method for the denominator in SI Eq. 98, and introduce auxilliary integration variable $\{\boldsymbol{v}_t^\alpha\}_{t=2,\cdots,T;\alpha=1,\cdots n}, \boldsymbol{v}_1^n \in \mathbb{R}^P$ using the H-S transform, and arrive at

$$\begin{aligned}\mathcal{M}\left(\{\ell_T^{task\ t}\}_{t=1,\cdots,T}\right) = \lim_{n\to 0}\int\prod_{\alpha=1}^{n}\prod_{t=2}^{T} d\boldsymbol{v}_t^\alpha \int d\boldsymbol{v}_1^n \int \prod_{\alpha=1}^{n}\prod_{t=2}^{T} d\Theta_t^\alpha \int d\Theta_1^n \\ \exp\left[-i\sum_{t,\alpha}\sum_{\mu=1}^{P} v_t^{\alpha,\mu}\left(f_t^t\left(\Theta_t^\alpha, \boldsymbol{x}_t^\mu\right) - y_t^\mu\right) - \frac{1}{2}\sigma^{-2}\sum_{t,\alpha}\|\Theta_t^\alpha\|^2 - \frac{1}{2}\lambda\sum_{t=2}^{T}\sum_{\alpha=1}^{n}\|\mathcal{W}_t^\alpha - \mathcal{W}_{t-1}^n\|^2 \right. \\ \left. -\frac{1}{2}\beta^{-1}\sum_{t,\alpha}\boldsymbol{v}_t^{\alpha\top}\boldsymbol{v}_t^\alpha + \sum_{t=1}^{T}\ell_t f_T^t(\boldsymbol{a}_t^n, \mathcal{W}_T^n, \mathbf{x})\right]\end{aligned} \tag{105}$$

where we use $f_t^t\left(\Theta_t^\alpha, \boldsymbol{x}_t^\mu\right)$ to denote the mapping with the replicated Θ_t^α,$f_t^t(\Theta_t^\alpha, \boldsymbol{x}_t^\mu) \equiv \frac{1}{\sqrt{N}}\boldsymbol{a}_t^\alpha\Phi(\mathcal{W}_t^\alpha, \boldsymbol{x}_t^\mu)$; and we use $f_T^t(\boldsymbol{a}_t^n, \mathcal{W}_T^n, \mathbf{x})$ to denote the t-th mapping after learning all T tasks with physical parameters $\{\boldsymbol{a}_t^n, \mathcal{W}_T^n\}$, on an arbitrary test input $\mathbf{x}$.Similar to single-head CL, we define $\sum_{t,\alpha}(\cdot)_{t,\alpha} \equiv (\cdot)_{1,n} + \sum_{t=2}^{T}\sum_{\alpha=1}^{n}(\cdot)_{t,\alpha}$.

We then integrate the readout weights $\{\boldsymbol{a}_t^\alpha\}_{t=1,\cdots T;\alpha=1,\cdots n}$, and obtain in the $\beta\to\infty$ limit

$$\mathcal{M}\left(\{\ell_t\}_{t=1,\cdots,T}\right) = \lim_{n\to 0}\int\prod_{\alpha=1}^{n}\prod_{t=1}^{T} d\boldsymbol{v}_t^\alpha \exp\left(-\frac{1}{2}\beta^{-1}\sum_{t,\alpha}\boldsymbol{v}_t^{\alpha\top}\boldsymbol{v}_t^\alpha + i\sum_{t,\alpha}\boldsymbol{v}_t^{\alpha\top}\boldsymbol{Y}_t + G(\{\tilde{\boldsymbol{v}}_t^\alpha\}_{\alpha=1,\cdots,n;t=2,\cdots,T}, \tilde{\boldsymbol{v}}_1^n)\right) \tag{106}$$

where

$$G(\{\tilde{\boldsymbol{v}}_t^\alpha\}_{\alpha=1,\cdots,n;t=2,\cdots,T}, \tilde{\boldsymbol{v}}_1^n) \equiv \log\left[\int\prod_{\alpha=1}^{n}\prod_{t=1}^{T} d\mathcal{W}_t^\alpha \exp\left(S_0\left(\{\mathcal{W}^\alpha\}_{\alpha=1,\cdots,n}\right) - \frac{\sigma^2}{2}\sum_{t,\alpha}\tilde{\boldsymbol{v}}_t^{\alpha\top}\boldsymbol{M}_{t,t}^{\alpha\alpha}\tilde{\boldsymbol{v}}_t^\alpha\right)\right] \tag{107}$$

and $S_0(\{\mathcal{W}^\alpha\}_{\alpha=1,\cdots,n})$ and $\boldsymbol{M}$ are defined in the same way as in SI Eqs. 34, 35. The only difference is that only diagonal elements of $\boldsymbol{M}$ appear in G.For simplicity, here we denote $\tilde{\boldsymbol{v}}_t^\alpha \equiv [\boldsymbol{v}_t^\alpha, \delta_{\alpha n}\ell_t] \in \mathbb{R}^{P+1}$, absorbing the fields coupled to the mappings on arbitrary $\mathbf{x}$ into $\boldsymbol{v}_t^\alpha$. It is still highly nontrivial to integrate the hidden-layer weights and compute $G(\{\tilde{\boldsymbol{v}}_t^\alpha\}_{\alpha=1,\cdots,n;t=2,\cdots,T}, \tilde{\boldsymbol{v}}_1^n)$ in general.

Infinite-width Limit: In the infinite-width limit, the distribution of $\{\mathcal{W}^\alpha\}_{\alpha=1,\cdots,n}$ is dominated by the prior determined by $S_0(\{\mathcal{W}^\alpha\}_{\alpha=1,\cdots,n})$. Therefore, G can be calculated by integrating over Gaussian $\{\mathcal{W}^\alpha\}_{\alpha=1,\cdots,n}$, resulting in

$$G(\{\tilde{\boldsymbol{v}}_t^\alpha\}_{\alpha=1,\cdots,n;t=2,\cdots,T}, \tilde{\boldsymbol{v}}_1^n) = -\frac{\sigma^2}{2}\sum_{t,\alpha}\tilde{\boldsymbol{v}}_t^{\alpha\top}\langle\boldsymbol{M}_{t,t}^{\alpha\alpha}\rangle_{\mathcal{W}}\tilde{\boldsymbol{v}}_t^\alpha \tag{108}$$

Compared to SI Eq. 38, there is no coupling between different replica indices, and no coupling between different time indices, which allows us to get rid of the replica easily. Using SI Eqs. 41-44, we have, in the $\beta\to\infty$ limit,

$$\mathcal{M}\left(\{\ell_t\}_{t=1,\cdots,T}\right) = \int\prod_{t=1}^{T} d\boldsymbol{v}_t \exp\left(i\sum_{t=1}^{T}\boldsymbol{v}_t^\top\boldsymbol{Y}_t - \frac{\sigma^2}{2}\sum_{t=1}^{T}\boldsymbol{v}_t^\top\boldsymbol{K}_{t,t}^{L,1}\boldsymbol{v}_t - \frac{\sigma^2}{2}\left(\ell_t\right)^2 k_{T,T}^{L,1}(\boldsymbol{x},\boldsymbol{x}) - \sigma^2\sum_{t=1}^{T}\ell_t k_{T,t}^{L,1}(\boldsymbol{x})\boldsymbol{v}_t\right) \tag{109}$$

$$= \exp\left(-\frac{\sigma^2}{2}\left(\ell_t\right)^2 k_{T,T}^{L,1}(\boldsymbol{x},\boldsymbol{x}) - \frac{\sigma^{-2}}{2}\sum_{t=1}^{T}(\boldsymbol{Y}_t + \sigma^2 i\ell_t\boldsymbol{k}_{T,t}^{L,1}(\boldsymbol{x}))\left(\boldsymbol{K}_{t,t}^{L,1}\right)^{-1}(\boldsymbol{Y}_t + i\ell_t\sigma^2\boldsymbol{k}_{T,t}^{L,1}(\boldsymbol{x}))^\top\right) \tag{110}$$

The mapping statistics are then simply given by

$$\langle f_T^t(\boldsymbol{x})\rangle = \boldsymbol{k}_{T,t}^{L,1}(\boldsymbol{x})\left(\boldsymbol{K}_{t,t}^{L,1}\right)^{-1}\boldsymbol{Y}_t \tag{111}$$

and

$$\langle\delta f_T^t(\boldsymbol{x})^2\rangle = \sigma^2\left(k_{T,T}^{L,1}(\boldsymbol{x},\boldsymbol{x}) - \boldsymbol{k}_{T,t}^{L,1}(\boldsymbol{x})\left(\boldsymbol{K}_{t,t}^{L,1}\right)^{-1}\boldsymbol{k}_{T,t}^{L,1}(\boldsymbol{x})^\top\right) \tag{112}$$

Therefore, the infinite-width limit in multi-head CL is trivial. The mapping statistics are identical as learning a single task, where the readout weights are learned with hidden-layer weights $\mathcal{W}_t$. There is no coupling between different tasks induced by the learning of hidden-layer weights. As a result, we focus on the thermodynamic finite-width limit, where the hidden-layer weights become task-relevant, and induce interactions between different tasks during CL.

Thermodynamic Finite-Width Limit: We focus on the thermodynamic finite-Width limit, and use the kernel renormalization approach as in (6) to derive the mapping statistics in this regime for networks with a *single hidden-layer*. First, for $L = 1$, using $\boldsymbol{w}_t^\alpha$ to denote a single row of $W_t^{1,\alpha}$, we have

$$G(\{\tilde{\boldsymbol{v}}_t^\alpha\}_{\alpha=1,\cdots,n;t=2,\cdots,T}, \tilde{\boldsymbol{v}}_1^n) = N \log\left[\int \prod_{\alpha=1}^{n}\prod_{t=1}^{T} d\boldsymbol{w}_t^\alpha \exp\left(S_0(\boldsymbol{w}) - \frac{\sigma^2}{2}\sum_{t,\alpha}\tilde{\boldsymbol{v}}_t^{\alpha\top}\boldsymbol{M}_{t,t}^{\alpha\alpha,w}\tilde{\boldsymbol{v}}_t^\alpha\right)\right] \tag{113}$$

where $\boldsymbol{M}_{t,t}^{\alpha\alpha,w}$ is defined similarly as SI Eq. 35, but replacing the $P\times N$ matrix $\Phi(\mathcal{W}_t^\alpha, \mathbf{x}_t)$ and N dimensional vector $\Phi(\boldsymbol{w}_t^\alpha, \mathbf{x})$, and replacing the P dimensional vector $\Phi(\mathcal{W}_t^\alpha, \mathbf{x})$ with a scalar $\Phi(\boldsymbol{w}_t^\alpha, \mathbf{x})$, and scaled by N to keep the elements $\mathcal{O}(1)$. $S_0(\boldsymbol{w})$ is also defined similarly as in SI Eq. 34, by replacing $\{\mathcal{W}_t^\alpha\}_{t=1,\cdots,T;\alpha=1,\cdots,n}$ with the row vectors $\{\boldsymbol{w}_t^\alpha\}_{t=1,\cdots,T;\alpha=1,\cdots,n}$. Furthermore, we adopt the Gaussian approximation equivalent to (6), such that

$$z_t^\alpha = N^{-1/2}\sigma\boldsymbol{v}_t^{\alpha\top}\Phi(\boldsymbol{w}_t^\alpha, \mathbf{x}_t) + N^{-1/2}\sigma\delta_{\alpha n}\ell^{task\ t}\Phi(\boldsymbol{w}_T^\alpha, \mathbf{x}) \tag{114}$$

is Gaussian with $\langle z_t^\alpha\rangle = 0$ and $\langle z_t^\alpha z_{t'}^\beta\rangle = \sigma^2\frac{1}{N}\tilde{\boldsymbol{v}}_t^{\alpha\top}\langle\boldsymbol{M}_{t,t'}^{\alpha\beta,w}\rangle_w\tilde{\boldsymbol{v}}_{t'}^\beta$where the average $\langle\cdot\rangle_w$ is w.r.t. the prior Gaussian distribution in $\boldsymbol{w}$, whose probability density function is proportional to $\exp(-S_0(\boldsymbol{w}))$. Therefore, we replace the integral over $\boldsymbol{w}_t^\alpha$ with a Gaussian integral over z_t^α, and introducing $H_{t,t',\alpha,\beta} = \sigma^2\frac{1}{N}\tilde{\boldsymbol{v}}_t^{\alpha\top}\langle\boldsymbol{M}_{t,t'}^{\alpha\beta,w}\rangle_w\tilde{\boldsymbol{v}}_{t'}^\beta$, we have

$$\begin{aligned}
&G(\{\tilde{v}_t^\alpha\}_{\alpha=1,\cdots,n;t=1,\cdots,T}) = G(\boldsymbol{H})\\
&=N\log\left[\int\prod_{\alpha=1}^{n}\prod_{t=1}^{T}dz_t^\alpha\det(\boldsymbol{H})^{-1/2}\exp\left(-\frac{1}{2}\sum_{t,\alpha}\sum_{t',\beta}z_t^{\alpha\top}[\boldsymbol{H}^{-1}]_{t,t',\alpha\beta}z_{t'}^\beta - \frac{1}{2}\sum_{t,\alpha}(z_t^\alpha)^2\right)\right]\\
&=-\frac{N}{2}\log\det(\mathbb{I}+\boldsymbol{H})
\end{aligned} \tag{115}$$

Plugging into SI Eq. 106, and using the Fourier representation of the Dirac delta function to introduce $\boldsymbol{H}$,i.e.,

$$\delta(\sigma^{-2}H_{t,t',\alpha,\beta} - \frac{1}{N}\tilde{\boldsymbol{v}}_t^{\alpha\top}\langle\boldsymbol{M}_{t,t'}^{\alpha\beta,w}\rangle_w\tilde{\boldsymbol{v}}_{t'}^\beta) = \int d\boldsymbol{U}\exp(\sum_{t,t',\alpha,\beta}U_{t,t',\alpha,\beta}(\sigma^{-2}H_{t,t',\alpha,\beta} - \frac{1}{N}\tilde{\boldsymbol{v}}_t^{\alpha\top}\langle\boldsymbol{M}_{t,t'}^{\alpha\beta,w}\rangle_w\tilde{\boldsymbol{v}}_{t'}^\beta))$$

, we have

$$\begin{aligned}
\mathcal{M}\left(\{\ell_t\}_{t=1,\cdots,T}\right) = \lim_{n\to0}\int\prod_{\alpha=1}^{n}\prod_{t=1}^{T}d\boldsymbol{v}_t^\alpha\int d\boldsymbol{U}\int d\boldsymbol{H}\exp(-\frac{1}{2}\beta^{-1}\sum_{t,\alpha}\boldsymbol{v}_t^{\alpha\top}\boldsymbol{v}_t^\alpha + i\sum_{t,\alpha}\boldsymbol{v}_t^{\alpha\top}\boldsymbol{Y}_t - \frac{N}{2}\log\det(\mathbb{I}+\boldsymbol{H})\\
+\sigma^{-2}\frac{N}{2}\mathrm{Tr}(\boldsymbol{U}\boldsymbol{H}) - \frac{1}{2}\sum\sum_{t',\beta}U_{t,t',\alpha,\beta}\tilde{\boldsymbol{v}}_t^{\alpha\top}\langle\boldsymbol{M}_{t,t'}^{\alpha\beta,w}\rangle_w\tilde{\boldsymbol{v}}_{t'}^\beta)
\end{aligned} \tag{116}$$

In the limit $N\to\infty$, $P\to\infty$, $\alpha = P/N \sim \mathcal{O}(1)$. The exponent of $\mathcal{M}(\ell)$ scales with N, allowing us to adopt the saddle-point approximation for the integral over H and U. Taking derivative of the exponent w.r.t. H and set it to 0, we obtain $\sigma^{-2}\boldsymbol{U} = (\mathbb{I}+\boldsymbol{H})^{-1}$,plugging back in, we have

$$\begin{aligned}
\mathcal{M}\left(\{\ell_t\}_{t=1,\cdots,T}\right) = \lim_{n\to0}\int\prod_{\alpha=1}^{n}\prod_{t=1}^{T}d\boldsymbol{v}_t^\alpha\int d\boldsymbol{U}\exp\Bigg[-\frac{1}{2}\beta^{-1}\sum_{t,\alpha}\boldsymbol{v}_t^{\alpha\top}\boldsymbol{v}_t^\alpha + i\sum_{t,\alpha}\boldsymbol{v}_t^{\alpha\top}\boldsymbol{Y}_t + \frac{N}{2}\log\det(\boldsymbol{U})\\
-\sigma^{-2}\frac{N}{2}\mathrm{Tr}(\boldsymbol{U}) - \frac{1}{2}\sum_{t,\alpha}\sum_{t',\beta}U_{t,t',\alpha,\beta}\tilde{\boldsymbol{v}}_t^{\alpha\top}\langle\boldsymbol{M}_{t,t'}^{\alpha\beta,w}\rangle_w\tilde{\boldsymbol{v}}_{t'}^\beta\Bigg]
\end{aligned} \tag{117}$$

To proceed from SI Eq. 117, we take the replica symmetric ansatz, analogous to calculation for the Franz-Parisi potential(7), for $t, t' \geq 2$

$$U_{t,t',\alpha,\beta} = \begin{cases} u_{t,t'}^1 & \{\alpha=\beta, \tau=\tau'\}\cup\{\alpha=n,\tau<\tau'\}\cup\{\beta=n,\tau>\tau'\}\\ u_{t,t'}^0 & otherwise\end{cases} \tag{118}$$

otherwise denote

$$U_{t,1,\alpha,n} = u_{t,1}^1; u_{t,1}^0 \equiv 0 \tag{119}$$

Furthermore, $\langle\boldsymbol{M}_{t,t'}^{\alpha\beta,w}\rangle_w$ can be written in the same way as SI Eqs. 41-44, thus getting rid of the replica indices in $\boldsymbol{M}$. Analogous to SI Eq. 45, we introduce

$$\tilde{K}_{t,t'}(\boldsymbol{x},\boldsymbol{x}') = u_{t,t'}^1K_{t,t'}^{L,1}(\boldsymbol{x},\boldsymbol{x}') - u_{t,t'}^0K_{t,t'}^{L,0}(\boldsymbol{x},\boldsymbol{x}') \tag{120}$$

and correspondingly, $\tilde{\boldsymbol{K}}^{L}_{t,t'} = u^1_{t,t'}\boldsymbol{K}^{L,1}_{t,t'} - u^0_{t,t'}\boldsymbol{K}^{L,0}_{t,t'}$. $\tilde{\boldsymbol{k}}^{L}_{t,t'}(\boldsymbol{x}) = u^1_{t,t'}\boldsymbol{k}^{L,1}_{t,t'}(\boldsymbol{x}) - u^0_{t,t'}\boldsymbol{k}^{L,0}_{t,t'}(\boldsymbol{x})$.We also introduce $\Delta\boldsymbol{k}_{t,t'}(\boldsymbol{x}) = \boldsymbol{k}^{L,1}_{t,t'}(\boldsymbol{x}) - \boldsymbol{k}^{L,0}_{t,t'}(\boldsymbol{x})$. Note that for multi-head CL, the theoretical results are for $L=1$, we thus neglect the L index hereafter.

$\mathcal{M}(\ell)$ is quadratic in $\boldsymbol{v}^\alpha_t$, in principle, it allows us to perform integration over $\boldsymbol{v}^\alpha_t$ for arbitrary T in a similar way as in SI 3.??. By introducing $\boldsymbol{p}_t = \sum_{\alpha=1}^{n}\boldsymbol{v}^\alpha_t$ and integrating over $\{\boldsymbol{v}^\alpha_t\}_{\alpha=1,\cdots,n-1;t=1,\cdots,T}$, letting $\boldsymbol{v}_t \equiv \boldsymbol{v}^n_t$, we arrive at

$$
\begin{aligned}
\mathcal{M}\left(\{\ell_t\}_{t=1,\cdots,T}\right) = \int d\boldsymbol{U}\int\prod_{t=2}^{T} d\boldsymbol{p}_t\int\prod_{t=1}^{T} d\boldsymbol{v}_t \exp\Bigg[&\frac{N}{2}\log\det(\boldsymbol{U}) \\
&-\sigma^{-2}\frac{N}{2}\mathrm{Tr}(\boldsymbol{U}) - \frac{1}{2}\sum_{t=2}^{T}\boldsymbol{p}_t^\top(u^0_{t,t}\boldsymbol{K}^0_{t,t} - \tilde{\boldsymbol{K}}_{t,t})\boldsymbol{p}_t - \frac{1}{2}u^1_{1,1}\boldsymbol{v}_1^\top\boldsymbol{K}^1_{1,1}\boldsymbol{v}_1 \\
&-\sum_{t=t'}^{T}\sum_{t'=2}^{T}\boldsymbol{p}_t^\top\tilde{\boldsymbol{K}}_{t,t'}\boldsymbol{v}_{t'} - \sum_{t=t'+1}^{T}\sum_{t'=2}^{T}u^0_{t,t'}\boldsymbol{p}_t^\top\boldsymbol{K}^0_{t,t'}\boldsymbol{p}_{t'} - \sum_{t=2}^{T}u^1_{t,1}\boldsymbol{v}_1^\top\boldsymbol{K}^1_{1,t}\boldsymbol{p}_t + i\sum_{t=2}^{T}\boldsymbol{p}_t^\top\boldsymbol{Y}_t + i\boldsymbol{v}_1^\top\boldsymbol{Y}_1 \\
&-(n-2)\sum_{t=2}^{T}\log\det\tilde{\boldsymbol{K}}_{t,t} + \frac{n}{2}\sum_{t=2}^{T}\boldsymbol{p}_t^\top\tilde{\boldsymbol{K}}_{t,t}\boldsymbol{p}_t - n\sum_{t=2}^{T}\boldsymbol{p}_t^\top\tilde{\boldsymbol{K}}_{t,t}\boldsymbol{v}_t + \frac{n}{2}\sum_{t=1}^{T}\boldsymbol{v}_t^\top\tilde{\boldsymbol{K}}_{t,t}\boldsymbol{v}_t \\
&-\sum_{t=1}^{T}\sum_{t'=t+1}^{T}u^1_{t,t'}\ell_t\Delta\boldsymbol{k}_{T,t'}(\boldsymbol{x})\boldsymbol{v}_{t'} - \sum_{t=1}^{T}\sum_{t'=1}^{t}\ell_t[u^1_{t,t'}\boldsymbol{k}^1_{T,t'}(\boldsymbol{x}) - u^0_{t,t'}k^0_{T,t'}(\boldsymbol{x})]\boldsymbol{v}_{t'} \\
&-\sum_{t=1}^{T}\sum_{t'=t+1}^{T}u^1_{t,t'}\ell_t\boldsymbol{k}^0_{T,t'}(\boldsymbol{x})\boldsymbol{p}_{t'} - \sum_{t=1}^{T}\sum_{t'=2}^{t}u^0_{t,t'}\ell_t\boldsymbol{k}^0_{T,t'}(\boldsymbol{x})\boldsymbol{p}_{t'} - \sum_{t,t'=1}^{T}k^1_{T,T}(\boldsymbol{x},\boldsymbol{x})u^1_{t,t'}\ell_t\ell_{t'}\Bigg]
\end{aligned}
\quad [121]
$$

First, we note that the structure of SI Eq. 121 is quite similar to SI Eq. 49. Comparing the two equations, there are several major differences. One is that the factors $\left\{m^1_{t,t'}\right\}_{t,t'=1,\cdots,T}$ and $\left\{m^0_{t,t'}\right\}_{t,t'=1,\cdots,T}$ in SI Eq. 49 are determined by the prior distribution in the readout weights $\boldsymbol{a}_t$, the renormalization factors $\left\{u^1_{t,t'}\right\}_{t,t'=1,\cdots,T}$ and $\left\{u^0_{t,t'}\right\}_{t,t'=1,\cdots,T}$ incorporate the effect of learning the data, and need to be solved self-consistently. The other difference is in the terms containing the external field $\{\ell_t\}_{t=1,\cdots,T}$, while in single-head CL we only introduce one external field ℓ, in multi-head CL we introduce T external fields each coupled to one mapping. Furthermore, in multi-head CL, the mappings utilize $\boldsymbol{a}_t$ and $\mathcal{W}_T$, at different times, some kernels are renormalized by renormalization factors with different time indices. As an example, we see kernels with time indices t, T renormalized by renormalization factors with time indices t and t'. In single-head CL, however, kernels with time indices t and t' always appear with $m^1_{t,t'}$ or $m^0_{t,t'}$.Finally, we stress that unlike in SI Eq. 49 in SI 3.B.3, we keep also the $\mathcal{O}(n)$ terms, as we will see later, these terms will determine the self-consistent equations in the renormalization factors.

B.2. Statistics of the Input-Output Mappings for Arbitrary Number of Tasks T. The statistics of input-output mappings can be evaluated for arbitrary T, by taking derivative of $\mathcal{M}(\ell)$ w.r.t. ℓ_t. Using SI Eqs. 103, 104, we have

Mean: The mean mappings are given by

$$
\langle f^t_T(\boldsymbol{x})\rangle = \sum_{t'=t+1}^{T}u^1_{t,t'}\Delta\boldsymbol{k}_{T,t'}(\boldsymbol{x})^\top\langle -i\boldsymbol{v}_{t'}\rangle + \sum_{t'=1}^{t}[u^1_{t,t'}\boldsymbol{k}^{L,1}_{T,t'}(\boldsymbol{x}) - u^0_{t,t'}k^{L,0}_{T,t'}(\boldsymbol{x})]^\top\langle -i\boldsymbol{v}_{t'}\rangle \quad [122]
$$

Variance:

$$
\begin{aligned}
\langle \delta^2 f_T^t(\boldsymbol{x})\rangle = {} & u_{t,t}^1 k_{T,T}^1(\boldsymbol{x},\boldsymbol{x}) - \sum_{\tau,\tau'=t+1}^{T} \Delta \boldsymbol{k}_{T,\tau}(\boldsymbol{x}) u_{t,\tau}^1 u_{t,\tau'} \langle \delta\boldsymbol{v}_\tau \delta\boldsymbol{v}_{\tau'}^\top\rangle \Delta\boldsymbol{k}_{T,\tau'}(\boldsymbol{x})^\top \\
& - \sum_{\tau,\tau'=1}^{t} [u_{t,\tau}^1 \boldsymbol{k}_{T,\tau}^1(\boldsymbol{x}) - u_{t,\tau}^0 \boldsymbol{k}_{T,\tau}^0(\boldsymbol{x})] \langle \delta\boldsymbol{v}_\tau \delta\boldsymbol{v}_{\tau'}^\top\rangle [u_{t,\tau'}^1 \boldsymbol{k}_{T,\tau'}^1(\boldsymbol{x}) - u_{t,\tau'}^0 \boldsymbol{k}_{T,\tau'}^0(\boldsymbol{x})]^\top \\
& - 2\sum_{\tau=1}^{t} \sum_{\tau'=t+1}^{T} u_{t,\tau'}^1 [u_{t,\tau}^1 \boldsymbol{k}_{T,\tau}^1(\boldsymbol{x}) - u_{t,\tau}^0 \boldsymbol{k}_{T,\tau}^0(\boldsymbol{x})] \langle \delta\boldsymbol{v}_\tau \delta\boldsymbol{v}_{\tau'}^\top\rangle \Delta\boldsymbol{k}_{T,\tau'}(\boldsymbol{x})^\top \\
& - 2\sum_{\tau=t+1}^{T} \sum_{\tau'=t+1}^{\tau} u_{t,\tau}^1 u_{t,\tau'}^1 \Delta\boldsymbol{k}_{T,\tau}(\boldsymbol{x}) \langle \delta\boldsymbol{v}_\tau \delta\boldsymbol{p}_{\tau'}^\top\rangle \boldsymbol{k}_{T,\tau'}^0(\boldsymbol{x})^\top \\
& - 2\sum_{\tau=t+1}^{T} \sum_{\tau'=2}^{t} u_{t,\tau}^1 u_{t,\tau'}^0 \Delta\boldsymbol{k}_{T,\tau}(\boldsymbol{x}) \langle \delta\boldsymbol{v}_\tau \delta\boldsymbol{p}_{\tau'}^\top\rangle \boldsymbol{k}_{T,\tau'}^0(\boldsymbol{x})^\top \\
& - 2\sum_{\tau=1}^{t} \sum_{\tau'=1}^{\tau} u_{t,\tau'}^0 [u_{t,\tau}^1 \boldsymbol{k}_{T,\tau}^1(\boldsymbol{x}) - u_{t,\tau}^0 k_{T,\tau}^0(\boldsymbol{x})] \langle \delta\boldsymbol{v}_\tau \delta\boldsymbol{p}_{\tau'}^\top\rangle \boldsymbol{k}_{T,\tau'}^0(\boldsymbol{x})^\top
\end{aligned}
\qquad [123]
$$

The first and second moments of $\{\boldsymbol{v}_t, \boldsymbol{p}_t\}_{t=2}^T$ and $\boldsymbol{v}_1$ are the same as in SI 4.B.3, given by SI Eqs. 51, 54, 55, but replacing the factors $\left\{m_{t,t'}^1\right\}_{t,t'=1,\cdots,T}$, $\left\{m_{t,t'}^0\right\}_{t,t'=1,\cdots,T}$ with $\left\{u_{t,t'}^1\right\}_{t,t'=1,\cdots,T}$ and $\left\{u_{t,t'}^0\right\}_{t,t'=1,\cdots,T}$. Taking $T=2$ and $t=1,2$, we can obtain the results presented in SI 4.A.

To complete the calculation, we need to compute the renormalization factors $\left\{u_{t,t'}^1\right\}_{t,t'=1,\cdots,T}$ and $\left\{u_{t,t'}^0\right\}_{t,t'=1,\cdots,T}$. They obey self-consistent equations derived from saddle-point approximation of the integrals in SI Eq. 121 w.r.t. $\boldsymbol{U}$. The self-consistent equations for general T are complicated for the following reasons. First, using the replica symmetry ansatz, explicitly writing down $\log\det \boldsymbol{U}$ as a function of $\left\{u_{t,t'}^1\right\}_{t,t'=1,\cdots,T}$ and $\left\{u_{t,t'}^0\right\}_{t,t'=1,\cdots,T}$ for arbitrary T is complicated. Second, the total number of renormalization factors grow quadratically with the number of tasks. For T tasks, there are a total of T^2 renormalization factors. In the scope of this paper, we will only present the self-consistent equations for up to $T=3$ in SI A.B.3, which we use in our main results.

B.3. Self-Consistent Equations for the Renormalization Factors Up to $T=3$. In this section, we start from SI Eq. 121 and present the detailed derivation of the self-consistent equations for the renormalization factors for $T=2$. For $T=3$, we present the final result of the effective Hamiltonian used to derive the self-consistent equations on the renormalization factors, the detailed derivation is similar as for $T=2$.

Self-Consistent Equations for $T=2$ **:** The terms coupled to the external field $\{\ell_t\}_{t=1,\cdots,T}$ do not impact the self-consistent equation, and we may neglect them for simplicity, and for $T=2$, SI Eq. 121 simplifies to

$$
\begin{aligned}
\mathcal{M}\left(\{\ell_t\}_{t=1,2}=0\right) = \int d\boldsymbol{U} \int d\boldsymbol{p}_2 \int \prod_{t=1}^{2} d\boldsymbol{v}_t \exp\Bigg[& \frac{N}{2}\log\det(\boldsymbol{U}) - \sigma^{-2}\frac{N}{2}\mathrm{Tr}(\boldsymbol{U}) - \frac{1}{2}\boldsymbol{p}_2^\top (u_{2,2}^0 \boldsymbol{K}_{2,2}^0 - \tilde{\boldsymbol{K}}_{2,2})\boldsymbol{p}_2 - \frac{1}{2}\boldsymbol{v}_1^\top \tilde{\boldsymbol{K}}_{1,1}\boldsymbol{v}_1 \\
& - \boldsymbol{p}_2^\top \tilde{\boldsymbol{K}}_{2,2}\boldsymbol{v}_2 - \boldsymbol{v}_1^\top \tilde{\boldsymbol{K}}_{1,2}\boldsymbol{p}_2 + i\boldsymbol{p}_2^\top \boldsymbol{Y}_2 + i\boldsymbol{v}_1^\top \boldsymbol{Y}_1 - \frac{1}{2}(n-2)\log\det\tilde{\boldsymbol{K}}_{2,2} \\
& + \frac{n}{2}\boldsymbol{p}_2^\top \tilde{\boldsymbol{K}}_{2,2}\boldsymbol{p}_2 - n\boldsymbol{p}_2^\top \tilde{\boldsymbol{K}}_{2,2}\boldsymbol{v}_2 + \frac{n}{2}\sum_{t=1}^{2}\boldsymbol{v}_t^\top \tilde{\boldsymbol{K}}_{t,t}\boldsymbol{v}_t \Bigg]
\end{aligned}
\qquad [124]
$$

First, note that for $T=2$, we can derive explicit form of $\log\det(\boldsymbol{U})$ in terms of $\left\{u_{t,t'}^1\right\}_{t,t'=1,\cdots,T}$ and $\left\{u_{t,t'}^0\right\}_{t,t'=1,\cdots,T}$, as

$$
\log\det(\boldsymbol{U}) = \log u_{1,1}^1 + n\log\left(u_{2,2}^1 - u_{2,2}^0\right) + n\left(u_{2,2}^1 - u_{2,2}^0\right)^{-1}\left(u_{2,2}^0 - (u_{1,2}^1)^2 (u_{1,1}^1)^{-1}\right) \qquad [125]
$$

Plugging in $\log\det(\boldsymbol{U})$ and $\mathrm{Tr}(\boldsymbol{U})$, and integrating over $\boldsymbol{p}_2$ and $\boldsymbol{v}_2$, we have

$$
\begin{aligned}
&\mathcal{M}\left(\{\ell_t\}_{t=1,2}=0\right)\\
&=\int du^1_{1,1}\int du^1_{1,2}\int du^1_{2,2}\int du^0_{2,2}\int d\boldsymbol{v}_1\\
&\quad \exp\Bigg[\frac{N}{2}\log u^1_{1,1}+\frac{N}{2}n\log\left(u^1_{2,2}-u^0_{2,2}\right)+\frac{N}{2}n\left(u^1_{2,2}-u^0_{2,2}\right)^{-1}\left(u^0_{2,2}-(u^1_{1,2})^2(u^1_{1,1})^{-1}\right)-\sigma^{-2}\frac{N}{2}u^1_{1,1}-\sigma^{-2}n\frac{N}{2}u^1_{2,2}\\
&\quad +i\boldsymbol{v}_1^\top\boldsymbol{Y}_1-\frac{1}{2}\boldsymbol{v}_1^\top\tilde{\boldsymbol{K}}_{1,1}\boldsymbol{v}_1-\frac{n}{2}\log\det\tilde{\boldsymbol{K}}_{2,2}-\frac{n}{2}u^0_{2,2}\mathrm{Tr}(\left(\tilde{\boldsymbol{K}}_{2,2}\right)^{-1}\boldsymbol{K}^0_{2,2})\\
&\quad +\frac{N}{2}n\left(i\boldsymbol{Y}_2+\tilde{\boldsymbol{K}}_{2,1}\boldsymbol{v}_1\right)^\top\left(\tilde{\boldsymbol{K}}_{2,2}\right)^{-1}\left(i\boldsymbol{Y}_2+\tilde{\boldsymbol{K}}_{2,1}\boldsymbol{v}_1\right)\Bigg]
\end{aligned}
\tag{126}
$$

Further integrating $\boldsymbol{v}_1$, we have

$$
\mathcal{M}(\{\ell_t\}_{t=1,2}=0)=\int du^1_{1,1}\int du^1_{1,2}\int du^1_{2,2}\int du^0_{2,2}\exp(-\frac{N}{2}\mathcal{H}_{\mathrm{eff}}(u^1_{1,1},u^1_{1,2},u^1_{2,2},u^0_{2,2}))
\tag{127}
$$

where

$$
\begin{aligned}
\mathcal{H}_{\mathrm{eff}}(u^1_{1,1},u^1_{1,2},u^1_{2,2},u^0_{2,2})&=\sigma^{-2}u^1_{1,1}-(1-\alpha)\log u^1_{1,1}+\frac{1}{N}\boldsymbol{Y}_1^\top\tilde{\boldsymbol{K}}^{-1}_{1,1}\boldsymbol{Y}_1+\log\det\boldsymbol{K}^1_{1,1}\\
&-n\log\left(u^1_{2,2}-u^0_{2,2}\right)-n\left(u^1_{2,2}-u^0_{2,2}\right)^{-1}\left(u^0_{2,2}-(u^1_{1,2})^2(u^1_{1,1})^{-1}\right)+\sigma^{-2}nu^1_{2,2}\\
&+n\frac{1}{N}\log\det\tilde{\boldsymbol{K}}_{2,2}+nu^0_{2,2}\frac{1}{N}\mathrm{Tr}(\tilde{\boldsymbol{K}}^{-1}_{2,2}\boldsymbol{K}^0_{2,2})-n\frac{1}{N}\mathrm{Tr}(\tilde{\boldsymbol{K}}_{2,1}\tilde{\boldsymbol{K}}^{-1}_{1,1}\tilde{\boldsymbol{K}}^\top_{2,1}\tilde{\boldsymbol{K}}^{-1}_{2,2})\\
&+n\frac{1}{N}\left(\boldsymbol{Y}_2-\tilde{\boldsymbol{K}}_{2,1}\tilde{\boldsymbol{K}}^{-1}_{1,1}\boldsymbol{Y}_1\right)^\top\tilde{\boldsymbol{K}}^{-1}_{2,2}\left(\boldsymbol{Y}_2-\tilde{\boldsymbol{K}}_{2,1}\tilde{\boldsymbol{K}}^{-1}_{1,1}\boldsymbol{Y}_1\right)
\end{aligned}
\tag{128}
$$

We see that the leading terms in $u^1_{1,1}$ are $\mathcal{O}(1)$, while the leading terms in $u^1_{2,2}$,$u^0_{2,2}$ and $u^1_{1,2}$ are all $\mathcal{O}(n)$, this indicates that the solution of $u^1_{1,1}$ will not be affected by the other renormalization factors, reflecting the sequential nature of the learning.The self-consistent equations for these renormalization factors are derived by taking derivative of SI Eq. 128 w.r.t. them and setting to 0, resulting in

$$
\sigma^{-2}\left(u^1_{1,1}\right)^2-(1-\alpha)u^1_{1,1}-\frac{1}{N}\boldsymbol{Y}_1^\top\left(\boldsymbol{K}^1_{1,1}\right)^{-1}\boldsymbol{Y}_1=0
\tag{129}
$$

$$
u^1_{1,2}\left(u^1_{1,1}(u^1_{2,2}-u^0_{2,2})\right)^{-1}+\frac{1}{N}\mathrm{Tr}\left(\tilde{\boldsymbol{K}}_{2,1}\tilde{\boldsymbol{K}}^{-1}_{1,1}\boldsymbol{K}^1_{1,2}\tilde{\boldsymbol{K}}^{-1}_{2,2}\right)+\frac{1}{N}\boldsymbol{Y}_1^\top\tilde{\boldsymbol{K}}^{-1}_{1,1}\boldsymbol{K}^1_{1,2}\tilde{\boldsymbol{K}}^{-1}_{2,2}\left(\boldsymbol{Y}_2-\tilde{\boldsymbol{K}}_{2,1}\tilde{\boldsymbol{K}}^{-1}_{1,1}\boldsymbol{Y}_1\right)=0
\tag{130}
$$

$$
\begin{aligned}
&\left(u^1_{2,2}-u^0_{2,2}\right)^{-1}-\sigma^{-2}+u^0_{2,2}\frac{1}{N}\mathrm{Tr}\left(\tilde{\boldsymbol{K}}^{-1}_{2,2}\Delta\boldsymbol{K}_{2,2}\tilde{\boldsymbol{K}}^{-1}_{2,2}\boldsymbol{K}^0_{2,2}\right)-\frac{1}{N}\mathrm{Tr}\left(\tilde{\boldsymbol{K}}^{-1}_{2,2}\boldsymbol{K}^1_{2,2}\right)\\
&-\frac{1}{N}\mathrm{Tr}\left(\tilde{\boldsymbol{K}}_{2,1}\tilde{\boldsymbol{K}}^{-1}_{1,1}\tilde{\boldsymbol{K}}^\top_{2,1}\tilde{\boldsymbol{K}}^{-1}_{2,2}\Delta\boldsymbol{K}_{2,2}\tilde{\boldsymbol{K}}^{-1}_{2,2}\right)+\frac{1}{N}\left(\boldsymbol{Y}_2-\tilde{\boldsymbol{K}}_{2,1}\tilde{\boldsymbol{K}}^{-1}_{1,1}\boldsymbol{Y}_1\right)^\top\tilde{\boldsymbol{K}}^{-1}_{2,2}\Delta\boldsymbol{K}_{2,2}\tilde{\boldsymbol{K}}^{-1}_{2,2}\left(\boldsymbol{Y}_2-\tilde{\boldsymbol{K}}_{2,1}\tilde{\boldsymbol{K}}^{-1}_{1,1}\boldsymbol{Y}_1\right)=0
\end{aligned}
\tag{131}
$$

$$
\begin{aligned}
&\left(u^0_{2,2}-\left(u^1_{1,2}\right)^2\left(u^1_{1,1}\right)^{-1}\right)\left(u^1_{2,2}-u^0_{2,2}\right)^{-2}-u^0_{2,2}\frac{1}{N}\mathrm{Tr}\left(\tilde{\boldsymbol{K}}^{-1}_{2,2}\boldsymbol{K}^0_{2,2}\tilde{\boldsymbol{K}}^{-1}_{2,2}\boldsymbol{K}^0_{2,2}\right)\\
&+\frac{1}{N}\mathrm{Tr}\left(\tilde{\boldsymbol{K}}_{2,1}\tilde{\boldsymbol{K}}^{-1}_{1,1}\tilde{\boldsymbol{K}}^\top_{2,1}\tilde{\boldsymbol{K}}^{-1}_{2,2}\boldsymbol{K}^0_{2,2}\tilde{\boldsymbol{K}}^{-1}_{2,2}\right)-\frac{1}{N}\left(\boldsymbol{Y}_2-\tilde{\boldsymbol{K}}_{2,1}\tilde{\boldsymbol{K}}^{-1}_{1,1}\boldsymbol{Y}_1\right)^\top\tilde{\boldsymbol{K}}^{-1}_{2,2}\boldsymbol{K}^0_{2,2}\tilde{\boldsymbol{K}}^{-1}_{2,2}\left(\boldsymbol{Y}_2-\tilde{\boldsymbol{K}}_{2,1}\tilde{\boldsymbol{K}}^{-1}_{1,1}\boldsymbol{Y}_1\right)=0
\end{aligned}
\tag{132}
$$

Effective Hamiltonian for $T=3$: The effective Hamiltonian for $T=3$ can be derived similarly as for $T=2$. For $T=3$, we have 9 renormalization factors, among which $u^1_{1,1},u^1_{1,2},u^1_{2,2}$ and $u^0_{2,2}$ satisfy SI Eqs. 129-130. The other renormalization factors including $u^1_{1,3},u^1_{2,3},u^0_{2,3},u^1_{3,3}$ and $u^0_{3,3}$ can be solved by taking derivative of the following effective Hamiltonian (SI Eq. 133) w.r.t. each of them and setting to 0.

$$
\begin{aligned}
&\mathcal{H}_{\mathrm{eff}}(u^1_{1,3},u^1_{2,3},u^0_{2,3},u^1_{3,3},u^0_{3,3})=n\sigma^{-2}u^1_{3,3}+n\log\left(u^1_{3,3}-u^0_{3,3}\right)+n\left(u^1_{3,3}-u^0_{3,3}\right)^{-1}\left(u^0_{3,3}-\left(u^1_{1,3}\right)^2\left(u^1_{1,1}\right)^{-1}\right)\\
&-2n\left(u^1_{3,3}-u^0_{3,3}\right)^{-1}\left(u^1_{2,3}-u^0_{2,3}\right)\left(u^1_{2,2}-u^0_{2,2}\right)^{-1}\left(u^0_{2,3}-u^1_{1,3}u^1_{1,2}\left(u^1_{1,1}\right)^{-1}\right)\\
&-n\left(u^1_{3,3}-u^0_{3,3}\right)^{-1}\left(u^1_{2,3}-u^0_{2,3}\right)^2\left(u^1_{2,2}-u^0_{2,2}\right)^{-1}\left(1-\left(u^1_{2,2}-u^0_{2,2}\right)^{-1}\left(u^0_{2,2}-\left(u^1_{1,2}\right)^2u^{-1}_{1,1}\right)\right)\\
&+n\log\det\tilde{\boldsymbol{K}}_{3,3}+nu^0_{3,3}\mathrm{Tr}\left(\tilde{\boldsymbol{K}}^{-1}_{3,3}\boldsymbol{K}^0_{3,3}\right)-n\mathrm{Tr}\left(\tilde{\boldsymbol{K}}^{-1}_{3,3}\tilde{\boldsymbol{K}}_{3,1}\tilde{\boldsymbol{K}}^{-1}_{1,1}\tilde{\boldsymbol{K}}_{1,3}\right)\\
&+2n\mathrm{Tr}\left(\tilde{\boldsymbol{K}}^{-1}_{3,3}\tilde{\boldsymbol{K}}_{3,2}\tilde{\boldsymbol{K}}^{-1}_{2,2}\left(\tilde{\boldsymbol{K}}_{2,1}\tilde{\boldsymbol{K}}^{-1}_{1,1}\tilde{\boldsymbol{K}}_{1,3}-u^0_{2,3}\boldsymbol{K}^0_{2,3}\right)\right)-n\mathrm{Tr}\left(\tilde{\boldsymbol{K}}^{-1}_{3,3}\tilde{\boldsymbol{K}}_{3,2}\left(\tilde{\boldsymbol{K}}^{-1}_{2,2}-\tilde{\boldsymbol{K}}^{-1}_{2,2}\left(u^0_{2,2}\boldsymbol{K}^0_{2,2}-\tilde{\boldsymbol{K}}_{2,1}\tilde{\boldsymbol{K}}^{-1}_{1,1}\tilde{\boldsymbol{K}}_{1,2}\right)\tilde{\boldsymbol{K}}^{-1}_{2,2}\right)\tilde{\boldsymbol{K}}_{2,3}\right)\\
&+n\left(\boldsymbol{Y}_3-\tilde{\boldsymbol{K}}_{3,2}\tilde{\boldsymbol{K}}^{-1}_{2,2}\left(\boldsymbol{Y}_2-\tilde{\boldsymbol{K}}_{2,1}\tilde{\boldsymbol{K}}^{-1}_{1,1}\boldsymbol{Y}_1\right)-\tilde{\boldsymbol{K}}_{3,1}\tilde{\boldsymbol{K}}^{-1}_{1,1}\boldsymbol{Y}_1\right)^\top\tilde{\boldsymbol{K}}^{-1}_{3,3}\left(\boldsymbol{Y}_3-\tilde{\boldsymbol{K}}_{3,2}\tilde{\boldsymbol{K}}^{-1}_{2,2}\left(\boldsymbol{Y}_2-\tilde{\boldsymbol{K}}_{2,1}\tilde{\boldsymbol{K}}^{-1}_{1,1}\boldsymbol{Y}_1\right)-\tilde{\boldsymbol{K}}_{3,1}\tilde{\boldsymbol{K}}^{-1}_{1,1}\boldsymbol{Y}_1\right)
\end{aligned}
\tag{133}
$$

B.4. Interpretation of Renormalization Factors. In this section we show that the renormalization factors introduced in SI 4.B.1 are directly linked to the norm and inner product of the readout weights. To show this, we can compute the readout norm by introducing moment generating term coupled to them. Specifically we introduce $L_{t,t'}$ coupled to $\boldsymbol{a}_t \cdot \boldsymbol{a}_{t'}$ in the replicated partition function, resulting in

$$\mathcal{M}\left(\boldsymbol{L}\right) = \lim_{n\to 0}\int\prod_{\alpha=1}^{n}\prod_{t=2}^{T}d\boldsymbol{v}_t^{\alpha}\int d\boldsymbol{v}_1^{n}\int\prod_{\alpha=1}^{n}\prod_{t=2}^{T}d\Theta_t^{\alpha}\int d\Theta_1^{n}\exp(-i\sum_{t,\alpha}\sum_{\mu=1}^{P}v_t^{\alpha,\mu}\left(f_t^{t}\left(\Theta_t^{\alpha},\boldsymbol{x}_t^{\mu}\right)-y_t^{\mu}\right)$$

$$-\frac{1}{2}\sigma^{-2}\sum_{t,\alpha}\|\Theta_t^{\alpha}\|^2-\frac{1}{2}\lambda\sum_{t=2}^{T}\sum_{\alpha=1}^{n}\|\mathcal{W}_t^{\alpha}-\mathcal{W}_{t-1}^{n}\|^2-\frac{1}{2}\beta^{-1}\sum_{t,\alpha}\boldsymbol{v}_t^{\alpha\top}\boldsymbol{v}_t^{\alpha}-\sum_{t,t'}L_{t,t'}\boldsymbol{a}_t^{n}\cdot\boldsymbol{a}_{t'}^{n}) \quad [134]$$

Thus by taking derivative of the MGF w.r.t. $\boldsymbol{L}$, we can derive

$$\langle\boldsymbol{a}_t\cdot\boldsymbol{a}_{t'}\rangle = -\frac{\partial\mathcal{M}(\boldsymbol{L})}{\partial L_{t,t'}}|_{\boldsymbol{L}=0} \quad [135]$$

We can perform the calculation in the same way as in SI 4.B.1, define

$$z_t^{\alpha} = N^{-1/2}\sigma\boldsymbol{v}_t^{\alpha\top}\Phi(\boldsymbol{w}_t^{\alpha},\boldsymbol{X}_t) \quad [136]$$

and we obtain

$$G(\{\boldsymbol{v}_t^{\alpha}\}_{\alpha=1,\cdots,n;t=1,\cdots,T}) = G(\boldsymbol{H})$$

$$= N\log\left[\int\prod_{\alpha=1}^{n}\prod_{t=1}^{T}dz_t^{\alpha}\det(\boldsymbol{H})^{-1/2}\det(\mathbb{I}+\sigma^2\boldsymbol{L})^{-1/2}\right.$$

$$\left.\exp\left(-\frac{1}{2}\sum_{t,\alpha}\sum_{t',\beta}z_t^{\alpha\top}[\boldsymbol{H}^{-1}]_{t,t',\alpha\beta}z_{t'}^{\beta}-\frac{1}{2}\sum_{t,\alpha}z_t^{\alpha}\left(\mathbb{I}+\sigma^2\boldsymbol{L}\right)^{-1}{}_{t,t',\alpha,\beta}z_{t'}^{\beta}\right)\right] \quad [137]$$

$$= -\frac{N}{2}\log\det(\boldsymbol{H}+\mathbb{I}+\sigma^2\boldsymbol{L}) \quad [138]$$

in place of SI Eq. 115, and

$$\mathcal{M}\left(\boldsymbol{L}\right) = \lim_{n\to 0}\int\prod_{\alpha=1}^{n}\prod_{t=1}^{T}d\boldsymbol{v}_t^{\alpha}\int d\boldsymbol{U}\int d\boldsymbol{H}\exp(-\frac{1}{2}\beta^{-1}\sum_{t,\alpha}\boldsymbol{v}_t^{\alpha\top}\boldsymbol{v}_t^{\alpha}+i\sum_{t,\alpha}\boldsymbol{v}_t^{\alpha\top}\boldsymbol{Y}_t-\frac{N}{2}\log\det(\mathbb{I}+\boldsymbol{H}+\sigma^2\boldsymbol{L})$$

$$+\sigma^{-2}\frac{N}{2}\mathrm{Tr}(\boldsymbol{U}\boldsymbol{H})-\frac{1}{2}\sum\sum_{t',\beta}U_{t,t',\alpha,\beta}\boldsymbol{v}_t^{\alpha\top}\Phi(\boldsymbol{w}_t^{\alpha},\boldsymbol{X}_t)\cdot\Phi(\boldsymbol{w}_{t'}^{\beta},\boldsymbol{X}_{t'})\boldsymbol{v}_{t'}^{\beta}) \quad [139]$$

in place of SI Eq. 116.

Taking derivative of $\boldsymbol{H}$ and setting it to 0, we obtain,

$$\sigma^{-2}\boldsymbol{U} = \left(\mathbb{I}+\boldsymbol{H}+\sigma^2\boldsymbol{L}\right)^{-1} \quad [140]$$

Plugging into SI Eq. 139 yields

$$\mathcal{M}\left(\boldsymbol{L}\right) = \lim_{n\to 0}\int\prod_{\alpha=1}^{n}\prod_{t=1}^{T}d\boldsymbol{v}_t^{\alpha}\int d\boldsymbol{U}\int d\boldsymbol{H}\exp(-\frac{1}{2}\beta^{-1}\sum_{t,\alpha}\boldsymbol{v}_t^{\alpha\top}\boldsymbol{v}_t^{\alpha}+i\sum_{t,\alpha}\boldsymbol{v}_t^{\alpha\top}\boldsymbol{Y}_t+\frac{N}{2}\log\det(\boldsymbol{U})$$

$$-\sigma^{-2}\frac{N}{2}\mathrm{Tr}(\boldsymbol{U})-\frac{N}{2}\mathrm{Tr}(\boldsymbol{U}\boldsymbol{L})-\frac{1}{2}\sum\sum_{t',\beta}U_{t,t',\alpha,\beta}\boldsymbol{v}_t^{\alpha\top}\Phi(\boldsymbol{w}_t^{\alpha},\boldsymbol{X}_t)\cdot\Phi(\boldsymbol{w}_{t'}^{\beta},\boldsymbol{X}_{t'})\boldsymbol{v}_{t'}^{\beta}) \quad [141]$$

in place of Supplementary Eq. 117.

Taking derivative w.r.t. $\boldsymbol{L}$, we have

$$\langle\boldsymbol{a}_t,\boldsymbol{a}_t\rangle = NU_{t,t',n,n} \quad [142]$$

, and thus

$$N^{-1}\langle\boldsymbol{a}_t\cdot\boldsymbol{a}_{t'}\rangle = u_{t,t'}^{1} \quad [143]$$

The renormalization factors $\left\{u_{t,t'}^{1}\right\}_{t,t'=1,\cdots,T}$ are directly related the the covariances of the readout weights. In the specific case of $T=2$,

$$u_{2,2}^{1} = N^{-1}\langle\|\boldsymbol{a}_2\|^2\rangle \quad [144]$$

$$u_{2,1}^{1} = N^{-1}\langle\boldsymbol{a}_2\cdot\boldsymbol{a}_1\rangle \quad [145]$$

The renormalization factors $\left\{u_{t,t'}^{0}\right\}_{t,t'=1,\cdots,T}$ are less interpretable, the difference between $\left\{u_{t,t'}^{1}\right\}_{t,t'=1,\cdots,T}$ and $\left\{u_{t,t'}^{0}\right\}_{t,t'=1,\cdots,T}$ can be related to the susceptibility of the readout weights, but we leave out the detailed calculation for simplicity.

B.5. Predicting Phase Transition Boundaries with the Task Similairity OP. Even when there are only two tasks, SI Eqs. 129-132 need to be solved numerically in general. In the $\lambda \to \infty$ limit, we can analyze the scaling of the solutions with λ. Interestingly, we find 3 different consistent scalings of the solutions with λ. $u^1_{1,1}$ only depends on the first task, and is always $\mathcal{O}(1)$ w.r.t. λ. In one regime, we have $u^1_{2,2}, u^0_{2,2}, u^1_{2,2} - u^0_{2,2} \sim \mathcal{O}(1)$, SI Eqs. 132-130 can be simplified as

$$u^1_{1,2} = 0 \quad [146]$$

$$u^0_{2,2} = \frac{1}{N(1-\alpha)} \mathbf{Y}_2^\top \left(\boldsymbol{K}^1_{2,2}\right)^{-1} \mathbf{Y}_2 \quad [147]$$

$$u^1_{2,2} - u^0_{2,2} = \sigma^2(1-\alpha) \quad [148]$$

Note that since $u^1_{2,2} > u^0_{2,2}$ and $u^0_{2,2} > 0$ (see SI 4.B.4), this solution can only hold when $\alpha < 1$. In particular, in the infinite-width limit, $\alpha = 0$, and we have $u^0_{2,2} = 0, u^1_{2,2} = \sigma^2$. This result is equivalent to learning each task individually, with Gaussian random hidden-layer weights.

When $\alpha > 1$, there are two consistent scalings of the solutions with λ. In one regime, we have $u^1_{2,2}, u^0_{2,2} \sim \mathcal{O}(\lambda^{1/2}), u^1_{2,2} - u^0_{2,2} \sim \mathcal{O}(\lambda^{-1/2})$, $u^1_{1,2} \sim \mathcal{O}(1)$. This corresponds to the overfitting regime in. To the leading order, SI Eq. 130 remains the same, SI Eqs. 132, 131 are simplified as

$$1 - \frac{1}{N}\left(u^1_{2,2} - u^0_{2,2}\right)^2 \mathrm{Tr}\left(\tilde{\boldsymbol{K}}^{-1}_{2,2}\boldsymbol{K}^1_{2,2}\tilde{\boldsymbol{K}}^{-1}_{2,2}\boldsymbol{K}^1_{2,2}\right) = 0 \quad [149]$$

$$\begin{aligned}\sigma^{-2}u^0_{2,2} - \left(u^1_{1,2}\right)^2\left(u^1_{1,1}\right)^{-1}\left(u^1_{2,2} - u^0_{2,2}\right)^{-1} + \frac{1}{N}\mathrm{Tr}\left(\tilde{\boldsymbol{K}}_{2,1}\tilde{\boldsymbol{K}}^{-1}_{1,1}\tilde{\boldsymbol{K}}_{1,2}\tilde{\boldsymbol{K}}^{-1}_{2,2}\right)\\ - \frac{1}{N}\left(\mathbf{Y}_2 - \tilde{\boldsymbol{K}}_{2,1}\tilde{\boldsymbol{K}}^{-1}_{1,1}\mathbf{Y}_1\right)^\top \tilde{\boldsymbol{K}}^{-1}_{2,2}\left(\mathbf{Y}_2 - \tilde{\boldsymbol{K}}_{2,1}\tilde{\boldsymbol{K}}^{-1}_{1,1}\mathbf{Y}_1\right) = 0\end{aligned} \quad [150]$$

In the other regime, we have $u^1_{2,2}, u^0_{2,2}, u^1_{1,2} \sim \mathcal{O}(1), u^1_{2,2} - u^0_{2,2} \sim \mathcal{O}(\lambda^{-1})$, this corresponds to the generalization regime. To the leading order, SI Eqs. 130, 132 remain the same, SI Eq. 131 is simplified as

$$\begin{aligned}&\left(u^1_{2,2} - u^0_{2,2}\right)^{-1} + u^0_{2,2}\frac{1}{N}\mathrm{Tr}\left(\tilde{\boldsymbol{K}}^{-1}_{2,2}\Delta\boldsymbol{K}_{2,2}\tilde{\boldsymbol{K}}^{-1}_{2,2}\boldsymbol{K}^{L,0}_{2,2}\right) - \frac{1}{N}\mathrm{Tr}\left(\tilde{\boldsymbol{K}}^{-1}_{2,2}\boldsymbol{K}^{L,1}_{2,2}\right)\\ &- \frac{1}{N}\mathrm{Tr}\left(\tilde{\boldsymbol{K}}_{2,1}\tilde{\boldsymbol{K}}^{-1}_{1,1}\tilde{\boldsymbol{K}}^\top_{2,1}\tilde{\boldsymbol{K}}^{-1}_{2,2}\Delta\boldsymbol{K}_{2,2}\tilde{\boldsymbol{K}}^{-1}_{2,2}\right) + \frac{1}{N}\left(\mathbf{Y}_2 - \tilde{\boldsymbol{K}}_{2,1}\tilde{\boldsymbol{K}}^{-1}_{1,1}\mathbf{Y}_1\right)^\top\tilde{\boldsymbol{K}}^{-1}_{2,2}\Delta\boldsymbol{K}_{2,2}\tilde{\boldsymbol{K}}^{-1}_{2,2}\left(\mathbf{Y}_2 - \tilde{\boldsymbol{K}}_{2,1}\tilde{\boldsymbol{K}}^{-1}_{1,1}\mathbf{Y}_1\right) = 0\end{aligned} \quad [151]$$

It is difficult to see where the transition between the two scalings occur in general. Therefore, we make a further simplification assuming that the kernels $\boldsymbol{K}^1_{2,2}$,$\boldsymbol{K}^0_{2,2}$, $\tilde{\boldsymbol{K}}_{2,2}$ and $\Delta\boldsymbol{K}_{2,2}$ are different only in their magnitudes. To the leading order $\boldsymbol{K}^1_{2,2} = \boldsymbol{K}^0_{2,2}$

$$\Delta\boldsymbol{K}_{2,2} \approx \sigma^{-2}\lambda^{-1}\boldsymbol{K}^1_{2,2} \sim \mathcal{O}(\lambda^{-1}) \quad [152]$$

$$\tilde{\boldsymbol{K}}_{2,2} \approx \left(\left(u^1_{2,2} - u^0_{2,2}\right) + \sigma^{-2}\lambda^{-1}u^0_{2,2}\right)\boldsymbol{K}^1_{2,2} \quad [153]$$

This approximation is exact for linear networks, for nonlinear networks $\boldsymbol{K}^0_{2,2}$ and $\boldsymbol{K}^1_{2,2}$ are not only different in their scales but also their magnitude, and the approximation is only heuristic. In Figs. 5 in the main text, we see that the approximation is very accurate. With this approximation SI Eqs. 149 , 150 further simplify to

$$\left(1 + \sigma^{-2}\lambda^{-1}\frac{u^0_{2,2}}{u^1_{2,2} - u^0_{2,2}}\right)^2 = \alpha \quad [154]$$

$$\begin{aligned}&\sigma^{-2}\alpha^{1/2}u^0_{2,2}(u^1_{2,2} - u^0_{2,2})u^1_{1,1} - \alpha^{1/2}\left(u^1_{1,2}\right)^2 + \left(u^1_{1,2}\right)^2\frac{1}{N}\mathrm{Tr}\left(\boldsymbol{K}^1_{2,1}\left(\boldsymbol{K}^1_{1,1}\right)^{-1}\boldsymbol{K}^1_{2,1}\left(\boldsymbol{K}^1_{2,2}\right)^{-1}\right)\\ &- \frac{1}{N}u^1_{1,1}\mathbf{Y}_2^\top\left(\boldsymbol{K}^1_{2,2}\right)^{-1}\mathbf{Y}_2 + 2u^1_{1,2}\frac{1}{N}\mathbf{Y}_2^\top\left(\boldsymbol{K}^1_{2,2}\right)^{-1}\boldsymbol{K}^1_{2,1}\left(\boldsymbol{K}^1_{1,1}\right)^{-1}\mathbf{Y}_1\\ &- \frac{1}{N}\left(u^1_{1,2}\right)^2\left(u^1_{1,1}\right)^{-1}\mathbf{Y}_1^\top\left(\boldsymbol{K}^1_{1,1}\right)^{-1}\boldsymbol{K}^1_{1,2}\left(\boldsymbol{K}^1_{2,2}\right)^{-1}\boldsymbol{K}^1_{2,1}\left(\boldsymbol{K}^1_{1,1}\right)^{-1}\mathbf{Y}_1 = 0\end{aligned} \quad [155]$$

For this solutions to be valid, it is evident that we require $\alpha > 1$, confirming the phase-transition boundary at $\alpha = 1$. Furthermore, since $u^0_{2,2}, u^1_{2,2}, u^1_{2,2} - u^0_{2,2} > 0$, we have

$$\begin{aligned}&\alpha^{-1/2}u^1_{1,2} - u^1_{1,2}\frac{1}{P}\mathrm{Tr}\left(\boldsymbol{K}^1_{2,1}\left(\boldsymbol{K}^1_{1,1}\right)^{-1}\boldsymbol{K}^1_{2,1}\left(\boldsymbol{K}^1_{2,2}\right)^{-1}\right) + \frac{1}{P}u^1_{1,1}\left(u^1_{1,2}\right)^{-1}\mathbf{Y}_2^\top\left(\boldsymbol{K}^1_{2,2}\right)^{-1}\mathbf{Y}_2 - 2\frac{1}{P}\mathbf{Y}_2^\top\left(\boldsymbol{K}^1_{2,2}\right)^{-1}\boldsymbol{K}^1_{2,1}\left(\boldsymbol{K}^1_{1,1}\right)^{-1}\mathbf{Y}_1\\ &+ \frac{1}{P}u^1_{1,2}\left(u^1_{1,1}\right)^{-1}\mathbf{Y}_1^\top\left(\boldsymbol{K}^1_{1,1}\right)^{-1}\boldsymbol{K}^1_{1,2}\left(\boldsymbol{K}^1_{2,2}\right)^{-1}\boldsymbol{K}^1_{2,1}\left(\boldsymbol{K}^1_{1,1}\right)^{-1}\mathbf{Y}_1 > 0\end{aligned} \quad [156]$$

This condition determines the phase-transition boundary between the overfitting regime ($u^1_{2,2}, u^0_{2,2} \sim \mathcal{O}(\lambda^{1/2}), u^1_{2,2} - u^0_{2,2} \sim \mathcal{O}(\lambda^{-1/2})$) and the generalization regime ($u^1_{2,2}, u^0_{2,2} \sim \mathcal{O}(1), u^1_{2,2} - u^0_{2,2} \sim \mathcal{O}(\lambda^{-1})$). By simplifying SI Eq. 130 , solving for $u^1_{1,2}$, and plugging in SI Eq. 156, we obtain

$$\begin{aligned}&\frac{1}{P}\boldsymbol{Y}_1^\top\left(\boldsymbol{K}_{1,1}^1\right)^{-1}\boldsymbol{K}_{1,2}^1\left(\boldsymbol{K}_{2,2}^1\right)^{-1}\boldsymbol{K}_{2,1}^1\left(\boldsymbol{K}_{1,1}^1\right)^{-1}\boldsymbol{Y}_1-\left(\frac{1}{P}\boldsymbol{Y}_1^\top\left(\boldsymbol{K}_{1,1}^1\right)^{-1}\boldsymbol{K}_{1,2}^1\left(\boldsymbol{K}_{2,2}^1\right)^{-1}\boldsymbol{Y}_2\right)^2\cdot\frac{1}{P}\boldsymbol{Y}_2^\top\left(\boldsymbol{K}_{2,2}^1\right)^{-1}\boldsymbol{Y}_2\\&>u_{1,1}^1\left(\frac{1}{P}\mathrm{Tr}\left(\boldsymbol{P}_1\boldsymbol{P}_2\right)-\alpha^{-1/2}\right)\end{aligned}\qquad [157]$$

where $u_{1,1}^1$ is given by SI Eq. 129. Since we are in the $\lambda\to\infty$ limit, the kernel functions $K_{t,t'}^1$ are time invariant, and therefore the kernels in SI Eq. 157 are given by $\boldsymbol{K}_{t,t'}^1=K_{GP}(\boldsymbol{X}_t,\boldsymbol{X}_{t'})\ t,t'\in\{1,2\}$. We have thus derived the phase-transition boundary and the relevant OPs as given in SI Eqs. 95, 96 in SI 4.A.

By properly normalizing the data, we can further simplify SI Eq. 157. Assuming that $\sigma^2=1$ and the data is normalized such that $\frac{1}{P}=1$, and $\frac{1}{P}=1$, SI Eq. 157 can be simplified as

$$-\frac{1}{P}\boldsymbol{Y}_1^\top\left(\boldsymbol{K}_{1,1}^1\right)^{-1}\boldsymbol{K}_{1,2}^1\left(\boldsymbol{K}_{2,2}^1\right)^{-1}\boldsymbol{K}_{2,1}^1\left(\boldsymbol{K}_{1,1}^1\right)^{-1}\boldsymbol{Y}_1+\left(\frac{1}{P}\boldsymbol{Y}_1^\top\left(\boldsymbol{K}_{1,1}^1\right)^{-1}\boldsymbol{K}_{1,2}^1\left(\boldsymbol{K}_{2,2}^1\right)^{-1}\boldsymbol{Y}_2\right)^2+\frac{1}{P}\mathrm{Tr}\left(\boldsymbol{P}_1\boldsymbol{P}_2\right)<\alpha^{-1/2}\qquad [158]$$

If this inequality holds, the network in the overfitting regime, otherwise the network is in the generalization regime, thus setting the inequality to equality gives rise to the phase transition boundary, as given in SI Eq.97. We define the r.h.s. of Eq. 158 as γ_{sim}, which ranges from -1 to 1.

B.6. Hidden-Layer Kernels and Representational Changes.

Hidden-Layer Kernels To estimate the hidden-layer kernels, we make a heuristic approximation. We assume that the probability distributions of $\Phi(\mathcal{W}_2^\alpha,\boldsymbol{x})$ and $\Phi(\mathcal{W}_1,\boldsymbol{x})$ induced by the Gaussian prior proportional to $\exp(-S_0(\mathcal{W}))$ (SI Eq.34), can be approximated as Gaussian distributions with mean 0 and covariances

$$N^{-1}\langle\Phi(\mathcal{W}_1,\boldsymbol{x})\cdot\Phi(\mathcal{W}_1,\boldsymbol{x}')\rangle_{\mathcal{W}}=K_{1,1}^1(\boldsymbol{x},\boldsymbol{x}')\qquad [159]$$

$$N^{-1}\langle\Phi(\mathcal{W}_2^\alpha,\boldsymbol{x})\cdot\Phi(\mathcal{W}_2^\beta,\boldsymbol{x}')\rangle_{\mathcal{W}}=\begin{cases}K_{2,2}^1(\boldsymbol{x},\boldsymbol{x}') & \alpha=\beta\\ K_{2,2}^0(\boldsymbol{x},\boldsymbol{x}') & \alpha\neq\beta\end{cases}\qquad [160]$$

$$N^{-1}\langle\Phi(\mathcal{W}_1,\boldsymbol{x})\cdot\Phi(\mathcal{W}_2^\alpha,\boldsymbol{x}')\rangle=K_{1,2}^1(\boldsymbol{x},\boldsymbol{x}')\qquad [161]$$

This approximation is exact for linear networks, and allows us to evaluate second moments of the representations over the posterior distribution in Θ by evaluating Gaussian integrals. In particular, we define the similarity between representations (i.e. the hidden-layer kernels) as

$$K_{\mathrm{sim}}(\boldsymbol{x},\boldsymbol{x}')\equiv N^{-1}\langle\Phi(\mathcal{W}_2,\boldsymbol{x})\cdot\Phi(\mathcal{W}_2,\boldsymbol{x}')\rangle\qquad [162]$$

where the average is w.r.t. the posterior distribution of Θ for $T=2$.We have

$$\begin{aligned}K_{\mathrm{sim}}(\boldsymbol{x},\boldsymbol{x}')=K_{2,2}^1(\boldsymbol{x},\boldsymbol{x}')-\frac{1}{N}\Big(&u_{1,1}^1\boldsymbol{k}_{2,1}^1(\boldsymbol{x})^\top\langle\boldsymbol{v}_1\boldsymbol{v}_1^\top\rangle\boldsymbol{k}_{1,2}^1(\boldsymbol{x}')+u_{2,2}^0\Delta\boldsymbol{k}_{2,2}(\boldsymbol{x})^\top\langle\boldsymbol{v}_2\boldsymbol{v}_2^\top\rangle\Delta\boldsymbol{k}_{2,2}(\boldsymbol{x}')\\&+u_{1,2}^1\boldsymbol{k}_{2,1}^1(\boldsymbol{x})^\top\langle\boldsymbol{v}_1\boldsymbol{v}_2^\top\rangle\Delta\boldsymbol{k}_{2,2}(\boldsymbol{x}')+u_{1,2}^1\Delta\boldsymbol{k}_{2,2}(\boldsymbol{x})^\top\langle\boldsymbol{v}_2\boldsymbol{v}_1^\top\rangle\boldsymbol{k}_{1,2}^1(\boldsymbol{x}')\\&+u_{2,2}^0\boldsymbol{k}_{2,2}^0(\boldsymbol{x})^\top\langle\delta\boldsymbol{v}_2\delta\boldsymbol{p}_2^\top\rangle\Delta\boldsymbol{k}_{2,2}(\boldsymbol{x}')+u_{2,2}^0\Delta\boldsymbol{k}_{2,2}(\boldsymbol{x})^\top\langle\delta\boldsymbol{v}_2\delta\boldsymbol{p}_2^\top\rangle\boldsymbol{k}_{2,2}^0(\boldsymbol{x}')\\&+\left(u_{2,2}^1-u_{2,2}^0\right)\left(\boldsymbol{k}_{2,2}^1(\boldsymbol{x})^\top\langle\boldsymbol{v}_2\boldsymbol{v}_2^\top\rangle\boldsymbol{k}_{2,2}^1(\boldsymbol{x}')-\boldsymbol{k}_{2,2}^0(\boldsymbol{x})^\top\langle\boldsymbol{v}_2\boldsymbol{v}_2^\top\rangle\boldsymbol{k}_{2,2}^0(\boldsymbol{x}')\right)\Big)\end{aligned}\qquad [163]$$

where the statistics of $\boldsymbol{v}_2,\boldsymbol{p}_2$ and $\boldsymbol{v}_1$ are given in SI Eqs. 51, 54, 55, but with $\left\{m_{t,t'}^1\right\}_{t,t'=1,\cdots,T},\left\{m_{t,t'}^0\right\}_{t,t'=1,\cdots,T}$ replaced by $\left\{u_{t,t'}^1\right\}_{t,t'=1,\cdots,T},\left\{u_{t,t'}^0\right\}_{t,t'=1,\cdots,T}$. $K_{\mathrm{sim}}(\boldsymbol{x},\boldsymbol{x}')$ has two contributions, the first term in SI Eq. 163 corresponds to averaging SI Eq. 162 w.r.t. the Gaussian prior in $\mathcal{W}_2$, and the rest of the terms are induced by learning, which we denote as $K(\boldsymbol{x},\boldsymbol{x}')$. $K(\mathbf{x},\mathbf{x}')$ thus captures the changes in the similarity matrix before learning (with Gaussian prior weights) and after learning.

Although the learning induced terms are sub-leading and of $\mathcal{O}(1/N)$, we see in Fig. 7 in the main text that the structure of these terms can affect the generalization performance. We note that, in the overfitting regime, because $u_{2,2}^1,u_{2,2}^0\sim\mathcal{O}(\lambda^{1/2})$ and $u_{2,2}^1-u_{2,2}^0\sim\mathcal{O}(\lambda^{-1/2})$, several terms vanish in the large λ limit, and we have

$$\begin{aligned}K_{\mathrm{sim}}(\boldsymbol{x},\boldsymbol{x}')=K_{2,2}^1(\boldsymbol{x},\boldsymbol{x}')-\frac{1}{N}\Big(&u_{1,1}^1\boldsymbol{k}_{2,1}^1(\boldsymbol{x})^\top\langle\boldsymbol{v}_1\boldsymbol{v}_1^\top\rangle\boldsymbol{k}_{1,2}^1(\boldsymbol{x}')-\left(u_{2,2}^0\right)^2\Delta\boldsymbol{k}_{2,2}(\boldsymbol{x})^\top\tilde{\boldsymbol{K}}_{2,2}^{-1}\boldsymbol{K}_{2,2}^0\tilde{\boldsymbol{K}}_{2,2}^{-1}\Delta\boldsymbol{k}_{2,2}(\boldsymbol{x}')\\&+u_{2,2}^0\boldsymbol{k}_{2,2}^0(\boldsymbol{x})^\top\tilde{\boldsymbol{K}}_{2,2}^{-1}\Delta\boldsymbol{k}_{2,2}(\boldsymbol{x}')+u_{2,2}^0\Delta\boldsymbol{k}_{2,2}(\boldsymbol{x})^\top\tilde{\boldsymbol{K}}_{2,2}^{-1}\boldsymbol{k}_{2,2}^0(\boldsymbol{x}')\\&-\left(u_{2,2}^1-u_{2,2}^0\right)u_{2,2}^0\left(\boldsymbol{k}_{2,2}^1(\boldsymbol{x})^\top\tilde{\boldsymbol{K}}_{2,2}^{-1}\boldsymbol{K}_{2,2}^0\tilde{\boldsymbol{K}}_{2,2}^{-1}\boldsymbol{k}_{2,2}^1(\boldsymbol{x}')-\boldsymbol{k}_{2,2}^0(\boldsymbol{x})^\top\tilde{\boldsymbol{K}}_{2,2}^{-1}\boldsymbol{K}_{2,2}^0\tilde{\boldsymbol{K}}_{2,2}^{-1}\boldsymbol{k}_{2,2}^0(\boldsymbol{x}')\right)\Big)\end{aligned}\qquad [164]$$

The structure of $K_{\mathrm{sim}}(\boldsymbol{x},\boldsymbol{x}')$ is not affected by the labels of the second task $\boldsymbol{Y}_2$, confirming our observation that the network fails to learn task 2 relevant representations in this regime. In the main text Fig. 7, we evaluate the kernel on the training data, $K_{\mathrm{sim}}(\boldsymbol{X}_1)\equiv K_{\mathrm{sim}}(\boldsymbol{X}_1,\boldsymbol{X}_1)\in\mathbb{R}^{P\times P}$, $K_{\mathrm{sim}}(\boldsymbol{X}_2)\equiv K_{\mathrm{sim}}(\boldsymbol{X}_2,\boldsymbol{X}_2)\in\mathbb{R}^{P\times P}$. Similarly, $K(\boldsymbol{X}_1)\equiv K(\boldsymbol{X}_1,\boldsymbol{X}_1)\in\mathbb{R}^{P\times P}$, $K(\boldsymbol{X}_2)\equiv K(\boldsymbol{X}_2,\boldsymbol{X}_2)\in\mathbb{R}^{P\times P}$ correspond to the learning-induced terms in $K_{\mathrm{sim}}(\boldsymbol{X}_1)$ and $K_{\mathrm{sim}}(\boldsymbol{X}_2)$.

Representational Changes For the changes in the representation of $\boldsymbol{x}$ defined as $\Delta\Phi(\boldsymbol{x}) = \Phi(\mathcal{W}_2, \boldsymbol{x}) - \Phi(\mathcal{W}_1, \boldsymbol{x})$, we can derive the second moments in $\Delta\Phi(\boldsymbol{x})$ using the same Gaussian approximation for calculating the hidden-layer kernels. In the $\lambda \to \infty$ limit, we obtain

$$\Delta\Phi(\boldsymbol{x}) \cdot \Delta\Phi(\boldsymbol{x}') = -u^0_{2,2}\Delta\boldsymbol{k}_{2,2}(\boldsymbol{x})^\top \langle \boldsymbol{v}_2 \boldsymbol{v}_2^\top \rangle \Delta\boldsymbol{k}_{2,2}(\boldsymbol{x}') \qquad [165]$$

By analyzing the scalings of $u^1_{2,2}, u^0_{2,2}, u^1_{2,2} - u^0_{2,2}$ and $u^1_{1,2}$ in the different regimes in the $\lambda \to \infty$ limit, we can analyze the behavior of $\Delta\Phi(\boldsymbol{x}) \cdot \Delta\Phi(\boldsymbol{x}')$. In the regime $\alpha < 1$, we have $\Delta\Phi(\boldsymbol{x}) \cdot \Delta\Phi(\boldsymbol{x}') = 0$, confirming that the hidden-layer weights do not change from task 1 to task 2. In the overfitting regime, by evaluating $\langle \boldsymbol{v}_2 \boldsymbol{v}_2^\top \rangle$ with the corresponding scaling of the renormalization factors, we have

$$\Delta\Phi(\boldsymbol{x}) \cdot \Delta\Phi(\boldsymbol{x}') = \left(u^0_{2,2}\right)^2 \Delta\boldsymbol{k}_{2,2}(\boldsymbol{x})^\top \tilde{\boldsymbol{K}}^{-1}_{2,2} \boldsymbol{K}^0_{2,2} \tilde{\boldsymbol{K}}^{-1}_{2,2} \Delta\boldsymbol{k}_{2,2}(\boldsymbol{x}') \qquad [166]$$

In the generalization regime, we have

$$\begin{aligned}\Delta\Phi(\boldsymbol{x}) \cdot \Delta\Phi(\boldsymbol{x}') = &\left(u^0_{2,2}\right)^2 \Delta\boldsymbol{k}_{2,2}(\boldsymbol{x})^\top \tilde{\boldsymbol{K}}^{-1}_{2,2} \boldsymbol{K}^0_{2,2} \tilde{\boldsymbol{K}}^{-1}_{2,2} \Delta\boldsymbol{k}_{2,2}(\boldsymbol{x}') \\ &+ u^0_{2,2} \Delta\boldsymbol{k}_{2,2}(\boldsymbol{x})^\top \tilde{\boldsymbol{K}}^{-1}_{2,2} \left(\boldsymbol{Y}_2 - \tilde{\boldsymbol{K}}_{2,1} \tilde{\boldsymbol{K}}^{-1}_{1,1} \boldsymbol{Y}_1\right) \left(\boldsymbol{Y}_2 - \tilde{\boldsymbol{K}}_{2,1} \tilde{\boldsymbol{K}}^{-1}_{1,1} \boldsymbol{Y}_1\right)^\top \tilde{\boldsymbol{K}}^{-1}_{2,2} \Delta\boldsymbol{k}_{2,2}(\boldsymbol{x}')\end{aligned} \qquad [167]$$

As long as $\alpha > 1$, $\|\Delta\Phi(\boldsymbol{x})\|^2 > 0$. Therefore, $F_{2,1} = 0$ in the overfitting regime suggests that $\Delta\Phi(\boldsymbol{x})$ has to be in the null space of $\boldsymbol{a}_1$. Furthermore, Eqs. 166, 167 shows that the overfitting regime the structure of $\Delta\Phi(\boldsymbol{x}) \cdot \Delta\Phi(\boldsymbol{x}')$ is not aligned with either $\boldsymbol{Y}_1$ nor $\boldsymbol{Y}_2$, and in the generalization regime the structure is affected by both $\boldsymbol{Y}_1$ and $\boldsymbol{Y}_2$.

Part II
Details and Parameters of Numerical Experiments

5. The Target-Distractor Task Sequence

To construct the target-distractor task sequence, we used the CIFAR-100 source dataset (8), converted to grayscale. As a preprocessing step, all images are centered (zero-meaned), whitened, and normalized (such that the squared norm of every image is the input dimension of each source dataset). Each image is randomly labeled as ± 1 or 0. In all tasks, $x\%$ of the images are drawn from the ones with ± 1 labels, and the rest are drawn from the ones labeled 0. For each task sequence, among the images with ± 1 labels, a fraction of ρ_{target} images are shared across all tasks within the sequence, and the rest are unique for each task. Among the images with 0 labels, a fraction of $\rho_{\text{shared}} - x\% \cdot \rho_{\text{target}}$ images are shared across all tasks within the sequence, and the rest are unique for each task. Therefore a total fraction of ρ_{shared} images are the same across all tasks in the sequence, and the rest of the images are unique for each task. Among the images with ± 1 labels and are shared across all tasks within the sequence, the signs of the labels are flipped with probability ρ_{flipped}. ρ_{flipped} can be varied within the range of $[0, 0.5]$ independently of the other two parameters. For ρ_{shared} in the range of $[x\%, 1 - x\%]$, ρ_{target} can assume any values in $[0, 1]$, such that ρ_{target} and ρ_{shared} can be varied independently. However, for $\rho_{\text{shared}} \in [0, x\%]$, ρ_{target} can only be in the range of $[0, \rho_{\text{shared}}]$. Similarly if $\rho_{\text{shared}} \in [1 - x\%, 1]$, ρ_{target} can only be in the range of $[\rho_{\text{target}}, 1]$. Therefore, choosing a small $x\%$ allows ρ_{target} and ρ_{shared} to be manipulated more independently. In all our results we chose $x\% = 10\%$. We also fixed $P = 2000$, in Fig. 3 $L = 10$ and in Fig. 5 $L = 1$.

In Fig. 3**c**, we computed the normalized PVE (proportion of variance explained, (9)) of $F_{2,1}$ and τ_F. For $F_{2,1}$, we first employed a multiple linear regression model with $F_{2,1}$ as the dependent variable and γ_{RF}, γ_{rule}, γ_{feature} as the independent variables. We calculated the total R^2 value of this regression model to represent the proportion of variance in $F_{2,1}$ collectively explained by all three variables. To evaluate the contribution of each variable individually, we computed the R^2 values of the reduced models where each variable was removed one at a time. The PVE is then computed as the difference between the R^2 of the full model and the R^2 of the reduced model, divided by the R^2 of the full model. We then normalized the PVE such that the the PVE's of the three variables sum up to 1. The same procedure is done to calculate the PVE of τ_F.

In Fig. 4**b**,**d**, we showed the generalization performance $G_{2,2}$ using the target-distractor task sequence, to illustrate its divergent behavior. The test data is not defined for the target-distractor task sequence, as the images are randomly labeled, we thus evaluated the 'generalization' using perturbed images of the training data by adding Gaussian noise to the inputs. The standard deviation of the noise is fixed at 0.2. In Fig. 4**f**, we showed the transition boundary α_c, estimated using the shape of $F_{2,1}$ as a function of α. Specifically, α_c was estimated as the point where $F_{2,1}$ exhibited the most rapid change with respect to α.

6. Benchmark Task Sequences

All source datasets used (MNIST (10), EMNIST (11), Fashion-MNIST (12), and CIFAR-100 (8)) are image classification datasets. The images are either grayscale (MNIST, EMNIST, Fashion-MNIST) or converted to grayscale (CIFAR-100). Similarly as for the target-distractor task sequence, as preprocessing, all images are centered, whitened, and normalized. All of our analysis used subsets of $P = 2000$ images in the training set of each task to save computational cost, as commonly done in theoretical studies of deep NNs (e.g., (1, 6)) – the subset of images used are redrawn for each random seed used. As shown in Fig. S2, further increasing the number of images do not significantly improve generalization performance on previously learned tasks. The specific protocols used for generating task sequences from source datasets are detailed below. In all cases we use the MNIST dataset, which consists of images of digits "0" through "9", as an example to explain the protocols.

A. Permutation. Our permutation protocol largely follows standard practices in the literature (13). Each source dataset is first turned into a binary classification dataset by randomly dividing the original image classes (e.g., "0" through "9") into two groups. Images from one group are assigned target label $+1$ and those from the other are assigned -1. All training and test images corresponding to the same task undergo the same randomly generated pixel permutation, where the fraction of pixels permuted (relative to the original unpermuted images) is termed the "permutation ratio". The permutation is independently generated for each task. Inputs in each of $D_1, ..., D_T$ are permuted versions of the same subset of images. At zero permutation $D_1 = D_2 = ... = D_T$.
Note that the protocol above differs from standard practices in that we also permuted images in D_1 – this is to ensure such that any pair of tasks in a long sequence have the same statistical relations (Fig. 4). In all analysis of multi-head CL, as we focused on CL of two tasks, we followed standard practices and did not permuted D_1 (Figs. 6, 7).

B. Split. Our split protocol also largely follows standard practices in the literature (13, 14). Each task contains only images from a disjoint set of classes (e.g., task 1 is "0" vs. "1", task 2 is "2" vs. "3"). Thus, the maximum length of the sequence is limited by the number of classes in the source dataset – since MNIST and Fashion-MNIST each contains 10 classes, they can produce sequences with at most five tasks. On the other hand, CIFAR-100 contains 100 classes and EMNIST contains 62 classes, allowing for much longer sequences. Thus, our analysis of long-term forgetting only applies the split protocol to CIFAR-100 and EMNIST (Fig. 4). In EMNIST, each task includes a disjoint pair of classes. In CIFAR-100, we are limited by the amount of examples available for each class (500 images per class in the training set), in order to have $P = 2000$, each task includes a disjoint set of 4 classes, and the task is to classify 2 of these classes vs. the other 2. Each random seed corresponds to a different subsample of images from each class being used, as well as a random assignment of classes into disjoint sets.

In the special case of having only two tasks (Figs. 6, 7), we designed a "partial split" protocol to parametrically vary relations between D_1 and D_2. As an example, suppose the first pair of classes is "0" and "1". The second pair is "2" and "3". Under $x\%$ split, the training/test sets of task 1 would have $(x/2+50)\,\%$ images from the first pair and $(-x/2+50)\,\%$ from the second pair, whereas task 2 would have $(-x/2+50)\,\%$ from the first and $(x/2+50)\,\%$ from the second. Images in both tasks are labeled according to the rule "0", "1" vs. "2", "3". Under 0% split the two datasets would be identical.

7. Exponential Fitting of Long-Term Forgetting

All exponential fitting of forgetting $F_{t,1}$was carried out on the forgetting averaged over randomness of task sequence generation. In the target-distractor task sequence, the randomness refers to the randomly selected subsets of images and the random labels in each task (see SI 5). For benchmark task sequences, it refers factors such as different subsamples of the full source dataset (SI 6). For permutation task sequences with very low permutation ratios, we sometimes observed a non-monotonic relation between averaged $F_{t,1}$ and t. In these cases we truncated the averaged $F_{t,1}$ at the maximum before fitting. In Fig. 3 and Fig. S1, we presented results averaged over 40 random seeds for the target-distractor task sequence, and in Figs. 4, S2, S5 we presented results averaged over 50 random seeds for the benchmark task sequences.

8. Gradient Descent Simulations

A. Single-Head CL. We performed numerical simulations using gradient descent dynamics with different types of regularizers and compared with our theoretical results.

A.1. Gradient Descent. For networks trained with vanilla gradient descent dynamics (GD), the dynamics is simply given by

$$\Theta_t^\tau = \Theta_t^{\tau-1} - \eta \nabla_{\Theta_t^{\tau-1}} \mathcal{L}(f_t(\Theta_t^{\tau-1}), D_t) \qquad [168]$$

here we use t to denote the index of the task, and τ to denote the time steps during training. For the first task the weights are initialized as $\Theta_1^0 \sim \mathcal{N}(0, \sigma_0^2)$, for the following tasks the weights Θ_t^0 are initialized as Θ_{t-1}, i.e., the network weights after learning task $t-1$. The learning process is run until the MSE on the current training data is less than 10^{-3}.

A.2. L-2 regularizer. For networks trained with an L-2 regularizer, the dynamics is given by

$$\Theta_t^\tau = \Theta_t^{\tau-1} - \eta \nabla_{\Theta_t^{\tau-1}} \mathcal{L}(f_t(\Theta_t^{\tau-1}), D_t) - \eta\kappa(\Theta_t^{\tau-1} - \Theta_{t-1}) \qquad [169]$$

where Θ_{t-1} denotes the network weights after finishing learning task $t-1$. Similarly, for the first task the weights are initialized as $\Theta_1^0 \sim \mathcal{N}(0, \sigma_0^2)$ and for the following tasks the weights Θ_t^0 are initialized as Θ_{t-1}. For the first task, the learning process was run without the regularizer, and ends when the MSE on the current training data is less than 10^{-3}. For the following tasks, the learning process is run sufficiently long, in order to approximate the $\lambda \to \infty$ solution where the closest solution to $\mathcal{W}_{t-1}$ is obtained.

A.3. Online EWC. For networks trained with online EWC, the dynamics is given by

$$\Theta_t^\tau = \Theta_t^{\tau-1} - \eta \nabla_{\Theta_t^{\tau-1}} \mathcal{L}(f_t(\Theta_t^{\tau-1}), D_t) - \eta\kappa \bar{F}_t \otimes (\Theta_t^{\tau-1} - \Theta_{t-1}) \qquad [170]$$

$$\bar{F}_t = \gamma \bar{F}_{t-1} + F_t; F_1 = 0 \qquad [171]$$

where F_t denotes the diagonal elements of the normalized fisher information matrix at time t,

$$F_t^i = \frac{\left(\frac{\partial \mathcal{L}(f_t(\Theta_t), D_t)}{\partial \Theta_t^i}\right)^2}{\frac{1}{N_{\text{params}}}\sqrt{\sum_{i=1}^{N_{\text{params}}}\left(\frac{\partial \mathcal{L}(f_t(\Theta_t), D_t)}{\partial \Theta_t^i}\right)^4}} \quad [172]$$

where N_{params} denotes the total number of parameters in the network. F_t (and therefore $\bar{F}_t$) has the same number of elements as Θ_t, and $\otimes$ denotes element-wise product between $\bar{F}_t$ and $\Theta_t^{\tau-1} - \Theta_{t-1}$. The decay parameter γ controls how much information about the old tasks is preserved, at $\gamma = 1$, the fisher information of all previous tasks contributes equally, and at $\gamma = 0$, only the fisher information of the last task is retained. The initialization Θ_t^0 and the stopping criteria for each task are the same as in SI 8.A.2.

B. Multi-Head CL. We performed numerical simulations using gradient descent dynamics with an explicit L-2 regularizer to validate the phase transitions predicted by our theory. To capture multi-head CL behaviors in the $\lambda \to \infty$ limit, we first trained an NN with GD on the first task until the network reaches zero training error. We added an explicit $L-2$ regularizer penalizing the change in hidden-layer weights as we proceed to learn the next tasks. The dynamics is given by

$$\mathcal{W}_t^\tau = \mathcal{W}_t^{\tau-1} - \eta \nabla_{\mathcal{W}_t^{\tau-1}} \mathcal{L}(f_t^t(\Theta_t^{\tau-1}), D_t) - \eta\kappa(\mathcal{W}_t^{\tau-1} - \mathcal{W}_{t-1}) \quad [173]$$

$$a_t^\tau = a_t^{\tau-1} - \eta \nabla_{a_t^{\tau-1}} \mathcal{L}(f_t^t(\Theta_t^{\tau-1}), D_t) \quad [174]$$

For each task, the readout weights are always initialized as $a_t^0 \sim \mathcal{N}(0, \sigma_0^2)$, where as the hidden-layer weights $\mathcal{W}_t^0$ are initialized as $\mathcal{W}_{t-1}$, which denotes the weights obtained at the end of training on the previous task. Except for on the first task, $\mathcal{W}_1^0$ is initialized as $\mathcal{W}_1^0 \sim \mathcal{N}(0, \sigma_0^2)$. The learning process is run sufficiently long, in order to approximate the $\lambda \to \infty$ limit where the closest solution to $\mathcal{W}_{t-1}$ is obtained. Results are shown in Figs. S7, S8**d**,**g**, S9**b**,**c**.

Part III

Additional numerical results

9. Numerical results on single-head CL

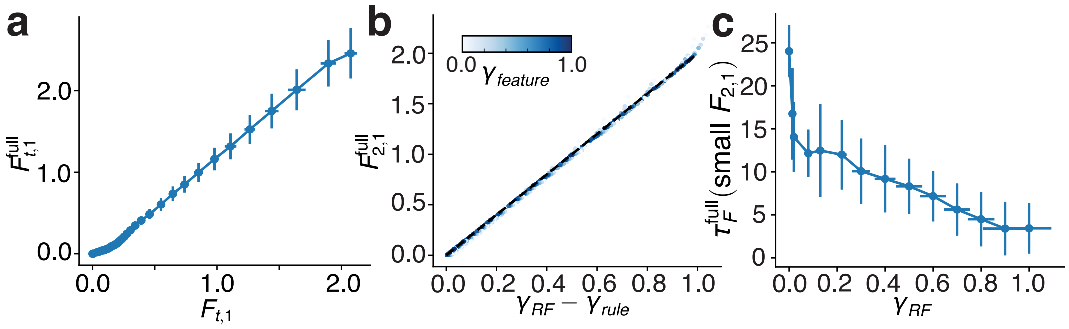

Fig. S1. Comparison between results under the full Gibbs distribution and the random feature model.
a Forgetting evaluated using the full theory ($F^{\text{full}}_{t,1}$, SI Eq. 16) vs. the random feature approximation ($F_{t,1}$) for t from 1 to T. The full theory behaves similarly to the random feature approximation across a wide range of t and task relations.
b Short-term forgetting evaluated using the full theory ($F^{\text{full}}_{2,1}$) behaves similarly as $F_{2,1}$ evaluated using the random feature approximation. $F^{\text{full}}_{2,1}$ can also be accurately captured by the interference $\gamma_{\text{RF}} - \gamma_{\text{rule}}$, and is independent of γ_{feature}.
c Long-term forgetting evaluated using the full theory also behaves similarly as the random feature approximation. We focus on the time constant τ_F^{full} for task sequences with small $F^{\text{full}}_{2,1}$ as in Fig. 3 in the main text. τ_F^{full} also decreases with γ_{RF}. The decrease is fast when γ_{RF} is close to 0 and slower for larger γ_{RF}.
All results were evaluated on the target-distractor task sequences with the same parameters as in Fig. 3, and detailed in SI 5. Results were averaged over 40 random seeds for task sequence generation.

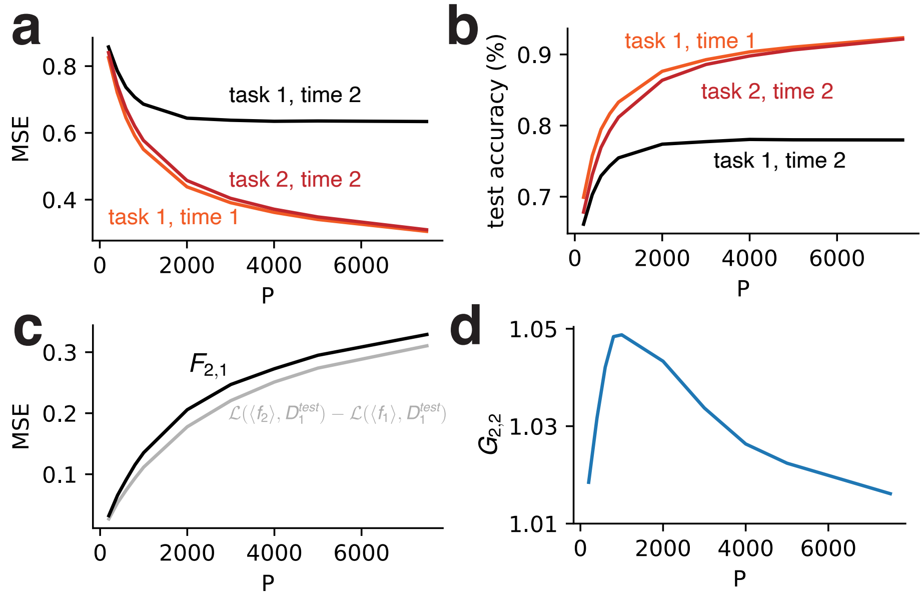

Fig. S2. Generalization in single-head CL and the choice of P.

a MSE on the test data of task 1 and task 2, measured after learning task 2. For learning single tasks (task 1, time 1), or learning the current task (task 2, time 2), generalization error improves with P. For the previously learned task (task 1, time 2), generalization error plateaus at around $P = 2000$, which is the value of P we selected for presenting all results in the main text.

b Same as **a**, but showing the test accuracy. The accuracy is measured by the percentage of the mean input-output mappings $\langle f_T\rangle$ that have the same sign as the labels.

c Short-term forgetting measured on the training data ($F_{2,1}$) increases with P. The behavior of $F_{2,1}$ is similar to forgetting measured using the generalization error, i.e., the difference between the generalization error on task 1 after learning task 2 ($\mathcal{L}(\langle f_2\rangle, D_1^{\text{test}})$) and the generalization error of learning task 1 alone ($\mathcal{L}(\langle f_1\rangle, D_1^{\text{test}})$).

d The anterograde effect is measured by $G_{2,2} \equiv \mathcal{L}(\langle f_2\rangle, D_2^{\text{test}})/G_2^0$, where G_2^0 is the generalization error when learning task 2 alone. $G_{2,2}$ remains close to 1 across all values of P, suggesting that the anterograde effect is weak.

Results were computed on permuted MNIST with 100% permutation ratio using the random feature approximation, in a network with $L = 9$, and averaged over 50 random seeds for task sequence generation.

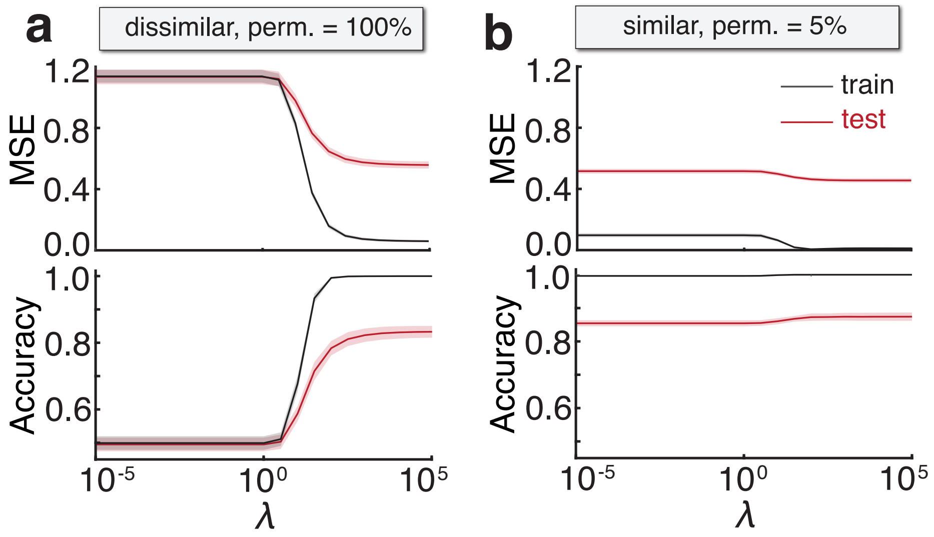

Fig. S3. Dependence of CL performance on λ.

a MSE and accuracy on both train (black) and test (red) data on the first task, after learning two tasks, for permuted MNIST with 100% permutation ratio. λ has a strong effect in mitigating forgetting. MSE on both train and test data decreases significantly with λ, and accuracy increases.

b Same as **a**, but for permuted MNIST with 5% permutation ratio. The effect of λ in mitigating forgetting is small, MSE remains small, and accuracy remains high across all values of λ.

The result suggests that λ plays a crucial role in mitigating forgetting in dissimilar tasks, but is less effective in similar tasks, where forgetting is already small without any regularizers, consistent with Fig. 3**a** inset in the main text. Shaded errorbars are standard deviations across 10 random seeds for task sequence generation. Both MSE and accuracy are evaluated using the mean input-output mappings. The accuracy is measured by the percentage of the mean input-output mappings ($\langle f_T\rangle$) that have the same sign as the labels. Results were computed with $L = 9$ and $P = 2000$, using the mean input-output mappings over the full Gibbs distribution.

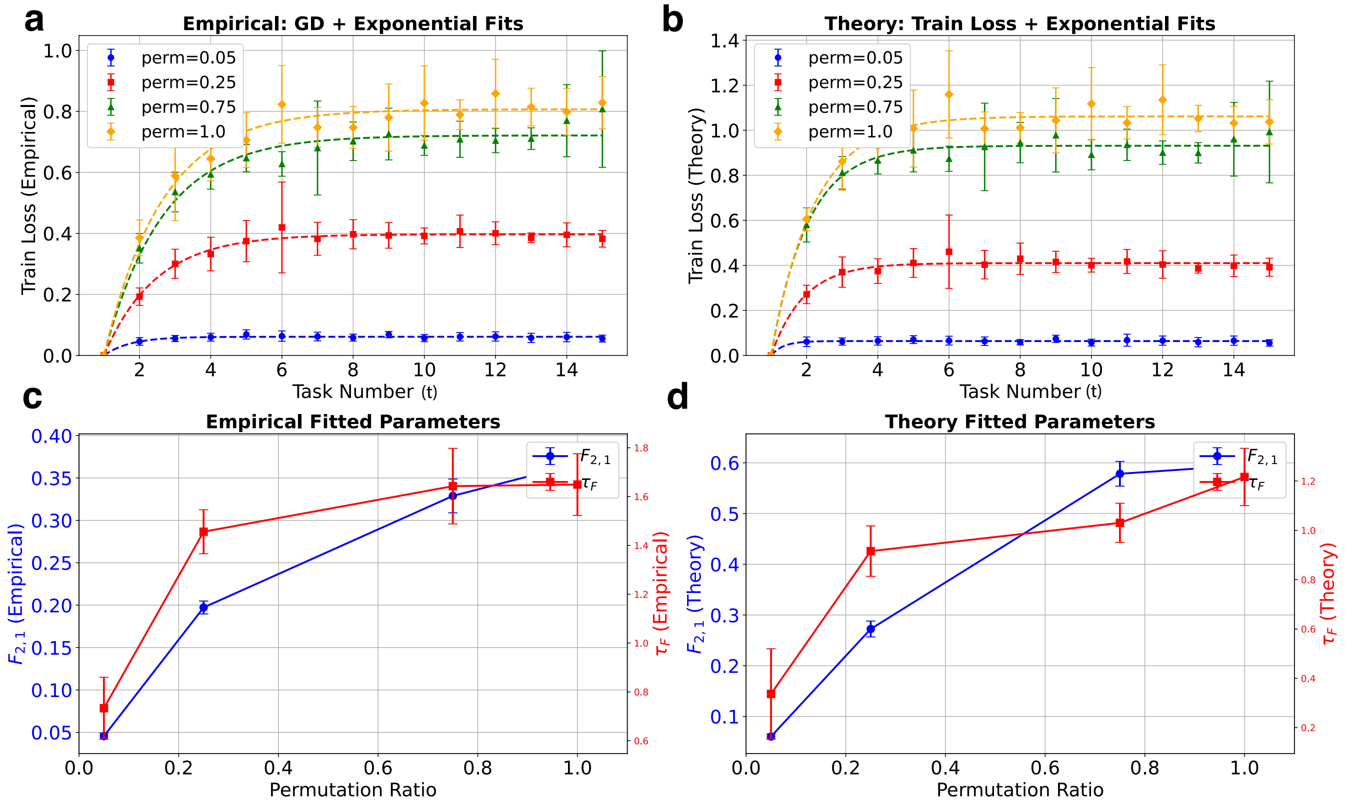

Fig. S4. Forgetting in networks trained with gradient descent on permuted MNIST task, compared with the theoretical result.
a Forgetting on the first task ($F_{t,1}$) as a function of number of task learned (t) in networks trained on permuted MNIST with different permutation ratios. Dashed lines show exponential fit of $F_{t,1}$.
b Same as **a**, but showing forgetting ($F_{t,1}$) predicted by the theory as presented in Section E.
c Parameters of the exponential fit including short-term forgetting ($F_{2,1}$) and the relaxation time constant τ_F as a function of the permutation ratio. Both increases monotonically as permutation ratio increases.
d Same as **c**, but showing the parameters of the exponential fit estimated using the theory.
Details of the training process are in SI 8.A. For both GD and theory, errorbars are standard errors across 10 random seeds of both task sequence generation. For GD result, the mean predictor used to compute $F_{t,1}$ is estimated by averaging over 10 random seeds for network initialization. Results were obtained with $L = 1$ and $P = 2000, N = 2000$ on the permuted MNIST task sequence with different permutation ratios, theoretical results were calculated with the random feature approximation, GD simulations were done with $\eta = 0.001, \kappa = 0.1, \sigma_0 = 1$.

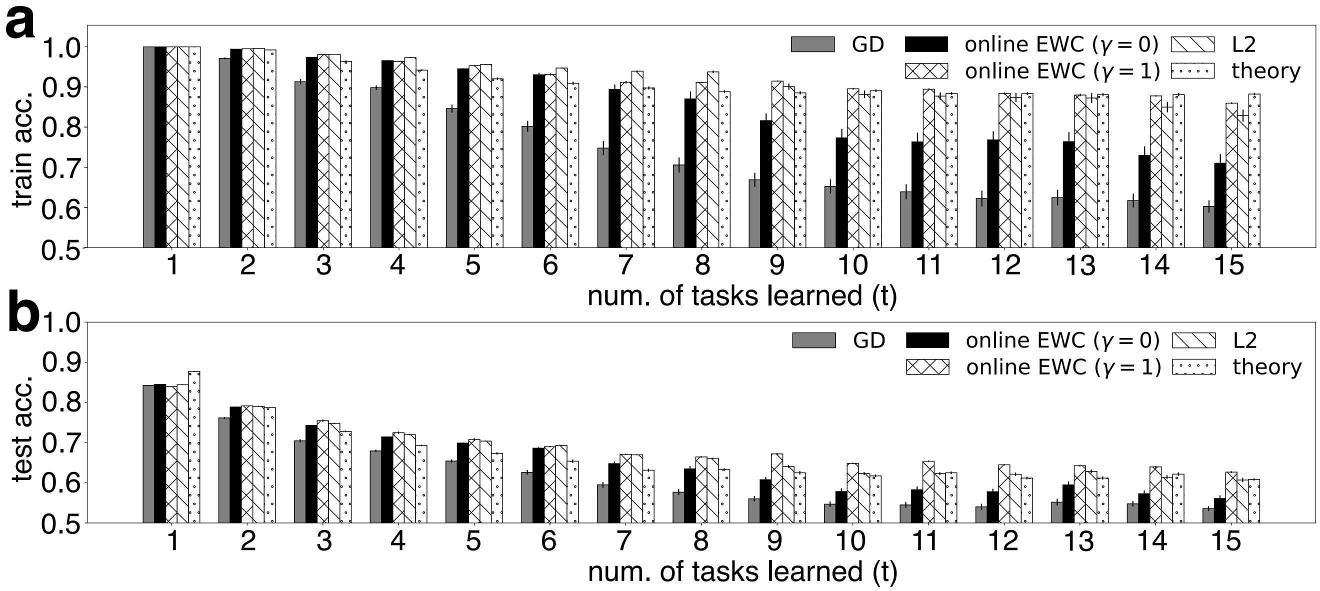

Fig. S5. Forgetting in networks trained with gradient descent with different regularizers in single-head CL.
a Train accuracy on the first task after learning t tasks, for t ranging from 1 to 15. We evaluated the accuracy for networks trained with vanilla gradient descent without explicit regularizers (GD), with online EWC with different decay parameters (online EWC ($\gamma = 0$) and online EWC ($\gamma = 1$)) or with an $L-2$ regularizer (L2), and compared their performance with accuracy obtained using our theoretical result of $\langle f_T \rangle$. Our theory achieves comparable performance L2 regularizer and online EWC with $\gamma = 1$, and outperforms vanilla GD and online EWC with $\gamma = 0$.
b Same as **a**, but showing the test accuracy.
Details of the training process are in SI 8.A. For the numerics (GD, online EWC ($\gamma = 0$ and $\gamma = 1$), L2), errorbars are standard errors across 100 random seeds of both task sequence generation and initialization. The theory is evaluated using the mean input-output mapping, and the accuracy is measured by the percentage of the mean input-output mappings ($\langle f_T \rangle$) that have the same sign as the labels. The errorbars are standard errors across 50 random seeds of task sequence generation. Results were obtained with $L = 9$ and $P = 2000$ on the permuted MNIST task sequence with permutation ratio 100%, theoretical results were calculated with the random feature approximation, GD simulations with different regularizers were done with $\eta = 0.001$, $\kappa = 0.1$, $\sigma_0 = 1$.

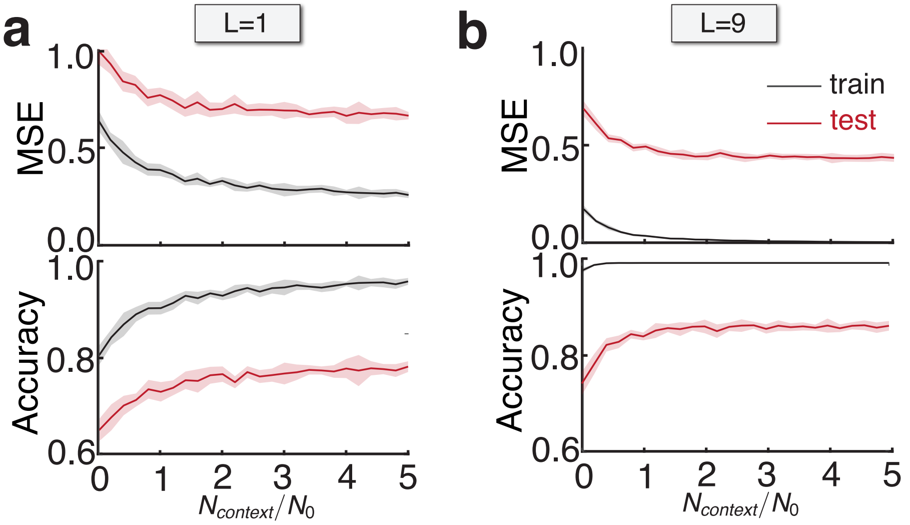

Fig. S6. Incorporating task-identity information in single-head CL. a MSE and accuracy measured on the train and test data on the first task after learning two tasks, as a function of the relative context length N_{context}/N_0, in a network with $L = 1$. Forgetting performance measured with both train and test data improves significantly with the context length. $F_{2,1}$ goes to a finite value larger than 0 as the context length increases. The task identity information is incorporated by appending an N_{context}-dimensional task-dependent context vector to each N_0-dimensional input. The context vector is drawn from an i.i.d. Gaussian distribution of dimension N_{context}, and is re-sampled for each new task.
b Same as **a**, but for a network with $L = 9$.
The results show that effectiveness of incorporating task-identity information in mitigating forgetting is stronger in shallower networks, but still less effective than using multi-head CL (where forgetting can be 0). Forgetting in deeper networks is already small without incorporating task-identity information. Results were evaluated on the permuted MNIST task sequence with permutation ratio 100% and $P = 2000$, using the random feature approximation of the mean input output mappings. Accuracy is measured by the percentage of the mean input-output mappings that have the same sign as the labels. Errorbars are across 10 random seeds of both task sequence generation and sampling of the context vectors.

813 ## 10. Numerical results on multi-head CL

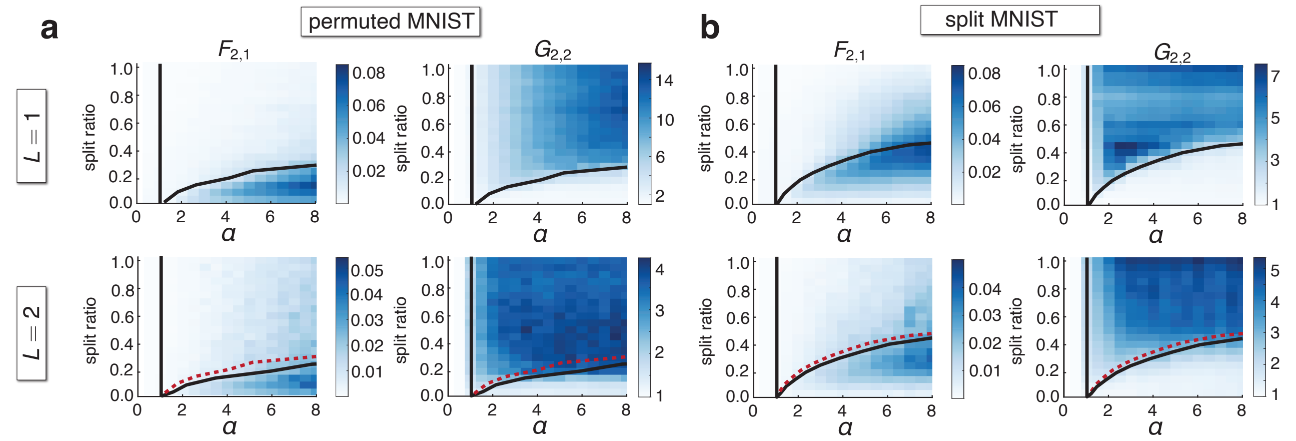

Fig. S7. Phase transitions in networks trained with gradient descent and explicit L-2 regularizer.
a $F_{2,1}$ (left) and $G_{2,2}$(right) evaluated from networks trained with GD and an explicit L-2 regularizer (SI Eqs. 174, 173), as a function of the permutation ratio and α for permuted MNIST, for $L = 1$(top) and $L = 2$(bottom). The same 3 regimes are observed as predicted by the theory. In networks with $L = 1$, theoretical approximation of the phase-transition boundary (black line) accurately matches the simulation. In networks with $L = 2$, the same phase transitions are observed, and the overfitting regime slightly extends beyond the theoretical phase-transition boundary of $L = 1$ (red dashed line), indicating a slightly stronger anterograde interference effect. Simulations are done with $\kappa = 0.1$, $\sigma_0 = 1$, $\eta = 0.01$ for $L = 1$ and $\eta = 0.001$ for $L = 2$ and $P = 600$.
b Same as a, but on the split MNIST task sequence. Simulations are done with $\kappa = 0.1$, $\sigma_0 = 1$, $\eta = 0.005$ for $L = 1$ and $\eta = 0.001$ for $L = 2$ and $P = 600$.
Results were averaged over 10 random seeds for initialization.

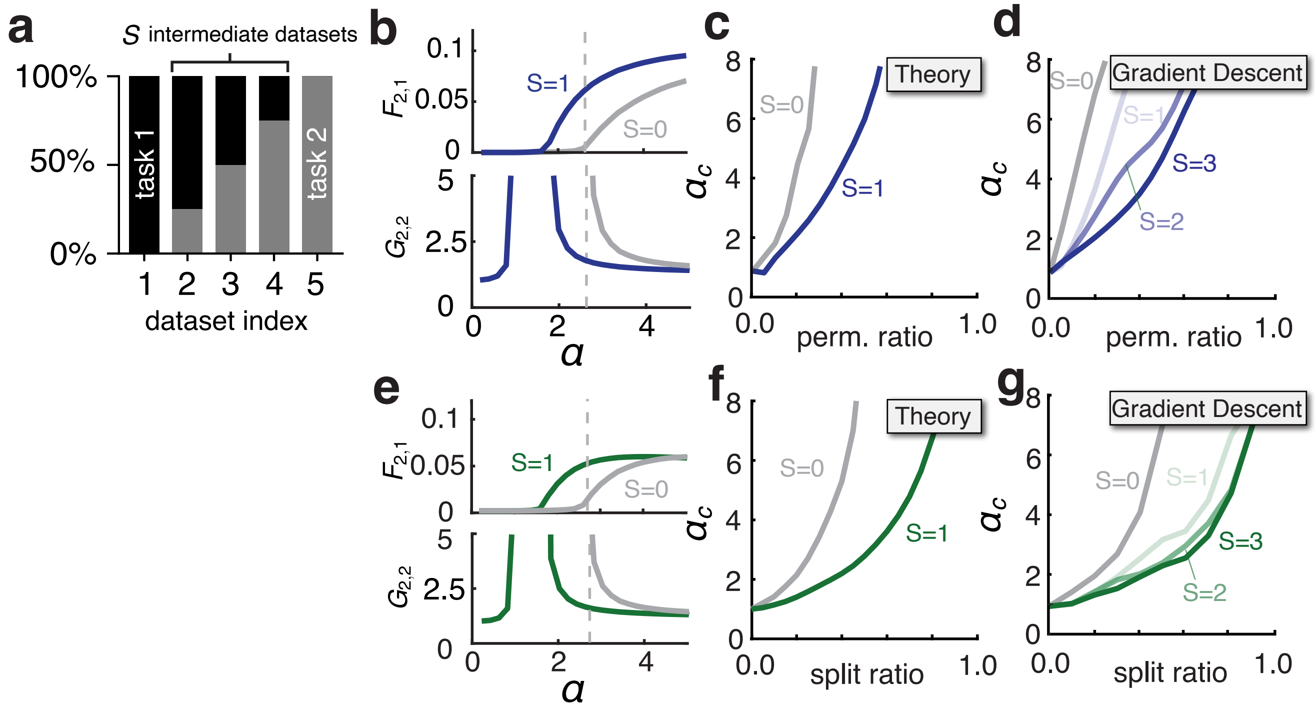

Fig. S8. Intermediate datasets help mitigate anterograde interference.

a Schematics of a task sequence with intermediate datasets. The first and last datasets solely contain training data from the first and second tasks, respectively, whereas the S intermediate ones contain a mixture of training data from two tasks, with the fraction of examples from task 2 increasing incrementally and equally across these datasets.

b Forgetting on the first task ($F_{2,1}$) and normalized generalization error on the second task ($G_{2,2}$) as a function of the network load α without intermediate datasets ($S = 0$, gray) and with one intermediate dataset ($S = 1$, blue) for a fixed pair permuted MNIST task sequence with permutation ratio 15%. In both cases there exists a critical α_c, for $\alpha > \alpha_c$ the network is in the generalization regime, $F_{2,1}$ is nonzero, and $G_{2,2}$ is finite; for $1 \leq \alpha \leq \alpha_c$, the network is in the overfitting regime, $F_{2,1} = 0$ and $G_{2,2}$ diverges. The critical α_c becomes smaller with the addition of an intermediate dataset, indicating a smaller overfitting regime and mitigated anterograde interference. Dashed line: theoretical prediction of α_c for $S = 0$.

c The critical α_c given by the theory, as a function of the permutation ratio in permuted MNIST. For task sequences without any intermediate datasets ($S = 0$), α_c was calculated by evaluating SI Eq. 97. For task sequences with 1 intermediate dataset ($S = 1$), α_c was calculated by estimating the position where $G_{2,2}$ starts to diverge (using $G_{2,2}(\alpha_c) = \frac{1}{2}(\max_{\alpha>1} G_{2,2}(\alpha) + \min_{\alpha>1} G_{2,2}(\alpha))$). The difference between the two lines $S = 0$ and $S = 1$ is larger as the permutation ratio increases, indicating that the benefit of intermediate datasets is more significant when task 1 and task 2 are less similar.

d The critical α_c given by gradient descent numerics with an explicit L-2 regularizer (SI Eqs. 173, 174), as a function of the permutation ratio in permuted MNIST, for $S = 0 - 3$. α_c was calculated by estimating the position where $G_{2,2}$ starts to diverge in the gradient descent simulations (using $G_{2,2}(\alpha_c) = \frac{1}{2}(\max_{\alpha>1} G_{2,2}(\alpha) + \min_{\alpha>1} G_{2,2}(\alpha))$ as in **c**) The effect of mitigating forgetting increases with the number of intermediate datasets S.

e-**g** Same as **b**-**d**, but for split MNIST. In **e** the split ratio is 25%.

Results were computed with $P = 600$ and $L = 1$. **d** and **g** were calculated with $\kappa = 0.1$, $\sigma_0 = 1$, $\eta = 0.01$, and averaged over 10 random seeds for initialization.

11. Numerical comparison between single-head and multi-head CL

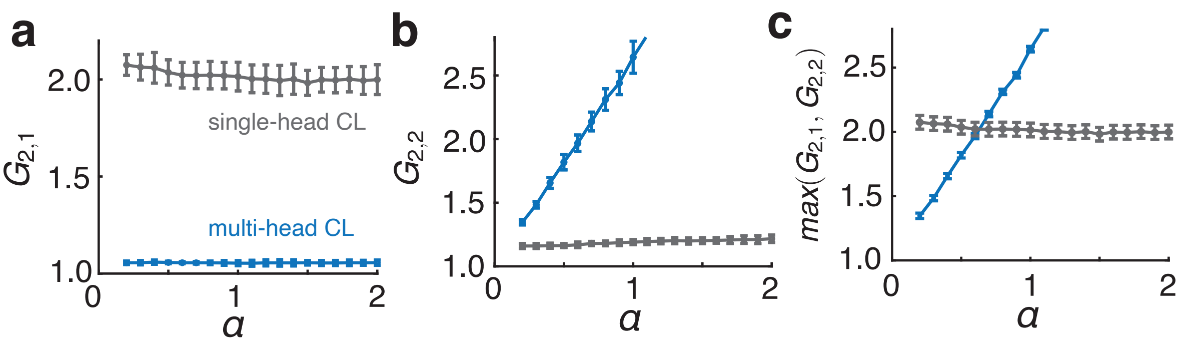

Fig. S9. Empirical comparison between single-head and multi-Head CL.

a Normalized generalization error on the first task after sequentially learning two tasks for single-head ($G_{2,1} = \left\langle \mathcal{L}(f_2, D_1^{test}) \right\rangle / G_1^0$) and multi-head ($G_{2,1} = \left\langle \mathcal{L}(f_2^1, D_1^{test}) \right\rangle / G_2^0$) CL as a function of the load α (α is changed through modifying N and fixing P). Multi-head CL always outperforms single-head CL for the entire range of α.

b Normalized generalization error on the second task after sequentially learning two tasks for single ($G_{2,2} = \left\langle \mathcal{L}(f_2, D_2^{test}) \right\rangle / G_2^0$) and multi-head ($G_{2,2} = \left\langle \mathcal{L}(f_2^2, D_2^{test}) \right\rangle / G_2^0$) CL as a function of α. The normalized generalization error remains close to 1 for single-head CL, but increases significantly with α for multi-head CL.

c Same as **a**, **b**, but taking the maximum over $G_{2,1}$ and $G_{2,2}$ in order to compare the overall performance on both tasks.

Numerics are done using split MNIST dataset with 100% split ratio, such that multi-head CL lies in the overfitting regime as long as $\alpha > 1$. Detailed parameters are $P = 600, \gamma = 0.1, \eta = 0.005$ and $\sigma_0 = 0.5$ for both scenarios.

References

1. A Jacot, F Gabriel, C Hongler, Neural tangent kernel: Convergence and generalization in neural networks. Adv. neural information processing systems **31** (2018).
2. J Lee, et al., Deep neural networks as gaussian processes. arXiv preprint arXiv:1711.00165 (2017).
3. T Hofmann, B Schölkopf, A Smola, Kernel methods in machine learning. The Annals Stat. **36**, 1171–1220 (2008).
4. Y Cho, L Saul, Kernel methods for deep learning. Adv. neural information processing systems **22** (2009).
5. Y Avidan, Q Li, H Sompolinsky, Connecting ntk and nngp: A unified theoretical framework for neural network learning dynamics in the kernel regime. arXiv preprint arXiv:2309.04522 (2023).
6. Q Li, H Sompolinsky, Statistical mechanics of deep linear neural networks: The backpropagating kernel renormalization. Phys. Rev. X **11**, 031059 (2021).
7. S Franz, G Parisi, Recipes for metastable states in spin glasses. J. de Physique I **5**, 1401–1415 (1995).
8. A Krizhevsky, G Hinton, Learning multiple layers of features from tiny images, (University of Toronto), Technical Report TR-2009 (2009).
9. KE O'Grady, Measures of explained variance: Cautions and limitations. Psychol. Bull. **92**, 766 (1982).
10. Y LeCun, L Bottou, Y Bengio, P Haffner, Gradient-based learning applied to document recognition. Proc. IEEE **86**, 2278–2324 (1998).
11. G Cohen, S Afshar, J Tapson, A Van Schaik, Emnist: Extending mnist to handwritten letters in 2017 international joint conference on neural networks (IJCNN). (IEEE), pp. 2921–2926 (2017).
12. H Xiao, K Rasul, R Vollgraf, Fashion-mnist: a novel image dataset for benchmarking machine learning algorithms. arXiv preprint arXiv:1708.07747 (2017).
13. IJ Goodfellow, M Mirza, D Xiao, A Courville, Y Bengio, An empirical investigation of catastrophic forgetting in gradient-based neural networks. arXiv preprint arXiv:1312.6211 (2013).
14. F Zenke, B Poole, S Ganguli, Continual learning through synaptic intelligence in International conference on machine learning. (PMLR), pp. 3987–3995 (2017).
